# Randomised controlled trial of an augmented exercise referral scheme using web-based behavioural support for inactive adults with chronic health conditions: the e-coachER trial

**DOI:** 10.1136/bjsports-2020-103121

**Published:** 2020-11-27

**Authors:** Adrian Taylor, Rod S Taylor, Wendy Ingram, Sarah Gerard Dean, Kate Jolly, Nanette Mutrie, Jeff Lambert, Lucy Yardley, Adam Streeter, Colin Greaves, Chloe McAdam, Lisa Price, Nana Kwame Anokye, John Campbell

**Affiliations:** 1 Peninsula School of Medicine, Faculty of Health, University of Plymouth, Plymouth, Devon, UK; 2 Institute of Health and Wellbeing, University of Glasgow, Glasgow, UK; 3 Health Statistics, University of Exeter Medical School, Exeter, UK; 4 Peninsula Clinical Trials Unit, University of Plymouth, Plymouth, Devon, UK; 5 University of Exeter Medical School, Exeter, Devon, UK; 6 Public Health, Epidemiology and Biostatistics, University of Birmingham, Birmingham, UK; 7 Physical Activity for Health Research Centre, The University of Edinburgh, Edinburgh, UK; 8 Department of Health, University of Bath, Bath, Somerset, UK; 9 School of Psychological Sciences, University of Bristol, Bristol, UK; 10 Medical Statistics, University of Plymouth, Plymouth, Devon, UK; 11 School of Sport, Exercise and Rehabilitation Sciences, University of Birmingham, Birmingham, UK; 12 University of Exeter Medical School, Exeter, UK; 13 School of Sport and Health Sciences, University of Exeter, Exeter, Devon, UK; 14 Department of Clinical Sciences, Brunel University, Uxbridge, Middlesex, UK

**Keywords:** accelerometer, physical activity, behaviour, chronic, primary care

## Abstract

**Objective:**

To determine whether adding web-based support (e-coachER) to an exercise referral scheme (ERS) increases objectively assessed physical activity (PA).

**Design:**

Multicentre trial with participants randomised to usual ERS alone (control) or usual ERS plus e-coachER (intervention).

**Setting:**

Primary care and ERS in three UK sites from 2015 to 2018.

**Participants:**

450 inactive ERS referees with chronic health conditions.

**Interventions:**

Participants received a pedometer, PA recording sheets and a user guide for the web-based support. e-coachER interactively encouraged the use of the ERS and other PA options.

**Main outcome measures:**

Primary and key secondary outcomes were: objective moderate-to-vigorous PA (MVPA) minutes (in ≥10 min bouts and without bouts), respectively, after 12 months. Secondary outcomes were: other accelerometer-derived and self-reported PA measures, ERS attendance, EQ-5D-5L, Hospital Anxiety and Depression Scale and beliefs about PA. All outcomes were collected at baseline, 4 and 12 months. Primary analysis was an intention to treat comparison between intervention and control arms at 12-month follow-up.

**Results:**

There was no significant effect of the intervention on weekly MVPA at 12 months between the groups recorded in ≥10 min bouts (mean difference 11.8 min of MVPA, 95% CI: −2.1 to 26.0; p=0.10) or without bouts (mean difference 13.7 min of MVPA, 95% CI: −26.8 to 54.2; p=0.51) for 232 participants with usable data. There was no difference in the primary or secondary PA outcomes at 4 or 12 months.

**Conclusion:**

Augmenting ERS referrals with web-based behavioural support had only a weak, non-significant effect on MVPA.

**Trial registration number:**

ISRCTN15644451.

## Introduction

Low levels of physical activity (PA) are a significant contributor to a wide range of chronic physical and mental health conditions such as obesity, type 2 diabetes, hypertension, osteoarthritis and depression^1–6^ and associated healthcare cost.^7^ Primary care exercise referral schemes (ERSs) have small positive effects on self-reported PA, compared with usual care. However, most of these trials are underpowered, and do not necessarily include physically inactive participants with chronic conditions.^8 9^ The format of ERS range from considerable exercise practitioner contact at an exercise facility to a signposting service to community PA options with minimal sustained contact.^10^ This variation in ERS makes a broader national approach to improving the quality of the patient experience and effectiveness challenging. Given that only 66%–81% ever attend the referral scheme, that only 43%–49% complete it^11^ and that the health benefits seem to be small,^12^ new ways are needed to improve uptake and adherence to ERS, and to foster sustainable PA from ERS.^13^

Web-based interventions have been shown to be effective in supporting short-term changes in (mostly self-reported) PA among the general population and those with clinical conditions.^14–18^ However, no studies have explored their use alongside ERS offering face-to-face support. Along with service users, we developed a bespoke support system called e-coachER, using the LifeGuide platform (https://www.lifeguideonline.org/), seeking to empower ERS participants with physical and mental health conditions to become more physically active and to remain motivated to do so. If shown to be an effective adjunctive intervention, such a system could be scaled up relatively cheaply and routinely offered to thousands of patients per year in hundreds of schemes in the UK.^10^

We undertook a multicentre parallel two-group randomised controlled trial to determine the impact of the addition of web-based behavioural support for ERS referral on PA and health outcomes in inactive people with chronic disease.

## Methods

The trial was conducted and reported in accordance with the Consolidated Standards of Reporting Trials guidelines.^19^ Our full trial protocol has been published elsewhere so we limit the details provided here.^20^

### Study population

Between July 2015 to March 2017, we recruited low active adults with at least one chronic condition (from obesity, hypertension, type 2 diabetes, lower limb osteoarthritis and depression) in Greater Glasgow, Birmingham or Plymouth and adjacent rural areas, who had been or were about to be referred by a primary care practitioner to a local ERS. For a full list of inclusion/exclusion criteria see online supplemental material (online supplemental appendix 1). For a full list of ways in which participants were recruited see online supplemental material (online supplemental appendix 2).

10.1136/bjsports-2020-103121.supp1 Supplementary data

10.1136/bjsports-2020-103121.supp2 Supplementary data

### Study procedures

A summary of the recruitment procedures is shown in a flow chart in online supplemental material appendix 3, and full procedures were previously reported.^20^

10.1136/bjsports-2020-103121.supp3 Supplementary data

### Randomisation and blinding

Participants were randomly allocated 1:1 to either usual ERS alone (control arm) or usual ERS plus e-coachER (intervention arm). Randomisation was stratified by site with minimisation by the participant’s perceived main reason for their referral to the ERS (ie, chronic condition) and by self-reported IT literacy/confidence using a 10-point scale.

Blinding to trial allocation among the trial statistician and most of the research team (excepting those involved in the qualitative process evaluation) was not broken until the primary and secondary analyses were reported to the Project Management Group.

### Intervention

Participants allocated to the intervention group were mailed a small box containing a user guide for accessing the e-coachER web-based support system, a pedometer (step-counter) and a fridge magnet with tear-off sheets to record weekly step counts or minutes of moderate-to-vigorous PA (MVPA). The user guide provided a summary of the content on the website and guidance on how to register to access a range of interactive opportunities to enhance participants’ motivation to take up the ERS and to become more physically active, whether or not they engaged with their local ERS. A logic model for the intervention and a more detailed description of the content, in compliance with the TiDIER checklist and Behaviour Change Techniques mapping has been reported elsewhere.^20^

The interactive e-coachER support system adopted effective features from other interventions.^21^ It involved seven ‘Steps to Health’ designed to take about 5–10 min each to complete each week. We defined getting to step 5 (setting a goal and reviewing a goal online) as a sufficient ‘dose’ of the intervention to impact on minutes of MVPA, although we recognise that merely mailing a pedometer could, for some, be an effective intervention.^22^

### Control

Participants in both arms of the trial were offered usual primary care ERS, across three different schemes, as described elsewhere,^20^ to increase the generalisability of the trial.

### Data collection

At 4 and 12 months post randomisation, participants were sent an accelerometer and questionnaire booklet by post, and prepaid envelope to return to Peninsula Clinical Trials Unit. Reminder letters and phone calls aimed to increase follow-up rates. Participants returning the device received an online/high street store voucher for £20 on each occasion.

### Outcomes measures

The primary outcome was the number of weekly minutes of MVPA, recorded in ≥10 min bouts, measured objectively by GENEActiv Original accelerometer (Activinsights; https://www.geneactiv.org/), over a 1-week period at 12 months post randomisation. A description of our procedures for processing accelerometer data is provided in online supplementary material appendix 4. Briefly, GENEActiv PC software (V.3.0_09.02.2015) was used with software R using package GGIR V.1.2-8 (https://cran.r-project.org/web/packages/GGIR/index.html)^23^ to identify data for the primary analysis if participants achieved a minimum of 16 hours of wear time for a minimum of 4 days (including at least 1 weekend day).

10.1136/bjsports-2020-103121.supp4 Supplementary data

Other accelerometer recorded and self-reported secondary outcomes at 4 and 12 months are shown in online supplemental material appendix 5 and table 1. Initial attendance at the ERS was captured from ERS providers with imputed participant-reported attendance at 4 weeks and/or 4 months where the ERS service data were missing. Engagement with the e-coachER intervention was captured using the LifeGuide platform. Other methods and data used for our health economic evaluation and process evaluation are reported elsewhere.^20^

10.1136/bjsports-2020-103121.supp5 Supplementary data

**Table 1:** Summary of secondary outcomes at baseline and 4 and 12 months follow-up and analysis of between group differences at 12 months

	Baseline		4-month follow-up		12-month follow-up		Between group difference or OR at 12 months* Mean (95% CI) P value
	Control Mean (SD) Median (IQR) or n/N (%)	Intervention Mean (SD) Median (IQR) or n/N (%)	Control Mean (SD) Median (IQR) or n/N (%)	Intervention Mean (SD) Median (IQR) or n/N (%)	Control Mean (SD) Median (IQR) or n/N (%)	Intervention Mean (SD) n, Median (IQR) or n/N (%)	
Achievement of at least 150 min of weekly MVPA in ≥10 min bouts†	8/201 (4%)	9/207 (4%)	2/128 (2%)	7/109 (6%)	3/133 (2%)	6/110 (5%)	OR: 3.80 (0.16 to 20.92), 0.12
Achievement of at least 150 min of weekly MVPA†	149/201 (74%)	178/207 (86%)	98/128 (76%)	99/109 (91%)	99/133 (74%)	93/110 (85%)	OR: 1.67 (0.82 to 3.42), 0.16
Self-reported MVPA weekly minutes	N=220, 213.5 (352.7) 65.0 (0–285.0)	N=220, 204.0 (375.6) 47.5 (0–247.5)	N=183, 318.0 (517.6) 95.7 (0–305.2)	N=166, 306.1 (430.5) 105.0 (0–314.1)	N=170, 228.3 (424.4) 85.0 (0–285.0)	N=154, 252.7 (426.2) 130.0 (0–320.2)	49.3 (−36.3 to 135.0) 0.26
Achievement of at least 150 min of weekly MVPA self-reported	83/220 (37%)	77/220 (48%)	94/183 (51%)	88/166 (53%)	76/170 (45%)	76/154 (49%)	OR: 1.23 (0.79 to 1.90), 0.36
Average daily diurnal inactivity (hours)†	N=199, 1.7 (1.1)	N=205, 1.5 (1.1)	N=125, 1.4 (1.1)	N=109, 1.4 (0.9)	N=99, 1.4 (1.0)	N=78, 1.5 (1.0)	0.6 (0.5 to 0.7),<0.0001
Average daily sleep (hours)†	N=199, 6.8 (1.5)	N=205, 6.9 (1.2)	N=125, 6.7 (1.3)	N=109, 6.7 (1.4)	N=128, 6.8 (1.5)	N=110, 7.0 (1.5)	0.3 (−0.1 to 0.6), 0.11
EQ-5D-5L (Devlin values)	N=216, 0.74 (0.24)	N=215, 0.76 (0.23)	N=162, 0.72 (0.26)	N=148, 0.76 (0.25)	N=158, 0.72 (0.26)	N=138, 0.73 (0.27)	0.00 (−0.4 to 0.05) 0.89
HADS-D	N=217, 7.6 (4.5)	N=214, 7.4 (4.7)	N=164, 7.4 (4.8)	N=147, 6.0 (4.7)	N=156, 7.1 (4.8)	N=139, 6.3 (5.1)	−0.2 (−1.0 to 0.6), 0.44
HADS-A	N=217, 8.7 (4.6)	N=214, 8.6 (5.1)	N=164, 8.5 (4.8)	N=146, 7.5 (5.0)	N=156, 8.4 (4.8)	N=139, 7.6 (5.2)	−0.5 (−1.2 to 0.2), 0.20

Median (IQR) reported for accelerometry and self-report continuous PA outcomes only.

*Adjusted for baseline MVPA, age, gender, site and minimisation variables.

†Non-bouted accelerometer recorded MVPA adjusted for baseline outcome value, age, gender, site and minimisation variables.

HADS-A, Hospital Anxiety & Depression Scale - Anxiety; HADS-D, Hospital Anxiety & Depression Scale - Depression; MVPA, moderate-to-vigorous physical activity.

### Statistical analysis

In the absence of a published minimally important difference for MVPA, we assumed a ‘small-to-moderate’ standardised effect size of 0.35, and estimated that 413 participants were required at 88% power and a two-sided alpha of 5% assuming for 20% attrition, or 90% power at a two-sided alpha of 5% allowing for 16% attrition (using ‘sampsi’ in STATA V.14.2). Based on the baseline SD for MVPA total weekly minutes in ≥10 min bouts of 104 to 113,^24^ an effect size of 0.35 would correspond to a mean between group differences of 36–39 min of MVPA per week at 12-month follow-up.

All statistical analyses were conducted to a predefined analysis plan prior to end of data collection and any comparison of follow-up outcomes. The primary analysis compared primary and secondary outcomes between groups in accordance with the principle of intention to treat (ITT) (ie, based on original random allocation) in participants with complete outcomes at 12 months, adjusting for baseline outcome values and stratification and minimisation variables. Following assessment of baseline demographics, mean age and gender were also added to the adjusted model.

Two secondary analyses were undertaken to compare groups across all follow-up points using a mixed model repeated measures approach and complier average causal effect (CACE) analyses undertaken to examine the impact of adherence to the intervention, (ie, (a) simply registering to access the website or not and (b) completing five or more ‘Steps to Health’ or not) on primary and secondary outcomes at 12 months.

The primary analysis model was extended to fit interaction terms to explore possible subgroup differences in intervention effect in stratification and minimisation variables for the primary outcome at 12 months. Given the low power for testing interactions, these results were treated only as exploratory. Sensitivity analyses were conducted for four additional wear time criteria (see online supplemental table appendix 6): Multiple imputation was used to replace missing outcome data using baseline outcomes and other explanatory covariates (eg, treatment group, age), assuming unobserved measurements were missing at random. Given that the proportion of patients with missing accelerometry data was <3% out of the total number of participants who fulfilled the wear time criteria of includable PA data (n=243), no imputation was undertaken for the accelerometry related primary and secondary outcome. Using the same primary analysis model as described above, between-group outcomes were compared in ITT complete case and imputed data sets for non-accelerometry related secondary outcomes at 12 months. All analyses were conducted by a blinded statistician using STATA V.14.2.

10.1136/bjsports-2020-103121.supp6 Supplementary data

### Patient and public involvement (PPI)

PPI representatives with diverse clinical conditions and experience of ERS provided critical feedback on the development and usability of the intervention, trial participant-facing documents, participant newsletter, on recruitment and trial retention issues, and interpretation of the findings and dissemination. Other stakeholders involved in the delivery of ERS such as managers and practitioners were also consulted in each site.

### Process evaluation and economic evaluation

Findings from an embedded process evaluation and economic evaluation will be presented elsewhere.

## Results

### Participant flow through the trial

**Figure 1:**
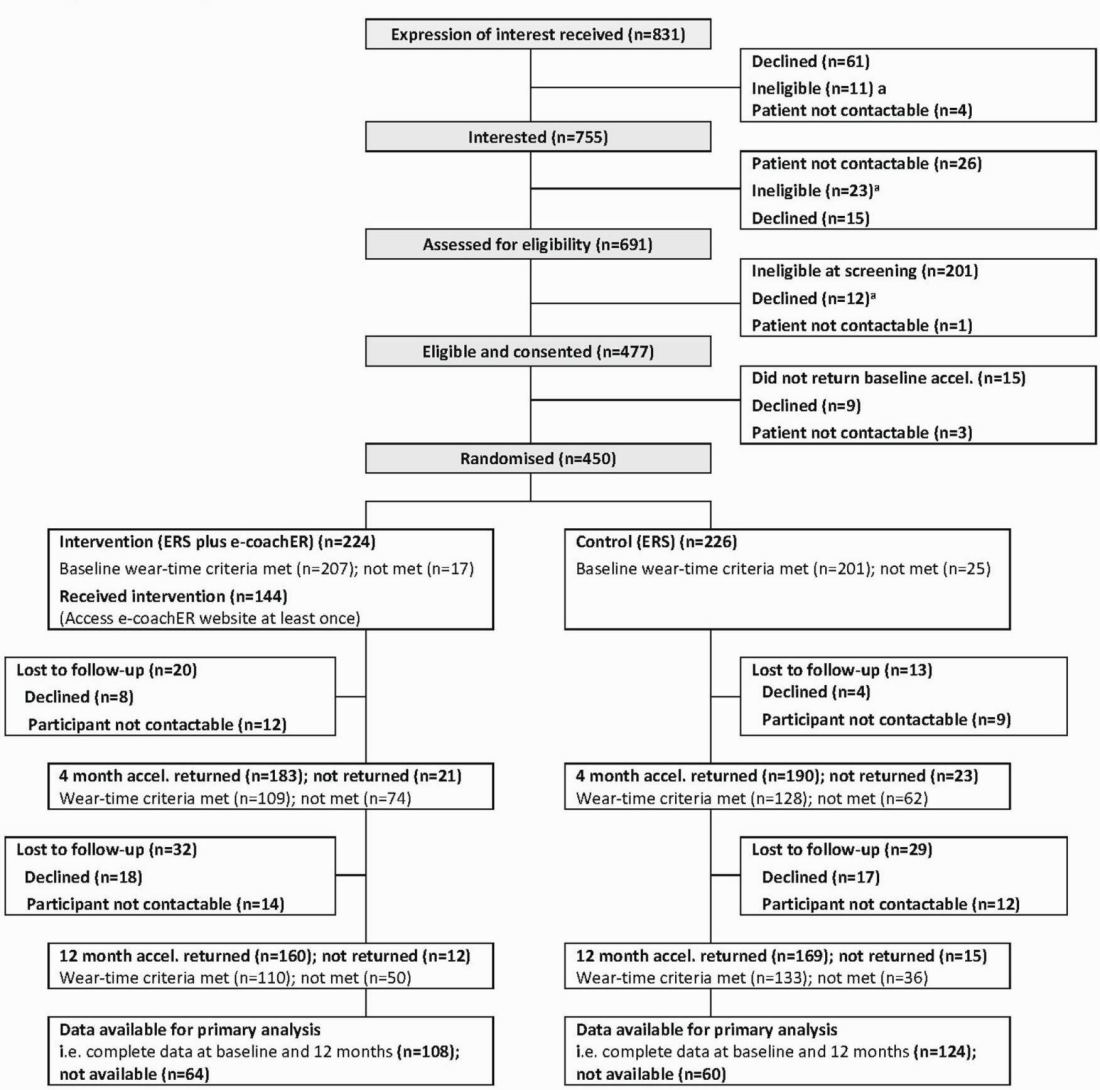
Participant flow chart. ^a^Reasons for ineligibility shown in online supplemental material appendix 7. ERS, exercise referral scheme.

Figure 1 shows the flow of participants through the trial. The reasons for ineligibility at each stage of recruitment are shown in online supplemental material appendix 7. Of the 450 participants randomised, 232 met our pre set, primary outcome wear time threshold (at baseline and 12 months). There was no evidence of differences in the demographic characteristics of those participants who provided primary outcome data at 12 months compared with those that did not provide this follow-up data.

10.1136/bjsports-2020-103121.supp7 Supplementary data

### Baseline participant characteristics

Table 2 shows the baseline characteristics of the 450 randomised participants as a whole and by trial arm. While in general the two arms were well balanced, we noted some small differences within categories in respect of education status though numbers in each category were small.

**Table 2:** Baseline characteristics by study group and for the whole sample (N=450 unless stated)

	Control group	Intervention	Both groups
N	226	224	450
Gender—n male (%)	84 (37)	76 (34)	160 (36)
Age—mean (SD) (range)	51 (14) (18–75)	50 (13) (20–73)	50 (12) (18–75)
BMI—mean (SD) (range)	32.5 (4.4) (18.8–40.5)	32.7 (4.5) (18.8–40.4)	32.6 (4.4) (18.8–40.5)
GP PAQ Score—n (%)			
2 (inactive)	144 (63.7%)	149 (66.5%)	293 (65.1%)
3 (moderately inactive)	82 (36.3%)	75 (33.5%)	157 (34.9%)
Ethnicity—n (%)			
White	195 (86.3%)	179 (79.9%)	374 (83.1%)
South Asian	11 (4.9 %)	16 (7.2 %)	28 (6.2 %)
Other	20 (8.8 %)	28 (12.6 %)	48 (10.7 %)
Relationship status—n (%)			
Single, widowed, divorced, or dissolved or surviving civil partnership	124 (54.9%)	112 (50%)	236 (52.4%)
Married or civil partnership	102 (45.1%)	112 (50%)	214 (47.6%)
Domestic residence status (live with …)—n (%)			
Live alone	59 (26.1%)	48 (21.4%)	107 (23.8%)
Live with others (eg, parent, child, other family or non-family member or partner)	167 (73.9%)	176 (78.6%)	343 (76.2%)
Education status—n (%)			
No qualifications	52 (23.0%)	29 (12.9%)	81 (18.0%)
GCEs	146 (64.6%)	162 (72.3%)	308 (68.4%)
A-level	71 (31.4%)	96 (42.9%)	167 (37.1%)
First degree or above	58 (25.6%)	74 (33%)	132 (29.3%)
Other	108 (47.8%)	104 (46.4%)	212 (47.1%)
Participant’s perceived possible reason versus main reason for GP referral—n (%)			
Prediabetes or diabetes	55 (24.8) versus 24 (11)	57 (26.5) versus 25 (12)	112 (25.6) versus 49 (11)
Lower limb osteoarthritis	64 (28.3%) versus 27 (12)	45 (20.1) versus 26 (12)	109 (24.2) versus 53 (12)
Weight loss	182 (80.5) versus 114 (50)	182 (81.3) versus 113 (50)	364 (80.9) versus 227 (50)
Low mood	122 (54.0) versus 42 (18)	121 (54.0) versus 42 (19)	243 (54.0) versus 84 (19)
High blood pressure	79 (35.0) versus 19 (8)	68 (30.4) versus 18 (8)	147 (32.7) versus 37 (8)
Smoking status—n (%)			
Smoker	34 (15.0%)	32 (14.3%)	66 (14.7%)
Ex-smoker	90 (39.8%)	89 (39.7%)	179 (39.8%)
Never smoked	102 (45.1%)	103 (46.0%)	205 (45.6%)
IT literacy/confidence level—n (%)1			
Low	36 (16%)	35 (16%)	72 (16%)
High	190 (84%)	189 (84%)	379 (84%)
Site—n (%)			
Birmingham	78 (34%)	76 (34%)	154 (34%)
Glasgow	69 (31%)	72 (32%)	141 (31%)
Plymouth	79 (35%)	76 (34%)	155 (35%)
Weekly MVPA minutes (in ≥10 min bouts)—n, mean (SD)*Median (IQR)	201, 30.2 (105.8) 201, 0 (0, 23.3)	207, 31.8 (53.7) 207, 7.5 (0, 41.1)	408, 31.0 (83.4) 408, 0 (0–30.3)
Weekly MVPA minutes (no bouts)—n, mean (SD)* Median (IQR)	201, 319.5 (249.5) 264.6 (147.0–395.5)	207, 371.8 (251.3) 309.4 (196.7–490.7)	408, 346.0 (251.5) 288.4 (172.9–455.0)
n (%) achieving 150 min (in ≥10 min bouts)*	8/201 (4%)	9/207 (4%)	17/408 (4%)
n (%) achieving 150 min (no bouts)*	149/201 (74%)	178/207 (86%)	327/408 (80%)
Self-reported weekly MVPA minutes—n, mean (SD) Median (IQR)	220 213.5 (352.7) 65.0 (0–285.0)	220 204.0 (375.6) 47.5 (0–247.5)	440 208.8 (364.0) 40 (0–210)
N (%) achieving 150 weekly minutes of self-reported MVPA	83/220 (37%)	77/220 (35%)	160/440 (36%)
EQ-5D-5L (Devlin)—n, mean (SD)	216, 0.74 (0.24)	215 0.76 (0.23)	431, 0.75 (0.24)
HADS-D—n, mean (SD)	217, 7.6 (4.5)	214, 7.4 (4.7)	431, 7.5 (4.6)
HADS-A—n, mean (SD)	217, 8.7 (4.6)	214, 8.6 (5.1)	431, 8.6 (4.9)

On a 10 point Likert scale, scores of 1-5 indicated a low literacy level and scores of 6-10 a high literacy level.

*Accelerometer recorded.

BMI, body mass index; GCE, general certificate of education; GP PAQ, general practitioner physical activity questionnaire; MVPA, moderate-to-vigorous physical activity.

Approximately one-third of participants were recruited from each of the three sites. As an indication of the level of multimorbidity in the sample, 74.2% had two conditions, 30.7% had three conditions and 11.8% had four or more conditions.

There was a distinct difference at baseline, for the whole sample, between the mean (SD) weekly accelerometer MVPA minutes when recorded in ≥10 min bouts (31.0 (83.4)) and without bouts (346.0 (251.5)), and the proportion of the whole sample who achieved 150 min/week when recorded in ≥10 min bouts (4%) and without bouts (80%). These figures compared with self-reported data which showed a mean (SD) of 208.8 (364.0) minutes and 36% achieving 150 min/week.

### Intervention engagement

Among intervention participants, 36% did not register and log into the e-coachER website, and 36% progressed through to at least step 5. The proportion reaching each step is shown in online supplemental material appendix 8. The mean (SD) number of goal reviews was 2.5 (SD 4.5) with a range of 0–24. The 144 participants who registered, logged into e-coachER for a mean (SD) and median number of times of 14.1 (16.7) and 6, respectively, with a range from 1 to 101. Those who registered spent an estimated mean (SD) and median time engaging with the e-coachER web-based support of 48.4 (41.9) min and 36 min, respectively, with a range of 6–186 min.

10.1136/bjsports-2020-103121.supp8 Supplementary data

### Primary outcome

Table 3 shows the primary outcome summary scores at baseline, 4 and 12 months follow-up. Primary analysis showed a (non-significant) weak indicative effect in favour of the intervention at 12 months (mean difference 11.8 weekly minutes of MVPA, 95% CI: −2.1 to 26.0, p=0.10). Given the over dispersion and high frequency of zero values in the primary outcome, and the poor fit of the primary analysis model, alternative post-hoc regression models were explored. These included: log transformed mixed effects (with a constant added), mixed effect model with outliers removed, negative binomial and zero-inflated binomial models. These alternative models confirmed the interpretation of our primary analysis (see online supplemental material appendix 9 that also includes model fit graphs). The non-significant between group difference in primary outcome was consistent across the primary and post-hoc models.

10.1136/bjsports-2020-103121.supp9 Supplementary data

**Table 3:** Summary of primary outcome data at baseline and 4 and 12 months follow-up and analysis of between group differences in total weekly minutes (recorded in bouts and no bouts) at 12 months

	Baseline		4-month follow-up		12-month follow-up		Between group difference at 12 months* Mean (95% CI) P value
	Control (n=201) Mean (SD) Median (IQR)	Intervention (n=207) Mean (SD) Median (IQR)	Control (n=128) Mean (SD) Median (IQR)	Intervention (n=109) Mean (SD) Median (IQR)	Control (n=133) Mean (SD) Median (IQR)	Intervention (n=110) Mean (SD), Median (IQR)	
Total weekly minutes of MVPA in ≥10 min bouts	30.2 (105.8) 0 (0–23.3)	31.8 (53.7) 7.5 (0–41.1)	30.9 (64.5) 0 (0–45.9)	38.4 (74.5) 0 (0–49.4)	18.7 (37.4) 0 (0–19.8)	35.4 (78.3) 0 (0–40.4)	11.8 (−2.1 to 26.0), 0.10
Total weekly minutes of MVPA†	319.2 (249.2) 264.6 (147.0–395.5)	371.7 (251.3) 309.4 (196.7–490.7)	324.1 (264.6) 257.6 (151.2–375.2)	408.1 (251.3) 340.2 (231.7–521.5)	298.2 (210.7) 252.0 (144.2–420.0)	363.3 (256.2) 303.8 (186.9–469.0)	13.7 (−26.8 to 54.2), 0.51

Data from participants included as per primary analysis with 232 participants providing data at baseline and 12 months, and of these, from the 172 who provided data at 4 months.

*Adjusted for baseline MVPA, age, gender, site and minimisation variables.

†Unbouted minutes.

MVPA, moderate-to-vigorous physical activity.

CACE analyses for the primary outcome showed a mean difference of 22.9 weekly minutes of MVPA (95% CI: −3.4 to 47.8, p=0.09) in favour of the ERS group. There was no evidence of any interactions between stratification variables (site and reason for ERS referral), age and gender with the intervention effect for the primary outcome at 12 months. Sensitivity analysis showed that wear time (ie, days per week, hours per day, etc) did not influence the findings.

### Secondary outcome findings

Table 1 shows the summary descriptive secondary outcomes at baseline and 4 and 12 months follow-up. No significant differences in the primary analysis for any of the secondary outcomes at 12 months were seen except for intervention participants spending more time in daily diurnal inactivity (sedentary time) at 12 months. Secondary analysis models compared imputed secondary outcome data sets at 12 months and repeated measures analysis of primary and secondary outcomes at both 4 and 12 months were broadly consistent with the primary analyses results above.

There was no difference in ERS uptake, between the control group, 173/223 (78%) and intervention group, 167/223 (75%).

### Serious adverse events (SAEs)

In total, 42 SAEs were reported in 35 participants and were all deemed to be either ‘not related’ or ‘unlikely to be related’ to the trial. In the control group, there were 26 SAEs among 21 participants, and in the intervention group there were 16 SAEs among 14 participants. One SAE was reported as a life-threatening event (asthma attack), all other SAEs were hospitalisations. See online supplemental material appendix 10.

10.1136/bjsports-2020-103121.supp10 Supplementary data

## Discussion

### Summary of findings

To our knowledge this is the first randomised study to assess the effects of adding web-based behavioural support to usual ERS support on objectively assessed long-term minutes of MVPA among participants with chronic physical and mental health conditions. Augmenting usual ERS using web-based behavioural support (e-coachER) provided a (none statistically significant) weak indicative effect on objectively assessed minutes of MVPA (when recorded in ≥10 min bouts or not) at 12 months among inactive or moderately inactive patients. Various sensitivity analyses supported these findings. We also found no evidence of benefit in terms of ERS uptake and patient-reported outcomes. The extent of engagement with e-coachER was modest, but this factor did not influence the findings.

### Understanding the findings

Despite our best efforts, we were unable to get follow-up data from as many participants as we had planned and this may have reduced power to find a statistically significant effect. This has been a challenge for other ERS studies as well involving both device-based 25 and subjectively^26^ captured PA; for example, the latter study^26^ followed up only 55.6% of participants at 6 months. The e-coachER study was initially powered to detect differences in numbers achieving 150 min of MVPA based on our systematic review.^9^ Due to early recruitment issues, the sample size was recalculated as reported, with the primary outcome based on ≥10 min bouts. The scant available data from device-based assessed PA in comparable trials made the power calculation somewhat uncertain, and we also need to know more about what is a clinically significant change in device-based assessed MVPA to justify sample sizes.

Our prespecified analysis plan, involving a measure of MVPA in bouts of ≥10 min, meant that a larger proportion of the sample than expected recorded zero minutes. This required us to explore a number of analysis models for the primary analysis, none of which were ideal but did provide a consistent conclusion. Given that other studies have reported findings using a different accelerometer wear time (eg, Harris *et al* reported MVPA minutes from ‘at least 1 day’ to estimate weekly activity^22^ we also considered our data with a range of wear time criteria, and again the findings were consistent.

The primary focus was on differences in MVPA minutes at 12 months, but both groups showed an increase at 4 months. Our aim was to increase uptake and long-term change in MVPA, given concerns that ERS only foster short-term change,^9^ but providing the e-coachER intervention at the same time as what was somewhat effective ERS support may have limited the perceived need for e-coachER engagement. Also, digital support interventions are renowned for having relatively short-term engagement and effects, so it may be that greater digital support is needed after 4 months (the typical duration of ERS).

In line with guidelines for ERS research,^27^ we tried to make the intervention as accessible as possible to participants from a wide range of socioeconomic backgrounds, which we achieved to some extent, but with an increasing array of devices for self-monitoring and setting goals for PA, and it may be that many participants in both arms of our sample were independently accessing these, which thereby negated any benefits from the e-coachER intervention.

### Other considerations

We found a large discrepancy between the proportion of the sample who achieved 150 min of accelerometer recorded MVPA when assessed in ≥10 min bouts (4%) or not (80%), and by self-report (36%) at baseline, despite selecting sedentary or inactive participants for the trial. This finding also aligns with recently reported data from the USA, which identified a range of 3.4%–95.6% of people achieving 150 min of MVPA depending on how the accelerometer data were processed.^28^ This is important given the recent removal of the ‘≥10 min bouts’ in UK and international guidelines.^29 30^ It has been suggested that data collected using accelerometers is incompatible with guidelines of 150 min of MVPA per week and a value of about 1000 unbouted minutes of MVPA would need to be recommended for public health benefits.^31^ Our sample at baseline recorded only 346 min of unbouted weekly MVPA, which highlights the uniqueness of the study involving attempts to support change in such a low active sample, who potentially have the greatest to gain in terms of health from increases in MVPA.

A final consideration is that there was a small indication of imbalance in educational status between the groups at baseline, with a greater proportion of those with no qualification, and a slightly smaller proportion with higher qualifications, in the control group. However, given the relative small numbers of those in the respective categories for educational status, the fact that we had not specified inclusion of this variable in our statistical analysis plan for the primary analysis, and the absence of between group differences in IT confidence, we chose not to further explore any confounding effects of educational status.

### Further research

The focus on accumulating ≥10 min bouts for health benefit has now been dropped in global guidelines but in presenting both bouted and unbouted total MVPA the present study will provide valuable device-based information to inform future related research. Changes in international guidelines have removed the importance of completing PA in ≥10 min bouts, which in turn changes the basis for powering studies since a much greater proportion of the population are likely to meet the new unbouted target of 150 min of MVPA per week.

### Practical implications

There remain digital opportunities to provide support for patients to facilitate greater uptake of ERS and sustained change in PA for the management of chronic conditions. Bespoke software, drawing on some of the concepts and content in e-coachER could be used to ensure better links between the referee in primary care and patient. There can be confusion about what the ERS involves, compounded sometimes by delayed starting. Beyond the formal ERS, digital systems could be implemented to maintain long-term MVPA.

## Conclusion

Augmenting ERS referrals with web-based behavioural support had only a weak, non-significant indicative effect on accelerometer recorded MVPA at 12 months, and no effect on ERS engagement. Overall, there was only modest engagement in the e-coachER web-based support, but degree of engagement did not influence the overall findings.

**What are the key findings?**

- With lower than desired follow-up rates, we found no significant effect of augmenting usual primary care exercise referral schemes with the e-coachER intervention on 12 month objectively assessed physical activity (PA), among low active participants with chronic conditions.
- Engagement in the web-based support was modest despite being based on contemporary behaviour change theories, other effective interventions and good public and patient and stakeholder involvement in the development.

**How might it impact on clinical practice in the future?**

- The study was conducted pre COVID 19 and the need to find effective digital support for patients to facilitate greater uptake of exercise referral scheme (ERS) and sustained change in PA for the management of chronic conditions has only increased.
- Local digital solutions could be developed in primary care to better manage and monitor the progress of patients in an ERS to inform further conversations about self-management of chronic conditions.

10.1136/bjsports-2020-103121.supp11 Supplementary data
