# Supplementary material for: Randomised controlled trial of an augmented exercise referral scheme using web-based behavioural support for inactive adults with chronic health conditions: the e-coachER trial
Source: Br J Sports Med. 2020 Nov 27;55(8):444–50. doi: 10.1136/bjsports-2020-103121 (PMC8020080; doi:10.1136/bjsports-2020-103121)
Supplement: Supplementary data [file bjsports-2020-103121supp001.pdf]

Supplementary material - Appendix 1: Full list of inclusion/exclusion criteria

Inclusion criteria:

- Aged between 16 and 74 years inclusive
- Had one of more of the following conditions:
    - obesity i.e. a body mass index of 30–40 kg/m^2
    - hypertension
    - pre-diabetes
    - type 2 diabetes
    - lower limb osteoarthritis
    - current or recent history of treatment for depression
- Categorised as ‘inactive’ (i.e. 0 hours per week of physical exercise and in a sedentary occupation) or ‘moderately inactive’ (i.e. some activity but < 1 hour per week and in a sedentary occupation or 0 hours per week of physical exercise and in a standing occupation) according to the General Practice Physical Activity Questionnaire (GPPAQ).[1]

Patients were excluded for the following reasons:

- Did not meet the eligibility criteria for their local ERS
- Had an unstable, severe and enduring mental health problem
- Were being treated for an alcohol or drug addiction that may have limited their involvement with the study
- Were unable to use written materials in English, unless there was a designated family member or friend to act as translator.

1. Ahmad S, Harris T, Limb E, et al. Evaluation of reliability and validity of the General Practice Physical Activity Questionnaire (GPPAQ) in 60-74 year old primary care patients. *BMC Fam Pract* 2015;16:113. doi: 10.1186/s12875-015-0324-8 [published Online First: 2015/09/04]
