# Supplementary material for: Randomised controlled trial of an augmented exercise referral scheme using web-based behavioural support for inactive adults with chronic health conditions: the e-coachER trial
Source: Br J Sports Med. 2020 Nov 27;55(8):444–50. doi: 10.1136/bjsports-2020-103121 (PMC8020080; doi:10.1136/bjsports-2020-103121)
Supplement: Supplementary data [file bjsports-2020-103121supp002.pdf]

Supplementary material - Appendix 2: Routes of approaching potential participants

Patients were identified as potentially eligible for the trial in a number of different ways:

- By health-care professionals in primary care at the point of being actively referred to an ERS or having been opportunistically found to be eligible for an ERS at a consultation with the primary care practitioner.
- Via a search of patient databases at the participating GP practices (conducted by the local NIHR Primary Care Research Network team).
- Via patient self-referral to the GP arising from community-based publicity for the trial.
- By the ERS programme administrator on receipt of an ERS referral form from a GP practice.
- By exercise advisors at the ERS service at enrolment on the ERS. With the patient's consent, the exercise advisor provided the local researcher with the patient's contact details for the purposes of the trial.
