# Supplementary figures and images for: Randomised controlled trial of an augmented exercise referral scheme using web-based behavioural support for inactive adults with chronic health conditions: the e-coachER trial
Source: Br J Sports Med. 2020 Nov 27;55(8):444–50. doi: 10.1136/bjsports-2020-103121 (PMC8020080; doi:10.1136/bjsports-2020-103121)

## Supplementary material – Appendix 3: Participant pathway

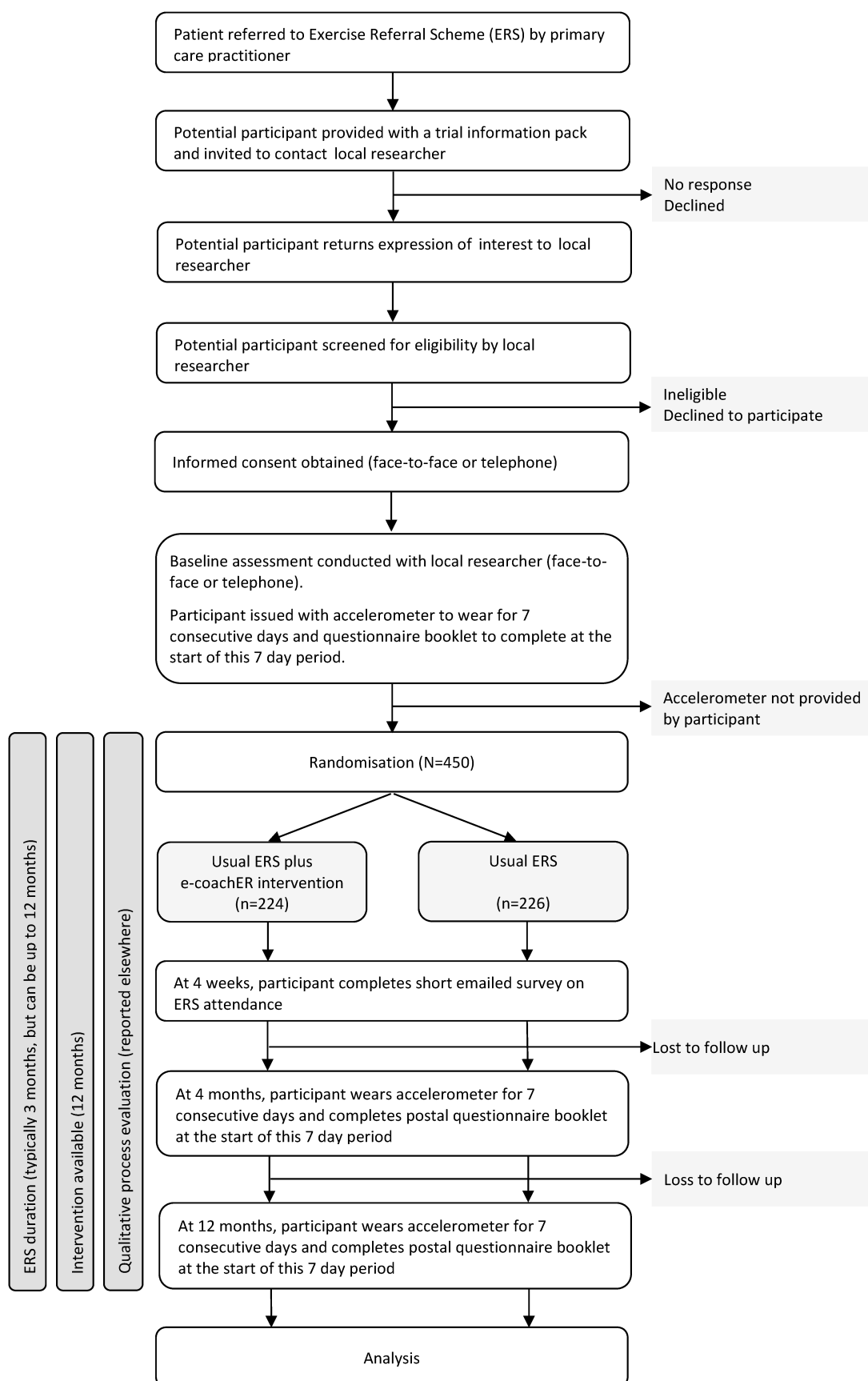

Supplement: Supplementary data [file bjsports-2020-103121supp003.pdf]
