# Supplementary material for: Randomised controlled trial of an augmented exercise referral scheme using web-based behavioural support for inactive adults with chronic health conditions: the e-coachER trial
Source: Br J Sports Med. 2020 Nov 27;55(8):444–50. doi: 10.1136/bjsports-2020-103121 (PMC8020080; doi:10.1136/bjsports-2020-103121)
Supplement: Supplementary data [file bjsports-2020-103121supp004.pdf]

Supplementary material - Appendix 4: Data reduction decisions for processing accelerometers for the e-coachER study

Epochs:	5
Valid day criteria:	16 hours
Number of valid days:	4 including 1 weekend day
Cut points	Converted from SVMgs to Milli-g values[1]
Daily Analysis period	Midnight to Midnight

1. Esliger DW, Rowlands AV, Hurst TL, et al. Validation of the GENEA Accelerometer. *Medicine and science in sports and exercise* 2011;43(6):1085-93. doi: 10.1249/MSS.0b013e31820513be [published Online First: 2010/11/23]
