# Supplementary material for: Randomised controlled trial of an augmented exercise referral scheme using web-based behavioural support for inactive adults with chronic health conditions: the e-coachER trial
Source: Br J Sports Med. 2020 Nov 27;55(8):444–50. doi: 10.1136/bjsports-2020-103121 (PMC8020080; doi:10.1136/bjsports-2020-103121)
Supplement: Supplementary data [file bjsports-2020-103121supp005.pdf]

Supplementary material – Appendix 5: Accelerometer recorded and self-reported secondary outcome measures at 4 and 12 months

- Total weekly minutes of MVPA in ≥10 minute bouts, measured objectively by accelerometer, over one week at four months.
- Achievement of at least 150 minutes of MVPA, measured objectively by accelerometer, over one week at four and twelve months.
- Self-reported achievement of at least 150 minutes of MVPA over one week using the 7 day recall of PA[1] (7-day PAR) at four and twelve months.
- Self-reported weekly minutes of MVPA at four and twelve months.
- Average daily hours of sedentary behaviour measured objectively by accelerometer over one week at four and twelve months.
- Self-reported average daily hours of sleep over one week at four and twelve months.
- Self-reported health-related quality of life, assessed by the EQ-5D-5L[2] at four and twelve months.
- Self-reported symptoms of anxiety and depression, assessed by the Hospital Anxiety and Depression Scale[3] at four and twelve months.

1. Blair SN, Haskell WL, Ho P, et al. Assessment of habitual physical activity by a seven-day recall in a community survey and controlled experiments. *Am J Epidemiol* 1985;122(5):794-804. [published Online First: 1985/11/01]
2. Herdman M, Gudex C, Lloyd A, et al. Development and preliminary testing of the new five-level version of EQ-5D (EQ-5D-5L). *Qual Life Res* 2011;20(10):1727-36. doi: 10.1007/s11136-011-9903-x [published Online First: 2011/04/12]
3. Zigmond AS, Snaith RP. The hospital anxiety and depression scale. *Acta Psychiatr Scand* 1983;67(6):361-70. [published Online First: 1983/06/01]
