# Supplementary material for: Randomised controlled trial of an augmented exercise referral scheme using web-based behavioural support for inactive adults with chronic health conditions: the e-coachER trial
Source: Br J Sports Med. 2020 Nov 27;55(8):444–50. doi: 10.1136/bjsports-2020-103121 (PMC8020080; doi:10.1136/bjsports-2020-103121)
Supplement: Supplementary data [file bjsports-2020-103121supp006.pdf]

Supplementary material - Appendix 6: Additional wear time criteria used in the sensitivity analysis

Sensitivity analysis were conducted for the following four additional wear time criteria:

1) ≥16 hours over any four days (irrespective of week/weekend);

2) ≥10 hours for 4 days (including at least one weekend day);

3) ≥10 hours over any four days (irrespective of week/weekend);

4) A minimum wear criteria of 1 day for 10 hours but with individuals weighted by the number of valid days with a minimum of 10 hours wear.
