# Supplementary material for: Randomised controlled trial of an augmented exercise referral scheme using web-based behavioural support for inactive adults with chronic health conditions: the e-coachER trial
Source: Br J Sports Med. 2020 Nov 27;55(8):444–50. doi: 10.1136/bjsports-2020-103121 (PMC8020080; doi:10.1136/bjsports-2020-103121)
Supplement: Supplementary data [file bjsports-2020-103121supp007.pdf]

Supplementary material - Appendix 7: Table showing reasons for ineligibility at each stage of recruitment

After expression of interest received (n=831)	**Ineligible (n=11)**<br>No email/internet n=4<br>Other reasons n=3<br>Age outside range n=1<br>Doesn't meet ERS criteria n=1<br>No clinical condition of interest n=1<br>Too active (physically active occupation) n=1
After person contacted (n = 755)	**Ineligible (n=23)**<br>No email/internet n=9<br>Other/combined reasons n=6<br>Doesn't meet ERS criteria n=4<br>No translator n=2<br>Age outside range n=1<br>Too active (physically active occupation) n=1
After assessing for eligibility (n = 691)	**Ineligible at screening (n=201)**<br>BMI outside range n=104<br>Too active on GPPAQ n=46<br>No clinical condition of interest n=26<br>Age outside range n=10<br>No email/internet n=6<br>Substance abuse problem n=3<br>Other reasons n=3<br>Doesn't meet ERS criteria n=2<br>BP outside range n=1
