# Supplementary material for: Randomised controlled trial of an augmented exercise referral scheme using web-based behavioural support for inactive adults with chronic health conditions: the e-coachER trial
Source: Br J Sports Med. 2020 Nov 27;55(8):444–50. doi: 10.1136/bjsports-2020-103121 (PMC8020080; doi:10.1136/bjsports-2020-103121)
Supplement: Supplementary data [file bjsports-2020-103121supp008.pdf]

Supplementary material – Appendix 8: Table showing level of engagement at each intervention step

**Stage started**	**Summary of content**	**Number (% of 224 in intervention arm)**
Did NOT register		81 (36%)
Step 1	Quiz on benefits of PA	144 (64%)
Step 2	Support to get active	133 (59%)
Step 3	Encourage self-monitoring of steps	107 (48%)
Step 4	Setting SMART step-count goals for next week	99 (44%)
Step 5	Setting SMART goals for any PA for next week	96 (43%)
Goal review	Review goal and personalised feedback	81 (36%)
