# Supplementary material for: Randomised controlled trial of an augmented exercise referral scheme using web-based behavioural support for inactive adults with chronic health conditions: the e-coachER trial
Source: Br J Sports Med. 2020 Nov 27;55(8):444–50. doi: 10.1136/bjsports-2020-103121 (PMC8020080; doi:10.1136/bjsports-2020-103121)

Appendix 9 – Model fit graphs and statistics for primary analysis and post-hoc regression for the primary outcome

	**Between group difference**<br>**Mean (95% CI) P-value**
Primary analysis model	Mean difference: 11.8 (-2.1 to 26.0), 0.10
Post-hoc model 1: outliers [MVPA > 200] dropped	Mean difference: 2.5 (-5.8 to 10.7), 0.55
Post-hoc model 2: log outcome + constant of 5	Mean difference: 1.2 (0.8 to 1.5), 0.27
Post hoc model 3: negative binomial model	Rate ratio: 1.90 (0.90 to 4.00), 0.09
Post-hoc model 4: zero-inflated negative binomial model	Rate ratio: 1.59 (1.13 to 2.25), 0.01

Primary analysis

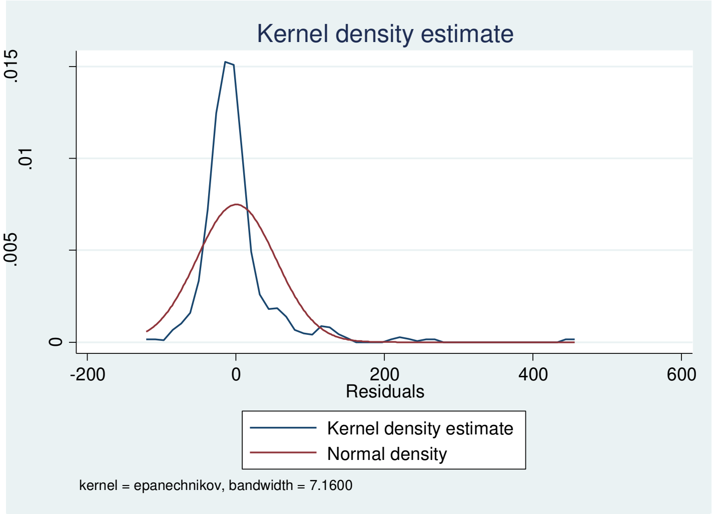

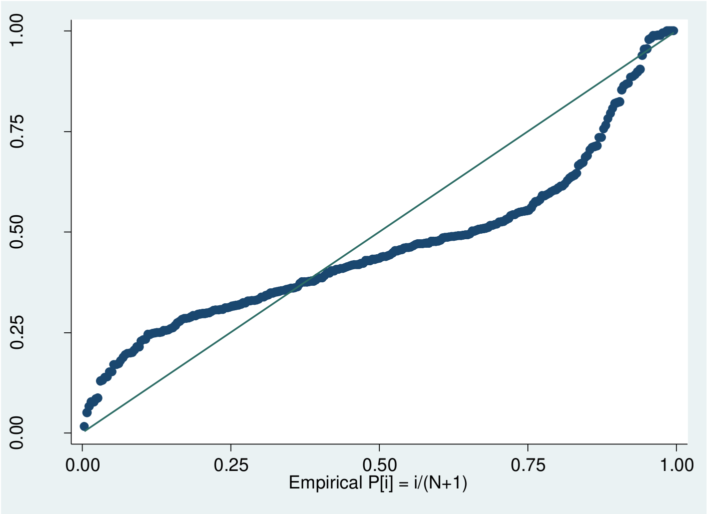

Outliers [>200] dropped model

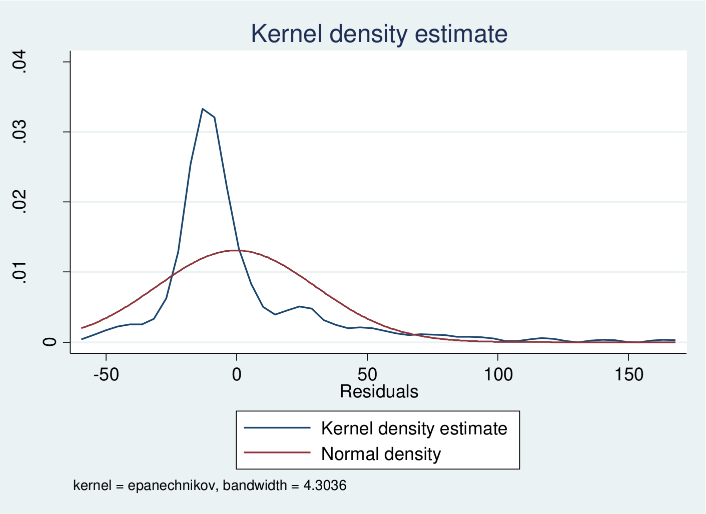

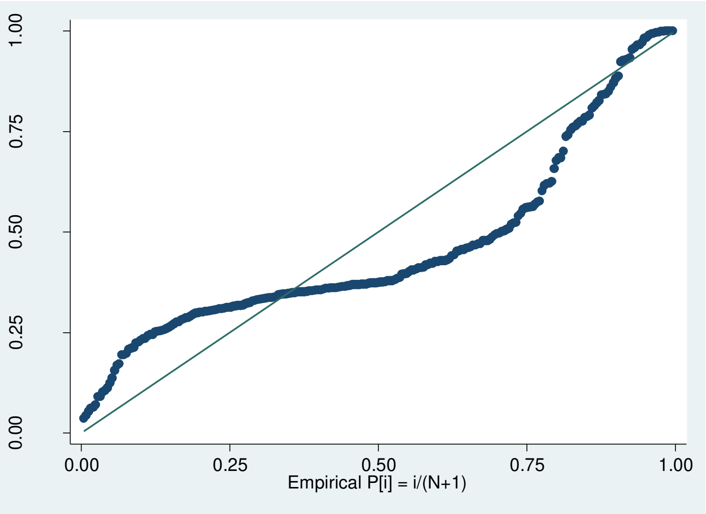

Log model with constant of 5 added

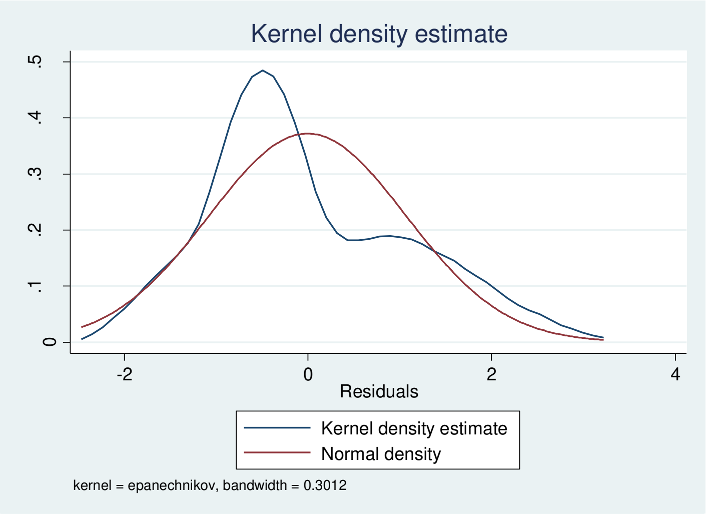

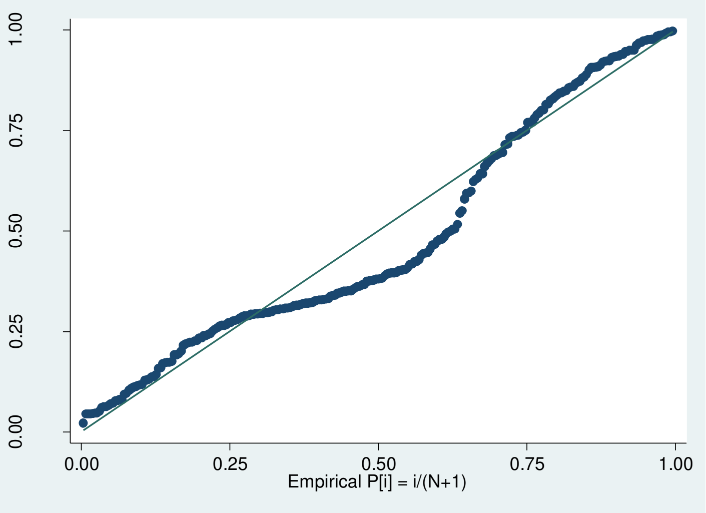

Supplement: Supplementary data [file bjsports-2020-103121supp009.pdf]
