# Supplementary material for: Randomised controlled trial of an augmented exercise referral scheme using web-based behavioural support for inactive adults with chronic health conditions: the e-coachER trial
Source: Br J Sports Med. 2020 Nov 27;55(8):444–50. doi: 10.1136/bjsports-2020-103121 (PMC8020080; doi:10.1136/bjsports-2020-103121)
Supplement: Supplementary data [file bjsports-2020-103121supp010.pdf]

Supplementary material – Appendix 10: Tables showing Serious Adverse Events

Serious Adverse Events (SAE) reported in the control group

Participant ID (not study number)	MedDRA organ system	Summary description of event
1	Neoplasms (2)	Diagnosed with chronic myeloid leukaemia.
2	Neoplasms (2)	Prolonged hospitalisation due to recurrence of breast cancer.
3	Psychiatric (7)	Inpatient stay on mental health ward.
2	Psychiatric (7)	Hospitalised for depression.
4	Nervous system (8)	Morton's Neuroma.
5	Respiratory (13)	Treated in hospital for fluid on the lungs.
6	Gastrointestinal (14)	Varices of gastrointestinal tract. Prolonged inpatient stay due to major organ system involvement.
7	Musculoskeletal (17)	Admitted to hospital because unable to walk
8	Pregnancy (19)	Childbirth and post-natal inpatient stay.
6	Investigations (23)	Admitted to hospital with symptoms of meningitis.
9	Investigations (23)	Collapse. No diagnosis made.
10	Surgical/medical (25)	Planned admission for femorodistal bypass (peripheral vascular disease), subsequent infection/abscess behind knee.
1	Surgical/medical (25)	Hospitalised for treatment of boils in groin.
11	Surgical/medical (25)	Planned hospital admission for bunion removal.
9	Surgical/medical (25)	Planned hospital admission for bunion removal
12	Surgical/medical (25)	Hospital admission for treatment for diverticular bleeding.
13	Surgical/medical (25)	Injury to foot led to planned admission for partial amputation of left great toe.
14	Surgical/medical (25)	Planned hospital admission for right hip replacement.
15	Surgical/medical (25)	Planned hospitalisation for total hip replacement.
16	Surgical/medical (25)	Planned hospital admission for total knee replacement.
17	Surgical/medical (25)	Pre-planned hospitalisation for knee surgery due to osteoarthritis.
18	Surgical/medical (25)	Planned admission for total right knee replacement.
18	Surgical/medical (25)	Planned admission following infected right knee joint - continuing physio and using crutches.
19	Surgical/medical (25)	Hospitalisation for emergency operation on knee following a number of falls and pre-existing weakness in knee.
20	Surgical/medical (25)	Hospital admission for surgical repair of bulging disc in lower back.
21	Surgical/medical (25)	Hospitalised due to complications from type-2 diabetes and heart failure.

Serious Adverse Events (SAE) reported in the intervention group

Participant ID (not study number)	MedDRA organ system	Summary Description of Event
22	Cardiac (11)	Admitted to hospital with abnormal ECG. Diagnosis: Paroxysmal Atrial Fibrillation
23	Cardiac (11)	Hospitalised due to heart attack
24	Vascular (12)	Hospitalised due to minor stroke.
25	Respiratory (13)	Asthma attack.
26	Investigations (23)	Hospital admission ?meningitis. No formal diagnosis made. Symptoms attributed to adverse effects of prescription medication.
27	Investigations (23)	Fall resulting in fracture of left radius. Admitted for investigations of reasons for the fall.
28	Surgical/medical (25)	Admitted to hospital following fall with fracture to right ankle and trauma to right knee
29	Surgical/medical (25)	Preplanned hospital admission for abdominal surgery
30	Surgical/medical (25)	Planned hospitalisation for operation on right ankle
31	Surgical/medical (25)	Admitted to hospital for 1 day (day case) due to osteoarthritis
32	Surgical/medical (25)	Planned hospital admission for tendon surgery on hand related to rheumatoid arthritis
33	Surgical/medical (25)	Hospital admission for treatment of rheumatoid arthritis flare-up.
34	Surgical/medical (25)	Inpatient stay for removal of Bartholin's cyst
29	Surgical/medical (25)	Planned hospital admission for knee replacement.
28	Surgical/medical (25)	Planned admission for partial right knee replacement
35	Surgical/medical (25)	Hospitalised for surgery on both knees, as treatment for long-standing osteoarthritis.
