# Supplementary material for: Randomised controlled trial of an augmented exercise referral scheme using web-based behavioural support for inactive adults with chronic health conditions: the e-coachER trial
Source: Br J Sports Med. 2020 Nov 27;55(8):444–50. doi: 10.1136/bjsports-2020-103121 (PMC8020080; doi:10.1136/bjsports-2020-103121)
Supplement: Supplementary data [file bjsports-2020-103121supp011.pdf]

Open access Protocol

BMJ Open

Multicentred randomised controlled trial of an augmented exercise referral scheme using web-based behavioural support in individuals with metabolic, musculoskeletal and mental health conditions: protocol for the e-coachER trial

Wendy Ingram,[1] Douglas Webb,[1] Rod S Taylor,[2] Nana Anokye,[3] Lucy Yardley,[4,5] Kate Jolly,[6] Nanette Mutrie,[7] John L Campbell,[2] Sarah Gerard Dean,[2] Colin Greaves,[6] Mary Steele,[4] Jeffrey D Lambert,[2] Chloe McAdam,[7] Ben Jane,[8] Jennie King,[9] Ray B Jones,[1] Paul Little,[4] Anthony Woolf,[10] Jo Erwin,[10] Nigel Charles,[2] Rohini H Terry,[2] Adrian H Taylor[1]

To cite: Ingram W, Webb D, Taylor RS, *et al.* Multicentred randomised controlled trial of an augmented exercise referral scheme using web-based behavioural support in individuals with metabolic, musculoskeletal and mental health conditions: protocol for the e-coachER trial. *BMJ Open* 2018;**8**:e022382. doi:10.1136/bmjopen-2018-022382

► Prepublication history for this paper is available online. To view these files, please visit the journal online (http://dx.doi.org/10.1136/bmjopen-2018-022382).

Received 14 February 2018
Revised 16 May 2018
Accepted 21 May 2018

Check for updates

© Author(s) (or their employer(s)) 2018. Re-use permitted under CC BY. Published by BMJ.

For numbered affiliations see end of article.

Correspondence to
Dr Wendy Ingram;
wendy.ingram@plymouth.ac.uk

ABSTRACT

Introduction Physical activity is recommended for improving health among people with common chronic conditions such as obesity, diabetes, hypertension, osteoarthritis and low mood. One approach to promote physical activity is via primary care exercise referral schemes (ERS). However, there is limited support for the effectiveness of ERS for increasing long-term physical activity and additional interventions are needed to help patients overcome barriers to ERS uptake and adherence. This study aims to determine whether augmenting usual ERS with web-based behavioural support, based on the LifeGuide platform, will increase long-term physical activity for patients with chronic physical and mental health conditions, and is cost-effective.

Methods and analysis A multicentre parallel two-group randomised controlled trial with 1:1 individual allocation to usual ERS alone (control) or usual ERS plus web-based behavioural support (intervention) with parallel economic and mixed methods process evaluations. Participants are low active adults with obesity, diabetes, hypertension, osteoarthritis or a history of depression, referred to an ERS from primary care in the UK. The primary outcome measure is the number of minutes of moderate-to-vigorous physical activity (MVPA) in ≥10 min bouts measured by accelerometer over 1 week at 12 months. We plan to recruit 413 participants, with 88% power at a two-sided alpha of 5%, assuming 20% attrition, to demonstrate a between-group difference of 36–39 min of MVPA per week at 12 months. An improvement of this magnitude represents an important change in physical activity, particularly for inactive participants with chronic conditions.

Ethics and dissemination Approved by North West Preston NHS Research Ethics Committee (15/NW/0347). Dissemination will include publication of findings for the stated outcomes, parallel process evaluation and economic evaluation in peer-reviewed journals. Results will be disseminated to ERS services, primary healthcare providers and trial participants.

Trial registration number ISRCTN15644451; Pre-results.

Strengths and limitations of this study

- ► This is the first study to determine whether adding web-based interventions to primary care exercise referral schemes increases objectively assessed physical activity more than usual exercise referral schemes, after 1 year.
- ► The study includes inactive adults with one or more common chronic conditions.
- ► No physical health measures (except self-reported weight) were assessed in the study.
- ► It is expected that participants will have multiple chronic conditions, meaning the study may not be able to determine intervention effects on physical activity for each condition.
- ► Participants in the intervention arm will be invited to take part in in-depth qualitative interviews which may act as a cointervention.

INTRODUCTION

Physical inactivity was found to cost the National Health Service (NHS) £455 million in 2013–2014 according to data collected by Clinical Commissioning Groups in the UK.[1]

BMJ Open: first published as 10.1136/bmjopen-2018-022382 on 21 September 2018. Downloaded from http://bmjopen.bmj.com/ on December 23, 2019 at University of Plymouth - Library A. Henderson-Lib & Media Ser. Protected by copyright.

Evidence-based guidelines recommend both aerobic and strength training for improving health markers and quality of life among those with common chronic metabolic conditions[2–5] and musculoskeletal conditions,[6] and mostly aerobic exercise for preventing and reducing depression.[7] Public health guidelines of 150 min of moderate-to-vigorous physical activity (MVPA) per week are widely accepted but even small increases in physical activity and reduced sedentary time among the least active are likely to accrue health benefits.[8 9]

Patients with obesity, hypertension, type 2 diabetes, osteoarthritis and depression are less physically active than the general population,[2] and need greater support to overcome real and perceived barriers to increase physical activity. Increases in physical activity among the least active have the potential to provide the largest impact on health but any benefits dissipate without maintained levels of activity.[10] A variety of initiatives have been explored to promote physical activity within primary care, including referring patients to 'exercise on prescription', that is, an exercise referral scheme (ERS). In the UK, ERS have been common for promoting physical activity, with an estimated 600 schemes involving up to 100 000 patients per year.[11]

Evidence from a meta-analysis of eight randomised trials involving 5190 participants eligible for ERS[12] indicated a small increase in the proportion of participants who achieved 90–150 min of physical activity of at least moderate intensity per week, compared with no exercise control at 6–12 months follow-up among at-risk individuals. But uncertainty remains regarding the effects for patients with specific medical conditions since no study assessed long-term physical activity objectively, and many of the eight studies reviewed had relatively small sample sizes.

A systematic review[13] reported an average ERS uptake (attendance at the first ERS session) that ranged from 66% in observational studies to 81% in randomised controlled trials, and average levels of adherence from 49% in observational studies to 43% in randomised controlled trials. Predictors of uptake and adherence have rarely been explored but it has been reported that while women were more likely to begin an ERS, they were less likely to adhere to it than men; also, older people were more likely to begin and adhere to an ERS.[13] ERS may help patients become familiar with concepts such as exercise type, intensity, frequency and duration of exercise, matched to their medical condition, and target key processes of behaviour change. However, the following features of an ERS may reduce uptake and adherence: inconvenience, cost, limited sustainable physical activity support (eg, for 10 weeks) and low appeal for structured exercise and/or the medical model, that is, 'exercise on prescription', which may do little to provide autonomous support nor empower patients to develop self-determined behaviour to manage chronic medical conditions.[11 14] It therefore appears that additional support may be needed which is accessible, low cost, can be tailored to support a wide range of individual needs and empowers patients to develop and use self-regulatory skills (eg, self-monitoring, goal setting) to self-manage their chronic conditions. A wide variety of online and mobile technologies have been developed and used to support changes in and maintenance of physical activity.

There is considerable evidence on the effects of technology-based interventions for promotion of physical activity.[15 16] These include studies with a wide range of interventions (from quite simple self-monitoring to interventions with complex multiple behaviour change components), targeted at different clinical groups with different baseline levels of physical activity, with various physical activity outcomes reported (very few using objective measures), and with mostly short-term follow-ups. Also, some comparisons are between intervention versus no intervention and others versus human contact, although none reports on the effects of adding web-based support to ERS. The impact for web-based and technology interventions on increasing physical activity is small to moderate (an effect size ≤0.4). However, there is evidence that more rigorous studies, interventions with more behaviour change components and ones targeted at less active populations are more effective.[15 16] A systematic review[17] has highlighted the importance of maximising sustained engagement in web-based interventions for enhancing change in the target behaviour. A recent study[18] confirmed that self-monitoring of physical activity and tailored feedback were important to increase engagement, and periodic communications helped to maintain participant engagement.

The LifeGuide platform (www.LifeGuideonline.org/) has been extensively used to develop and evaluate acceptability and impact of online behaviour change and self-management interventions with a variety of clinical groups, including in primary care.[19–21] For example, adding online LifeGuide support to face-to-face support showed a greater lasting reduction in obesity than face-to-face dietetic advice alone.[22] The LifeGuide platform provides a researcher-led tool to develop interventions drawn from theory and evidence of effective techniques[23 24] and provides the opportunity to understand engagement and utility of different behaviour change components.

Following iterative development work and user group testing and involvement, drawing on some online modules used in other LifeGuide interventions,[19] we developed a bespoke intervention, called 'e-coachER' to support patients with chronic physical and mental health conditions who have been referred from primary care to an ERS to receive face-to-face support. Should the approach prove to be effective, there is considerable potential for the intervention to be scaled up for patients with obesity, hypertension, type 2 diabetes, osteoarthritis and risk of depression at probable low cost[25 26] and also extend it for patients with other chronic medical conditions (eg, low back pain, heart disease, cancer).

BMJ Open: first published as 10.1136/bmjopen-2018-022382 on 21 September 2018. Downloaded from http://bmjopen.bmj.com/ on December 23, 2019 at University of Plymouth - Library A. Henderson-Lib & Media Ser. Protected by copyright.

AIM AND OBJECTIVES

The overarching aim is to determine if e-coachER online support combined with usual ERS provides an effective and cost-effective approach to supporting increases in physical activity in people referred to ERS with a range of chronic conditions.

The specific objectives are as follows:

- To determine whether in the intervention arm compared with the control arm, there is an increase in the total weekly minutes of MVPA at 12 months postrandomisation.
- To determine whether in the intervention arm compared with the control arm there is an increase in the proportion of participants who:
  - take up the opportunity to attend an initial consultation with an exercise practitioner;
  - maintain objectively assessed physical activity from 4 to 12 months postrandomisation;
  - maintain self-reported physical activity from 4 to 12 months postrandomisation;
  - have improved health-related quality of life at 4 and 12 months postrandomisation.
- To quantify the additional costs of delivering the intervention and determine the differences in health utilisation and costs between the intervention and control arms at 12 months postrandomisation.
- To assess the cost-effectiveness of the intervention compared with control at 12 months postrandomisation (incremental cost per quality-adjusted life-year (QALY)) and over the lifetime perspective (incremental cost per QALY).
- To quantitatively and qualitatively explore whether the impact of the intervention is moderated by medical condition, age, gender and socioeconomic status, IT literacy or ERS characteristics.
- To quantitatively and qualitatively explore the mechanisms through which the intervention may impact on the outcomes, through rigorous mixed methods process evaluation and mediation analyses (if appropriate).

METHODS AND ANALYSIS

This protocol is reported in accordance with the Standard Protocol Items: Recommendations for Interventional Trials guidance[27] (http://www.spirit-statement.org/spirit-statement/) for protocols of clinical trials and TIDieR guidelines[28] (http://www.equator-network.org/reporting-guidelines/tidier/) for intervention description.

Study design and setting

This is a multicentre parallel two-group randomised controlled trial with participant allocation to usual ERS alone (control) or usual ERS plus web-based behavioural support (intervention) with parallel economic and mixed methods process evaluations. The trial design is summarised in figure 1.

Recruitment to the trial will take place over a 21-month period (July 2015 to March 2017) in three areas in the UK, that is, Greater Glasgow, West Midlands and South West England (including Plymouth, Cornwall and Mid Devon). Only the latter includes some participants in more rural locations.

Study population

The study population will include patients registered with a general practitioner (GP) surgery and who have been or are about to be referred to a local ERS for a programme of support to increase physical activity. Participants will be aged 16–74 years and have one of more of the following: obesity (body mass index (BMI), 30–40), a diagnosis of hypertension, prediabetes, type 2 diabetes, lower limb osteoarthritis or having a history of treatment for depression. Participants must also be categorised as 'inactive' or 'moderately inactive' based on the GP Physical Activity Questionnaire,[29] be contactable via email, and have some experience of using the internet. Patients are excluded if they meet any of the following criteria: have an unstable, severe and enduring mental health problem or are being treated for an alcohol or drug addiction that may limit their involvement with the study, do not meet the eligibility criteria for their local ERS or are unable to use written materials in English unless a designated family member or friend can act as translator.

Study procedures

Patient identification, approach and consent

Patients will be identified as potentially eligible for the trial (i) by healthcare professionals in primary care at the point of being actively referred to an ERS or having been opportunistically found to be eligible for an ERS at a consultation with the primary care practitioner, (ii) via a search of patient databases at the participating GP practices (conducted by the local Primary Care Research Network team), (iii) via patient self-referral to the GP arising from community-based publicity for the trial, (iv) by the ERS programme administrator on receipt of an ERS referral form from a GP practice or (v) by exercise advisors at the ERS service at enrolment on the ERS (with the patient's consent, the exercise advisor will provide the local researcher with the patient's contact details for the purposes of the trial).

Potentially eligible patients will be approached by the primary care practitioner or the local researcher, depending on how the patient was identified, or patients may self-refer to the local researcher in response to publicity campaigns. These various means of identification and approach are designed to accommodate the variation in usual care referral pathways to ERS across the participating sites and individual GP practices.

Amenable patients will be offered a study-specific Participant Information Sheet, either by post, via email or by hand (the route used will largely depend on the preference of the participating GP practice or ERS service). Interested patients will be asked to

BMJ Open: first published as 10.1136/bmjopen-2018-022382 on 21 September 2018. Downloaded from http://bmjopen.bmj.com/ on December 23, 2019 at University of Plymouth - Library A. Henderson-Lib & Media Ser. Protected by copyright.

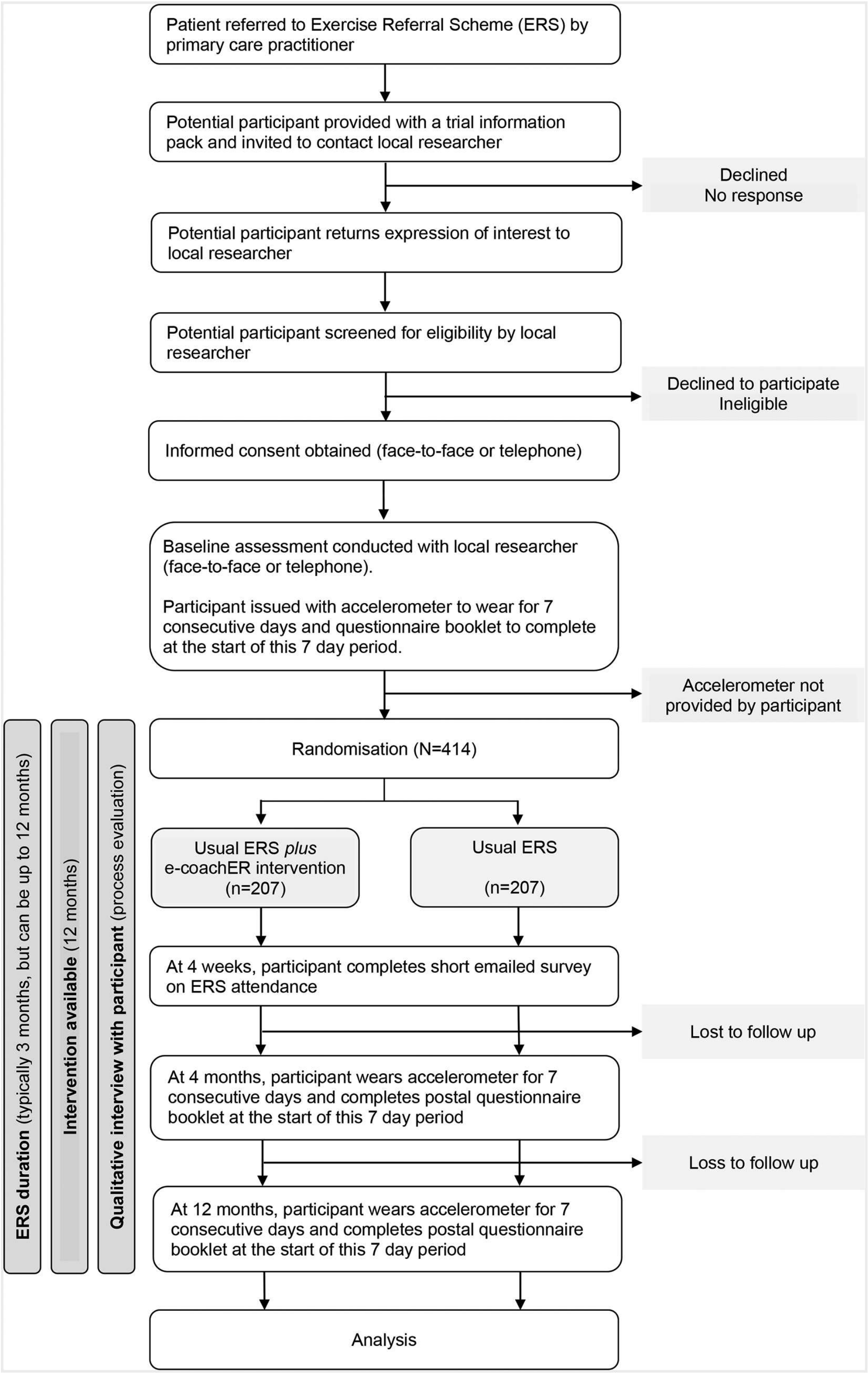

Figure 1 Trial design/participant pathway.

communicate their expression of interest to the local researcher via a prepaid reply slip, by telephone or by email. On receipt of an expression of interest, the local researcher will contact the potential participant by telephone to discuss the trial, confirm eligibility and take informed consent.

BMJ Open: first published as 10.1136/bmjopen-2018-022382 on 21 September 2018. Downloaded from http://bmjopen.bmj.com/ on December 23, 2019 at University of Plymouth - Library A. Henderson-Lib & Media Ser. Protected by copyright.

Table 1 Schedule of baseline and follow-up measures

Measure	Baseline	Randomisation	4 weeks	4 months	12 months
Demographics	X				
Objectively measured physical activity (eg, minutes of MVPA in ≥10 min bouts, recorded by accelerometer)	X			X	X
Engagement with the ERS (uptake at 4 weeks, plus subsequent attendance at ERS, eg, number of sessions attended)			X	X	
Engagement with e-coachER (captured from the LifeGuide platform)		X		X	X
Self-reported:<br>► MVPA (7-day recall of physical activity)<br>► Health and social care resource use<br>► Quality of life measures: 5-level Euroqol-5D (EQ-5D-5L), SF-12v2<br>► Hospital Anxiety and Depression Scale	X			X	X
Process evaluation outcomes (eg, self-reported confidence to be physically active; perceived frequency and availability of support; perceived autonomy over choices; involvement in self-monitoring and planning physical activity)	X			X	X
Qualitative interviews as part of the process evaluation focusing on participants' experiences with the ERS and the intervention (optional for participants)		———————————————	———————————	X————————	———————————

ERS, exercise referral scheme; MVPA, moderate- to- vigorous physical activity.

Baseline assessment

Consented participants will attend a baseline assessment with the local researcher. This assessment will be conducted over the telephone, or in person at the GP practice or at the centre delivering the ERS or another convenient community location. Demographic data will be collected. The participant will be issued with a wrist-worn waterproof accelerometer (GENEActiv Original accelerometer http://www.geneactiv.org/) to wear constantly for one whole week (day and night), and a self-report questionnaire booklet to complete at the beginning of the week-long period. The accelerometer will be worn on the wrist of the non-dominant hand (ie, the hand not favoured for writing). After 1 week's wear, participants will post the accelerometer and completed questionnaire to the Peninsula Clinical Trials Unit (CTU) in pre-addressed envelopes provided using a prepaid postal service. The measures collected at baseline and follow-up are shown in table 1.

Randomisation

On receipt of the baseline accelerometer at the CTU after 1 week's wear, participants will be randomised. Randomisation will be stratified by site with minimisation by the participant's perceived reason for their referral to the ERS (ie, weight loss, diabetes control, reduce blood pressure, manage lower limb osteoarthritis symptoms, manage low mood/depression) and by self-reported IT literacy level on a visual analogue scale (ie, lower or higher confidence). To maintain allocation concealment, the minimisation procedure will retain a stochastic element and will be conducted using a secure, password protected web-based system.

Blinding

The ERS practitioners should be unaware of trial participants' treatment allocations. Blinding of participants is not possible, given the nature of the intervention. Given that the primary outcome is an objective measure of physical activity recorded by accelerometer, and the secondary outcomes will be assessed by participant self-completion questionnaire, the risk of assessor bias is likely to be negligible in this study. However, to minimise any potential bias, the statistical analysis will be kept blinded and the code for group allocation not broken until the primary and secondary analyses have been completed.

Follow-up

At 4 weeks post-baseline, a short survey on initial uptake of the ERS will be administered via email.

At 4 and 12 months post-randomisation, participants will be sent an accelerometer and questionnaire booklet by post, along with a simple instruction sheet on how to wear the accelerometer, and a prepaid envelope to return the items to the CTU.

To maximise data completeness at follow-up assessments, participants will be sent standard letters/emails from the CTU: (i) 7 days before delivery of the accelerometer, (ii) 3 days into the 10-day recording window as a prompt for the participant to begin wearing the accelerometer (if not already doing so) and (iii) should the accelerometer not have been received at the CTU, at 3 and 5

BMJ Open: first published as 10.1136/bmjopen-2018-022382 on 21 September 2018. Downloaded from http://bmjopen.bmj.com/ on December 23, 2019 at University of Plymouth - Library A. Henderson-Lib & Media Ser. Protected by copyright.

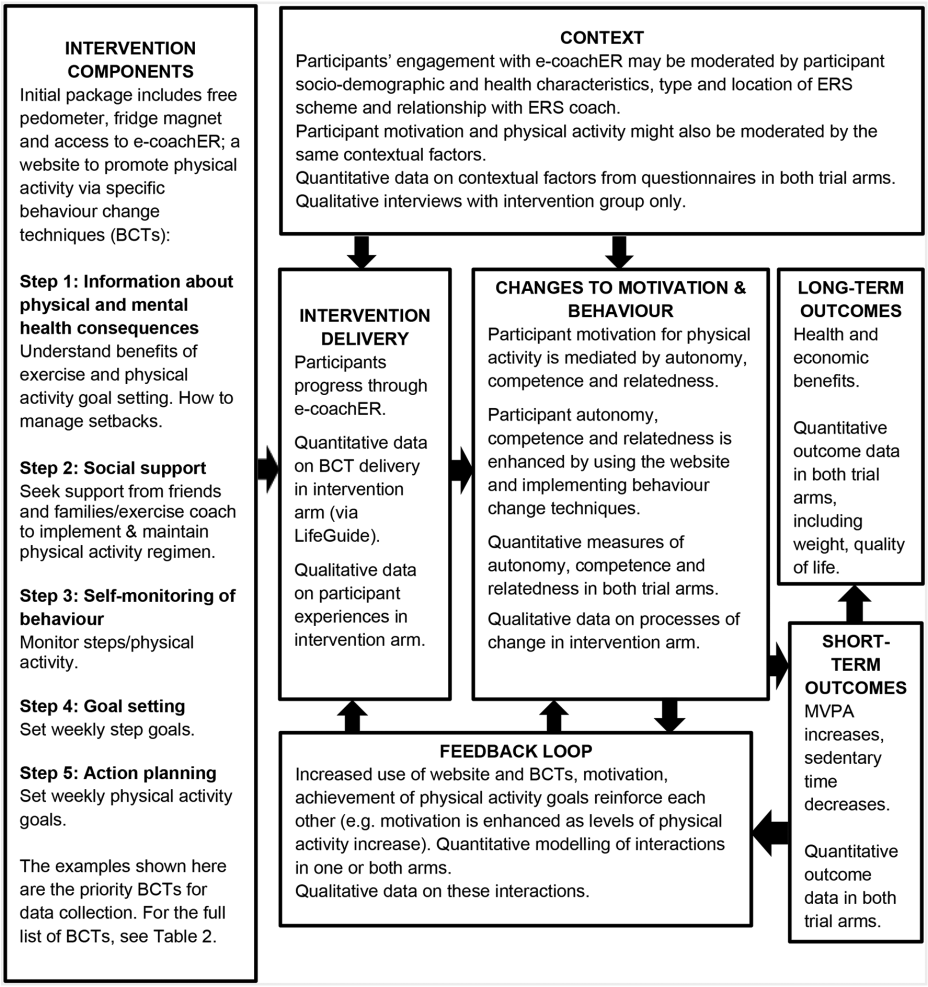

Figure 2 Logic model for e-coachER intervention. ERS, exercise referral scheme; MVPA, moderate- to- vigorous physical activity.

weeks after issue as a reminder to post the accelerometer to the CTU. If the participant has not sent the accelerometer to the CTU after 6 weeks, the local researcher will telephone the participant to remind them to return the device. Participants who return the accelerometer to the CTU will receive an online/high street store voucher for £20 as a token 'thank you', to maximise response rates.

Trial treatment/trial arms

Intervention: web-based support plus ERS (e-coachER)

e-coachER is a web-based support package, which offers a range of interactive opportunities to enhance participants' motivation to take up the ERS and to maintain a more physically active lifestyle, whether or not they engage with their local ERS. A logic model for the intervention is shown in figure 2.

e-coachER is primarily a self-delivered intervention and comprises the following components:

- A mailed 'Welcome Pack' that contains a user guide and the participant's unique user log-in; a simple pedometer (step-counter) and a notepad to record daily physical activity (appended to a magnet with study-specific branding). Participants are encouraged to make use of the pedometer and the activity record sheets for self-monitoring and goal setting in conjunction with the e-coachER website.
- The e-coachER website (on the LifeGuide platform). At the core of e-coachER are seven 'Steps to Health' lasting approximately 5–10 min each, designed to: encourage participants to think about the benefits of physical activity (motivation); seek support from an ERS practitioner, friends/family and the internet (support/relatedness); set progressive goals; self-monitor physical activity with a pedometer and upload step counts or minutes of MVPA (self-regulation, building confidence/autonomy); find ways to increase physical activity more sustainably in the context of day-to-day life and deal with setbacks (building confidence). The sequential content, objectives and how this was

BMJ Open: first published as 10.1136/bmjopen-2018-022382 on 21 September 2018. Downloaded from http://bmjopen.bmj.com/ on December 23, 2019 at University of Plymouth - Library A. Henderson-Lib & Media Ser. Protected by copyright.

implemented were mapped against a taxonomy for behaviour change techniques[30] (table 2). Self-determination theory underpins the intervention with core aims in every step and interaction with participants, aiming to build confidence, autonomy and relatedness.[31]

Participants are encouraged to use the e-coachER support package as an interactive tool by using preset or user-defined reminders to promote ongoing use of functions such as recording weekly physical activity (minutes of MVPA) and goal setting, and receive messages of encouragement. Prompts are sent to remind participants to review their goals. An absence of engagement (eg, failure to review a goal, or not signing into the website for 1, 2 and 4 weeks) triggers reminder emails to the participant.

The website content will be locked prior to starting recruitment, with the exception of webpages displaying links to reputable generic websites for further information about the chronic conditions of interest and lifestyle, links to other websites and apps for self-monitoring health behaviour and health as well as modifiable listings of local opportunities to engage in physical activity.

An avatar is used throughout the content to avoid having to represent a range of individual characteristics such as age, gender and ethnicity. The avatar delivers brief narratives to normalise and support behaviour change and encourage use of the e-coachER support package.

- To maximise accessibility and usage, a local researcher will provide technical support if requested. If a participant does not register on the e-coachER website within the first few weeks, the local researcher will contact the participant to offer support to register. If a participant requires technical support to resolve operational issues with the website (eg, requires a password to be reissued), participants will be referred to a centralised technician within the LifeGuide team.

Intervention development, including piloting the Welcome Pack and developing an initial version of e-coachER, was built on wide ranging experiences from the development of other self-management interventions using the LifeGuide platform,[32] and beta-testing over 7 months with input from service users. Co-applicants and researchers then provided feedback on a time-truncated version of the e-coachER website, and ERS patients provided feedback on a real-time version, for 5 months before the website was locked for the randomised controlled trial.

Usual care

There is currently no single model for ERS in the UK, but the predominant modes of delivery involve referral to a programme (eg, 10–12 weeks) of structured, supervised exercise at an exercise facility (eg, gym or leisure centre) or a counselling approach to support patients to engage in a variety of types of physical activity.[11] ERS operate diversely to accommodate patient choice and local availability of facilities, the common goal being to reduce the risk of long-term metabolic, musculoskeletal and mental health conditions due to physical inactivity. The three participating sites were selected from different regions of the UK (different ERS providers) to provide diversity of approach; the schemes are described in table 3.

Determination of sample size

In the absence of a published minimally important difference for MVPA, assuming a 'small' to 'moderate' standardised effect size of 0.35, we estimated that 413 participants are required at 88% power and a two-sided alpha of 5% assuming 20% attrition, or 90% power at a two-sided alpha of 5% allowing for 16% attrition (using 'sampsi' in STATA V.14). Given that the intervention is being delivered at the level of the individual participant, clustering has not been factored into the sample size calculation. Based on the baseline SD for MVPA total weekly minutes in ≥10 min bouts of 104–113,[33] an effect size of 0.35 would correspond to a between-group difference of 36–39 min of MVPA per week.

Measures

Primary outcome measure

The primary outcome is the number of weekly minutes of MVPA, in ≥10 min bouts, measured objectively by GENEActiv Original accelerometer,[34] over 1 week at 12 months post-randomisation compared with the control group. To be included participants need to provide activity recorded over 4 days, including a weekend day, for at least 16 hours/day.

Additional measures

- Total weekly minutes of MVPA in ≥10 min bouts, measured objectively by accelerometer, over 1 week at 4 months.
- Achievement of at least 150 min of MVPA, measured objectively by accelerometer, over 1 week at 4 and 12 months.
- Achievement of at least 150 min of MVPA over 1 week using the self-reported 7-day Physical Activity Recall Questionnaire at 4 and 12 months.
- Self-reported weekly minutes of MVPA at 4 and 12 months.
- Average daily hours of sedentary behaviour measured objectively by accelerometer over 1 week at 4 and 12 months.
- Self-reported average daily hours of sleep over 1 week at 4 and 12 months.
- Self-reported health-related quality of life, assessed by the EQ-5D-5L[35] and SF-12v2[36] at 4 and 12 months.
- Self-reported symptoms of anxiety and depression, assessed by the Hospital Anxiety and Depression Scale[37] at 4 and 12 months.
- Uptake of the ERS by participant self-report at approximately 4 weeks and at 4 months, and from ERS records.

BMJ Open: first published as 10.1136/bmjopen-2018-022382 on 21 September 2018. Downloaded from http://bmjopen.bmj.com/ on December 23, 2019 at University of Plymouth - Library A. Henderson-Lib & Media Ser. Protected by copyright.

Table 2 The e-coachER sequential process and objectives mapped against behaviour change techniques, and explanation of the implementation strategy

Sequential process	Performance objectives	Behaviour change techniques[30]	Implementation strategy
Welcome pack, pedometer and Introduction to web-based support for self-directed physical activity	To introduce the user to the philosophy of the website to become personal coach.<br>Build on personal support provided by ERS using web-based platform.<br>Support those who do not want to/cannot engage with ERS personnel.<br>Support achievement of personal goals for physical activity to enhance health.	10. Self-monitoring	Explain philosophy of using website to become own personal coach.<br>Links provided to local services and other self-help resources to highlight patient autonomy and choice.<br>Offers e-coachER facilitator to help with using technology. Provide link to IT support from LifeGuide team.
Step 1: thinking about the benefits of physical activity	Elevate importance of physical activity.	82. Information about health consequences<br>83. Information about emotional consequences	Quiz to engage participants using positive framing.<br>Provide evidence of multiple benefits of physical activity, especially for relevant health condition(s).<br>Elicit and address concerns about physical activity, describing support given as part of ERS and by website.
Step 2: support to get active	To encourage user to access and create social support networks.<br>To encourage user to take advantage of ERS and face-to-face support offered.	1. Social support (practical)<br>2. Social support (emotional)<br>3. Social support (unspecified)	Explain how to make the most out of the ERS support to learn how to become own personal trainer in future.<br>Explain how user can create a personal 'physical activity challenge' and share it with family, friends, peers and exercise and health professionals. The patient may be encouraged to tell others about how e-coachER has been used to support behaviour change.<br>Suggest ways of involving family or friends in long-term support for continued physical activity.<br>Link to online sources of local support (eg, local walking or jogging group, or British Trust for Conservation Volunteers).<br>How to use website to send personalised email/text reminders, motivational messages to self.<br>Draw on positive normative beliefs; identify benefits of social interaction (companionship). Sharing personal physical activity challenge with others, involve friends and family, online local support links.<br>Identify benefits of informational support (from ERS) in addition to emotional support from family and friends.
Step 3: counting your steps	To encourage and support the user to monitor step counts using a pedometer over a week.<br>Emphasise personal experimentation.	10. Self-monitoring of behaviour	Provide guidance on how to count steps/use pedometer.<br>Provide guidance on how steps can be implemented into lifestyle.<br>Encourage self-monitoring using diary.
Step 4: making your step plans	To set explicit step count goals for the following week.	66. Goal setting (behaviour)	Give rationale and evidence for goal-setting for graded increase in physical activity.<br>User sets specific, achievable goals for next week (eg, sessions completed, step count using the supplied pedometers).<br>Links provided to local services and other resources.
Step 5: making your activity plans	To encourage and support the user to identify behavioural goals (types of activities).	68. Action planning	User selects walking or 'other physical activities' (which includes options for facility-based activity with practitioner support within ERS).<br>Present options for facility and lifestyle-based activity.<br>Sets specific, achievable goals for next week with a particular focus on avoiding days with less activity by planning walking or other activities.<br>Keeping a physical activity diary.

Continued

BMJ Open: first published as 10.1136/bmjopen-2018-022382 on 21 September 2018. Downloaded from http://bmjopen.bmj.com/ on December 23, 2019 at University of Plymouth - Library A. Henderson-Lib & Media Ser. Protected by copyright.

Table 2 Continued

Sequential process	Performance objectives	Behaviour change techniques[30]	Implementation strategy
Weekly goal and physical activity review	To promote adherence and graded increase in physical activity by providing tailored feedback and advice based on self-reported goal progress.	66. Goal setting behaviour<br>68. Action planning<br>69. Review behaviour goals	User records extent to which goals achieved in previous week, gets progress graph and personalised feedback.<br>Praise for any goal achievement, encouragement to set a more challenging goal if not yet meeting target physical activity criteria.<br>Encouragement where goals not attained, with links to webpages to assist with increasing motivation or confidence, selecting different activities or goals, making better plans, accessing support, overcoming setbacks (with links to relevant sessions below).<br>Each session completed ends with new links to reputable information and resources (eg, NHS choices, condition-specific physical activity advice websites).<br>Help user plan gradual increases in physical activity.
Step 6: finding ways to achieve your plans	To help the user harness their environment to provide support for physical activity.<br>Identifying personal motivations, building confidence.	30. Restructuring the physical environment<br>31. Restructuring the social environment<br>32. Avoidance/reducing exposure to cues for behaviour	Make plan to use environment to automatically support physical activity (eg, fitness equipment in living room, route to work/shops that involves more physical activity, committing self to specific routine).<br>Advise user on how to use website to send personalised email/text reminders, motivational messages.<br>Overcoming barriers in work, leisure, home and travel. Building self-efficacy.<br>Using smart phone apps for mobile support (eg, PowerTracker (c), MyFitnessPal (c)).<br>Invite user to identify personal motivations for becoming more active.
Motivational messages (text and/or emails)	To provide reminders of user's personal reasons (not necessarily health reasons) for becoming more active.	15. Prompts/cues	Invite user to write motivational message to be sent weekly or monthly detailing their own motivations for becoming more active.
Step 7: dealing with setbacks	To provide strategies for overcoming relapse in levels of physical activity.	5. Reduce negative emotions	Identify possible causes of relapse (eg, illness, holidays, change in work hours, new caring responsibilities) and plan ways to overcome barriers.<br>Challenging catastrophic negative thoughts about lapses from intended physical activity.<br>How to learn from a lapse and plan to avoid or overcome in future.<br>Provide salient role models of people overcoming barriers to successfully engage with physical activity.

ERS, exercise referral scheme.

BMJ Open: first published as 10.1136/bmjopen-2018-022382 on 21 September 2018. Downloaded from http://bmjopen.bmj.com/ on December 23, 2019 at University of Plymouth - Library A. Henderson-Lib & Media Ser. Protected by copyright.

Table 3 Characteristics of the local ERS involved in the study

	South West England (predominantly Plymouth)	West Midlands (Birmingham)	Greater Glasgow and Clyde (GGC) Health Board Area
Population of city/ locality and general characteristics	264 000<br>93% White British.<br>Average age is 39 years.<br>Plymouth has higher than average levels of poverty and deprivation (26.2% of population among the poorest 20.4% nationally).<br>Life expectancy, at 78.3 years for men and 82.1 for women, is the lowest of any region in the South West of England.	1 244 438<br>White British (53.1%), Pakistani (13.5%) and Indian (6%). Birmingham is ranked the sixth most deprived local authority in the UK.<br>Approximately 40% of the population lives in highly deprived areas.<br>The average life expectancy in Birmingham is 77.1 years for males and 81.9 years for females.	1 161 370<br>GGC is the largest health board in the UK, comprising six local authority areas: 92.5% white, 5.3% Asian, Asian Scottish or Asian British, 1.2% African, 0.2% Caribbean or black, 0.4% mixed, 0.4% other.<br>There is large variation in deprivation across GGC, but as a whole, it experiences higher than average levels of deprivation and poverty (34.4% population among the poorest 12.4% national average).<br>Life expectancy at 74.9 years for males and 80.0 years for females, is the lowest in Scotland.
Number of centres/ facilities where referrals are made to in the ERS	One main ERS run by Everyone Active in Plymouth and two smaller ones in rural locations.<br>Referrals for ERS came from 31 local GP practices.	One main ERS, Be Active Plus run by Birmingham City Council Wellbeing Service.<br>Referrals for ERS came from 286 local GP practices.	One main ERS (Live Active) delivered by six local leisure trusts in six local authority areas of GGC (Glasgow, East Renfrewshire, Renfrewshire, East Dunbartonshire, West Dunbartonshire and Inverclyde).<br>Referrals are possible from any health professional in primary and secondary care.
Weeks, sessions and general details about ERS	Schemes vary from 6 to 12 weeks, attendees should commit to a minimum of two sessions/week in the gym with drop-in swimming, aquafit and gentle exercise group sessions available to all.<br>All ERS referrals are risk assessed as low or medium risk. Those classed at medium risk may only attend a supervised session. Additionally, a 'walking for health' scheme is highlighted by one ERS provider.	Patients meet with a health and fitness advisor to discuss their preferences for physical activity and an individually tailored 12-week exercise programme is designed for them.<br>Activities include the use of gyms, swimming, fitness classes, badminton and table tennis. The gyms are local authority or privately owned. Privately owned gyms are obliged to offer their facilities to Be Active Plus participants.<br>Patients are also told about activities such as the use of parks and open spaces in Birmingham and walking to work, etc.<br>Participants are also contacted after 3 and 6 months and a report is sent to their GP at their 12-week exit interview.	Patients meet with an ERS advisor for behavioural change support and to design a suitable physical activity plan.<br>Patients are given information on a variety of physical activity options including those offered by leisure centres (eg, fitness classes, swimming, gym, etc) as well as health walks, home exercise, active travel, apps, etc and are able to offer specialist guidance on activities suitable for those with medical conditions and/or disabilities.<br>Patients assessed as high risk at referral are screened by a cardiologist prior to being accepted to the scheme.<br>There are fixed contact points of 1, 3, 6 and 12 months, but patients can choose how often they wish support (telephone, email or face-to-face) from the advisor in addition to these over a 12-month period.

Continued

BMJ Open: first published as 10.1136/bmjopen-2018-022382 on 21 September 2018. Downloaded from http://bmjopen.bmj.com/ on December 23, 2019 at University of Plymouth - Library A. Henderson-Lib & Media Ser. Protected by copyright.

Ingram W, et al. BMJ Open 2018;8:e022382. doi:10.1136/bmjopen-2018-022382

Table 3 Continued

	South West England (predominantly Plymouth)	West Midlands (Birmingham)	Greater Glasgow and Clyde (GGC) Health Board Area
Cost for patients in ERS (if applicable)	Costs vary related to age/concessions. 3 months ERS costs between £14.90 and £70 inclusive of all activities. Pay as you go: £2.10–£3.50 per session.	Patients are not charged for their assessment and support by the health and fitness advisor. The costs of the programme depend on chosen activities and leisure centre attended. Patients in receipt of state benefits or tax credits are eligible for a Passport to Leisure which entitles them to a 30% discount on most activities offered at Birmingham City Council run leisure centres, well-being centres and swimming pools. They can attend free Be Active sessions which take place at restricted times in leisure centres.	Live Active behavioural support is free to the patient for 12 months. If patients wish to use leisure facilities, they are entitled to access this at a concessionary rate (usually around 30% reduction).
Number of people referred to local ERS from 1 August 2015 to 31 March 2017 (ie, during the recruitment period of the study)	300	3470	6500
Most common primary reason for referrals (1 August 2015 to 31 March 2017)*	Depression/anxiety/stress: 24%	BMI>30: 28%	BMI≥30: 58%

*The data on primary reason for referral are subjective as many patients have multiple conditions and a practitioner may favour recording one condition (eg, obesity) rather than another (eg, low mood). Within the respective schemes, the quality of recording the referral reason also appears to be variable.
BMI, body mass index; ERS, exercise referral scheme; GP, general practitioner.

BMJ Open: first published as 10.1136/bmjopen-2018-022382 on 21 September 2018. Downloaded from http://bmjopen.bmj.com/ on December 23, 2019 at University of Plymouth - Library A. Henderson-Lib & Media Ser. Protected by copyright.

- Adherence to physical activity, using a composite measure to describe the proportion in each arm of the trial that achieved at least 150 min of MVPA in bouts of at least 10 min at 4 months and were still doing so at 12 months.

Self-reported survey process measures

- Single and multiple items, using Likert scales, to assess self-efficacy/confidence to be physically active, importance of being physically active, relatedness (perceived frequency and availability of support), perceived autonomy/control over physically active choices, involvement in self-monitoring and planning to do physical activity.
- In the intervention group, measures of engagement with e-coachER including whether or not the participant visits the website at least once, and whether they reach a stage of the online support to indicate they have set and reviewed at least one physical activity goal. Experience from engagement with other LifeGuide online interventions suggests there may not be an optimum dose of engagement.

Economic evaluation

- Cost-effectiveness. Incremental cost of the intervention to the NHS and incremental cost per change in minutes of MVPA (in ≥10 min bouts) and per QALY.
- An economic evaluation of e-coachER will be undertaken using NHS, personal social services, and patient perspective. The analysis will be twofold—short-term (within-trial) cost-effectiveness analysis (from baseline to 12 months postrandomisation) and long-term cost-effectiveness analysis (beyond-trial modelling of long-term expectations for cost-effectiveness), for e-coachER against ERS. The main outcome of the economic analysis will be an incremental cost per QALY (based on EQ-5D-5L). The short-term cost-effectiveness analysis will use resource use data for development of training of and input from a local LifeGuide facilitator, and central LifeGuide technician; provision and running of the exercise sessions at leisure centres and health and personal social service use. Data will be collected using the e-coachER monitoring system, key informant interviews (including trial manager), review of trial management records and participants' questionnaires at baseline, 4 and 12 months. Unit costs will be taken from the NHS reference costs (eg, DH 2015/2016),[38] standard unit costs[39] and published literature. The long-term cost-effectiveness of e-coachER will be based on an existing policy-relevant decision analytical model.[40 41] The analysis will account for the impact of physical activity on lifetime risk of developing coronary heart disease, stroke and type 2 diabetes.

Process evaluation

The barriers to, and facilitators for, recruitment will be explored with participants in the early stages of the trial through qualitative interviews with local researchers at each site, and also via local researcher field notes of conversations with participants at various stages of the trial. Along with relevant supporting literature, this information will be used to optimise recruitment during the remainder of the trial.

Following guidelines for evaluating complex interventions,[42] a nested mixed methods process evaluation will be undertaken, focussing on identifying factors relating to recruitment, engagement, acceptability, mechanisms and fidelity.

The assessment of barriers and facilitators in recruitment will involve the following:

1. Interviews with researchers about patient-reported reasons for joining the study or not;
2. Interviews with researchers about barriers to recruitment in the primary care setting, and among exercise referral practitioners.

The logic model shown in figure 2 will guide the process evaluation of the intervention. The logic model shows the types of data that will be collected, as well as the causal pathways proposed to contribute to behaviour change and intervention outcomes.

The assessment of intervention engagement and acceptability will involve the following:

1. Semi-structured interviews with up to 10% of the intervention group participants. A purposeful sampling framework will be used to ensure participants with a range of characteristics (gender, age, underlying health condition and trial centre) are invited to take part. Interviews will be conducted at different stages of participation in the trial, with each individual being invited to participate in telephone interviews and if appropriate follow-up interviews (up to a maximum of three telephone interviews over the course of the intervention period (approximately 4 months). Interviews will be recorded and transcribed and personal data or ways of identifying participants removed. Transcriptions will be imported into NVivo for data management purposes. The interview transcripts will be coded and thematic analysis performed to identify key findings. Analysis will initially focus on 'top level' themes, reflected in the intervention logic model. Analysis will follow the principles of Framework Analysis.[43] Further in-depth analysis will also be undertaken in order to ensure emergent data, for example, from longitudinal cases, or condition-specific themes, are explored fully. The focus of the interview questions will be linked to the phase of the intervention, and seek to identify the perceived value of the 'Welcome Pack' and contents in helping to access e-coachER, the overall web-based support and each of the Steps to Health, in terms of functionality and utility to support behaviour change. Participants will be asked to identify if and how they thought e-coachER provided support for their ERS, and maintaining physical activity in addition to and beyond the ERS support. Ideas for additions or revisions to e-coachER will be requested. Questions will also focus on the

BMJ Open: first published as 10.1136/bmjopen-2018-022382 on 21 September 2018. Downloaded from http://bmjopen.bmj.com/ on December 23, 2019 at University of Plymouth - Library A. Henderson-Lib & Media Ser. Protected by copyright.

participants' perceived development of self-regulatory skills (eg, self-monitoring, goal setting) and the extent to which the intervention enhanced a sense of competence, autonomy and relatedness, thereby linking back to the aims and guiding principles of the e-coachER intervention.
2. The researchers will be asked to maintain field notes on any interactions with participants concerning engagement with the intervention, such as any difficulties faced with accessing the intervention website. Semi-structured interviews will be conducted by the qualitative researcher with the researchers at each recruitment site to identify participant barriers and facilitators to using e-coachER.
3. Engagement with the web-based e-coachER support system will be quantified. Metrics such as whether the participant registered, how far they progressed in the seven Steps to Health, visits to and time spent on different web pages and within each of the respective Steps, number of times step counts or amount of physical activity (eg, MVPA) were entered into e-coachER (ie, self-monitoring) and number of times goals were achieved and reviewed.
4. Changes in the process measures (see above) (eg, self-efficacy/confidence to be and importance of being physically active) from baseline to 4 and 12 months follow-up will be assessed and compared between intervention arms.
5. Mediation analysis to determine the extent to which changes in the process measures mediate the effect of the intervention on changes in physical activity at 4 and 12 months.

Data handling

Data will be collected and stored in accordance with the Data Protection Act 1998/General Data Protection Regulation 2018.

Subject numbering

Following receipt of expression of interest, each patient will be allocated a unique number and will then be identified in all study-related documentation by their identification number and initials. A record of names, addresses, telephone numbers and email addresses linked to participants' identification numbers will be stored securely on the study database for administrative purposes only.

Data collection

Data will be recorded on study-specific paper-based case report forms (CRFs) by the local researcher, and participants will complete a paper-based questionnaire booklet comprising validated and non-validated self-report outcome measures (listed in table 1).

Accelerometers will be configured for use prior to issue to participants by the local researcher at baseline and the CTU thereafter, using GENEActiv software. A recording window of 10 days, recording at 75 Hz, will be preset, thus accounting for transits in the post while optimising the battery life of the device.

Accelerometers received by the CTU following 1 weeks' wear by the participant will be physically cleaned with liquid detergent (according to manufacturer's instructions) before data are downloaded via GENEActiv software and linked to participant identification number. Accelerometers will then be issued to other participants in the trial as required.

Data on participants' uptake of the ERS will be collected via a single use token-based authenticated email sent to participants at 4 weeks post-baseline. This will be a short survey requesting information on whether the participant has attended the initial consultation with the ERS advisor, and predefined reasons for non-attendance status, for example, appointment has been booked but not yet attended.

All persons authorised to collect and record study data at each site will be listed on the study site delegation logs, signed by the Principal Investigator.

Data entry

Original CRFs and questionnaire booklets will be posted to the CTU, with copies of the CRF retained at the study site. All data will be double-entered by CTU staff on to a password-protected SQL Server database and encrypted using Secure Sockets Layer. Double-entered data will be compared for discrepancies using a stored procedure and discrepant data will be verified using the original CRF. Incomplete, incoherent, unreadable or other problem data in the CRF pages will be queried by the CTU with study site staff during data entry to ensure a complete and valid dataset. Self-reported data in the questionnaire booklet will not be queried with participants.

The CTU may complete further validation of data items, perform logical data checks and raise further data queries after data collection has been completed. The final export of anonymous data will be transferred to statisticians for analysis after all data cleaning duties have been performed by the CTU.

Data analysis plan

All analyses will be carried out using a detailed a priori statistical analysis plan. Analyses will be reported in full and in accordance with the Consolidated Standards of Reporting Trials (CONSORT) guidelines.[44] Recruitment, uptake of the ERS, engagement with the intervention, outcome completion rates and study withdrawal will be reported (with 95% CIs). Baseline characteristics in the two trial arms will be reported.

The primary analysis will compare complete case outcomes between intervention and control arms groups according to the principle of intention to treat (ie, according to original randomised allocation) at 12 months adjusting for baseline outcome values and stratification and minimisation variables (recruitment site and disease indication).

Secondary analyses will be undertaken to compare groups at follow-up across all follow-up points (ie, 4 and

BMJ Open: first published as 10.1136/bmjopen-2018-022382 on 21 September 2018. Downloaded from http://bmjopen.bmj.com/ on December 23, 2019 at University of Plymouth - Library A. Henderson-Lib & Media Ser. Protected by copyright.

12 months) using a mixed effects repeated measures approach. In addition, we will seek to undertake secondary per-protocol analyses using a complier average causal effect approach to examine the impact of different levels of the adherence to the intervention.

Accelerometry data will be analysed with bespoke software to classify data into levels of physical activity intensity using accepted cut-points. Standard operating procedures will be applied to make a decision about dealing with missing data.

The primary analysis model will be extended to fit interaction terms to explore possible subgroup differences in intervention effect in stratification and minimisation variables and the predefined baseline characteristics. As not formally powered, these subgroup analyses will be regarded as exploratory and hypothesis-generating.

Sensitivity analysis, using multiple imputation and assuming unobserved measurements are missing at random will be conducted for both primary and secondary analyses to assess the likely impact of missing data on the primary and secondary outcomes at 12 months. Contemporary mediational analysis methods[45] will be used to explore the impact of process outcomes identified in the planned intervention components, including engagement, use of behaviour change techniques and motivation and processes of change (eg, self-efficacy, autonomy, relatedness).

No interim analysis of primary or secondary outcomes is planned. No adjustment of p-values will be made to account for multiple testing, although the implications of multiple testing will be considered when evaluating the results of the analyses. Analysis of the primary outcome will be performed prior to all other analyses. All analyses will be undertaken using STATA V.14.2.

Checks will be undertaken to assess the robustness of models, including assessment of model residual normality and heteroscedasticity.

Patient and public involvement

The research question was informed by patient and public involvement (PPI) over many years. Individual and group interviews were conducted with patients to identify the barriers and facilitators associated with ERS, and what additional support could help maintain physical activity for a variety of chronic conditions. Our extensive engagement with ERS practitioners allowed us to understand the individual variability and collective patient experience of ERS. This included one of the authors developing, delivering and adapting a training course for ERS practitioners based on their feedback.

The LifeGuide team worked extensively with PPI representatives to develop the appropriate support, concluding that ERS patients would appreciate additional support from an ERS to help them to further develop the independent motivation to maintain physical activity, involving a broad range of active options. Also, patients widely indicated that the LifeGuide web-based system can provide appropriate support for making health behaviour changes. Typically ERS can increase health inequalities by limiting access to those who have limited disposable income or have restricting physical and mental health conditions. The e-coachER system was designed to support those with such restrictions.

Patients were involved in the design of the study. A PPI group was involved in the initial development and refinement of the e-coachER web-based behavioural support. Patients with experience of being referred for an exercise programme, took part in focus groups and provided direct feedback on iterations of the e-coachER intervention during its development.

We engaged with over 20 ERS patients who volunteered to pilot the e-coachER Welcome Pack and provide feedback on the e-coachER website. A PPI representative was available to provide opinions on the study protocol and patient-facing documentation (eg, Participant Information Sheet) during the set-up of the study.

Patients are involved in the oversight of study progress and conduct via representation at periodic Project Management Group meetings and Trial Steering Committee meetings.

Results will be disseminated to study participants. At the end of the trial, a plain English summary of the study results will be made available to participants via a designated webpage on the Peninsula Clinical Trials Unit website, and emailed or posted to participants on request.

Trial monitoring and oversight

A Project Management Group including the Chief Investigator, Principal Investigators, co-applicants, CTU Trial Manager, ERS advisor and PPI representative will meet quarterly to provide multidisciplinary input and oversight for the study.

A Trial Steering Committee (TSC) including an independent chair, independent clinicians and/or academics with relevant expertise, independent statistician/methodologist with relevant expertise and a representative contributing a patient/public perspective will oversee the conduct and scientific integrity of the trial. The TSC will review study progress and protocol adherence. Each committee will function in accordance with agreed terms of reference set out in a charter.

An independent Data Monitoring Committee (DMC) will monitor the safety and ethics of the trial by overseeing recruitment, primary outcome data completeness and serious adverse event data.

The committees will meet once before the start of the trial and approximately annually thereafter.

ETHICS AND DISSEMINATION

Safety considerations

The recording and reporting of non-serious adverse events in this study will not be required. Serious adverse events (SAE) will be captured via survey-specific items on hospital admissions in the questionnaire booklet at 4 and 12 months, that is, reason and duration of the inpatient

BMJ Open: first published as 10.1136/bmjopen-2018-022382 on 21 September 2018. Downloaded from http://bmjopen.bmj.com/ on December 23, 2019 at University of Plymouth - Library A. Henderson-Lib & Media Ser. Protected by copyright.

stay, and self-reported relatedness of the SAE to participation in the trial; self-report independent of the questionnaire booklet; notification to the local researcher by the participant's relative/advocate or notification by the participant's GP.

Reports of SAEs will be provided to the CTU. The CTU will liaise with the local researcher who will be responsible for ascertaining further details about the SAE as appropriate. The Chief Investigator will report any SAE that is related (definitely, possibly or probably related) to the research procedures to the Research Ethics Committee within 15 days of becoming aware of the event. The CTU will prepare quarterly summaries of SAEs for review by the independent DMC and Sponsor.

Dissemination plan

The findings of the study will be made publicly available through publication in relevant peer-reviewed journals and the NIHR Journals Library website; and presentation to the scientific community, patient support groups, the ERS services and NHS strategy forums at local and national level. The study is reported in accordance with CONSORT guidelines for publishing randomised trials and TIDieR guidelines for intervention reporting.

A plain English summary of the main study results will be made available for participants and other lay audiences.

Changes to the protocol after the start of the trial

Primary outcome measure and sample size

The original protocol featured an internal pilot. During the internal pilot phase, 180 patients were to be recruited over 3 months to provide sufficient information to justify progression to a main trial. Progression from the internal pilot to the main trial was dependent on recruitment rate and engagement with the intervention according to the scenarios in table 4. In the main trial, an additional 1220 participants were to be recruited, giving a total of 1400 participants (recruited over 16 months).

The recruitment rate during the internal pilot phase was lower than expected, due to limitations on the time primary care practitioners had available to approach potential participants; delayed start at one of the research sites; poor uptake when patients were approached via a postal mailshot; high ineligibility rate among patients who were identified via a primary care database. In response to poor recruitment, the following strategies to increase recruitment were introduced:

- The inclusion criterion for BMI was aligned with the ERS entry (upper BMI limit for the trial was originally 35 and was raised to 40), and prediabetes was included as an inclusion criterion.
- Recruitment via the ERS service, which was already taking place at the site in Greater Glasgow, was adopted in the West Midlands and the South West in addition to recruitment via primary care.
- Incentive payments to participants (for returning an accelerometer) were increased from £10 to £20 per accelerometer.

Having implemented these measures, the conditions for progression in terms of recruitment rate and engagement with the intervention were not met by the end of the internal pilot phase, despite a 4-month extension period. A 'recovery plan' was developed in collaboration with the funders, based on amending the choice of primary outcome, and submitted in May 2016.

The original primary outcome was achievement of at least 150 min of MVPA measured objectively by accelerometer over 1 week at 12 months. This outcome was based on the findings of a systematic review of ERS[12 46] demonstrating that trials had primarily reported their outcomes according to percentage of participants reaching the National Institute for Health and Care Excellence guidelines for physical activity level, that is, 150 min of MVPA per week. We estimated that recruiting 700 participants per group would allow us to detect a difference at 12 months follow-up of at least 10% (intervention group: 53% vs control group: 43%), assuming an attrition rate of 20% and small effect of clustering (intracluster correlation coefficient ICC: 0.006) at 90% power and 5% alpha. Thus, the original sample size was 1400 participants, to be recruited over 16 months.

From the outset, the TSC and DMC had recommended that this dichotomous primary outcome measure be replaced with a continuous variable; total weekly minutes of MVPA. This was because:

a. A continuous primary outcome measure would be more relevant in this study population, in terms of detecting a small but clinically significant increase in minutes of MVPA.

Table 4 Internal pilot to main trial progression rules

Criteria	Scenario 3	Scenario 2	Scenario 1
% of internal pilot sample size target (180 patients) recruited	<65%	65%–79%	≥80%
Intervention engagement (% participants who access e-coachER at least once)	<65%	65%–79%	≥80%
Proposed action	No progression	Discuss with Trial Steering Committee and funder about progression and resources needed to achieve target.	Proceed to full trial.

BMJ Open: first published as 10.1136/bmjopen-2018-022382 on 21 September 2018. Downloaded from http://bmjopen.bmj.com/ on December 23, 2019 at University of Plymouth - Library A. Henderson-Lib & Media Ser. Protected by copyright.

b. Based on sample size calculations, this would offer greater statistical power than to the categorical assessment of whether participants reach a threshold of 150 min of MVPA. This would therefore afford a reduction in sample size.

The TSC and funders agreed these changes (in August 2016) and the original sample size was reduced in accordance with this new primary outcome measure and revised sample size calculation, from 1400 to 413 participants (to be recruited over 21 months). A similar reduction in sample size has been incorporated into the qualitative component of the process evaluation work.

Current study status

The e-coachER trial began recruiting patients in August 2015 and closed to recruitment in March 2017. Data collection is expected to be completed in March 2018 and results are expected to be published in September 2018.

Author affiliations
[1]Faculty of Medicine and Dentistry, Peninsula Medical School, University of Plymouth, Plymouth, UK
[2]University of Exeter Medical School, Exeter, UK
[3]Department of Clinical Sciences, Brunel University, London, UK
[4]Faculty of Medicine, Southampton University, Southampton, UK
[5]Nuffield Department of Primary Care Health Sciences, Oxford University, Oxford, UK
[6]School of Sport, Exercise and Rehabilitation Sciences, University of Birmingham, Birmingham, UK
[7]Physical Activity for Health Research Centre, University of Edinburgh, Edinburgh, UK
[8]Faculty of Sport and Health, University of St Mark and St John, Plymouth, UK
[9]Patient & Public Involvement, Plymouth, UK
[10]Department of Rheumatology, Royal Cornwall Hospitals NHS Trust, Truro, UK

Acknowledgements The authors would like to thank all the patients and staff who are participating in the trial and also to the members of the Trial Steering Committee and Data Monitoring Committee for their valuable support throughout the lifetime of the research. The authors would like to acknowledge the role of the Clinical Research Network in connection with data collection and also the contribution made by the Department of Health and the Greater Glasgow Health Board in meeting the excess treatment and service support costs associated with the trial. In memory of Nigel Charles, with thanks for his contribution to the qualitative research.

Collaborators Trial Steering Committee Full members: Dr Sharon Simpson, Chair (University of Glasgow); Professor Charlie Foster, Independent Member (University of Oxford then University of Bristol); Dr Mark Kelson, Independent Member (Cardiff University then University of Exeter); Professor John Powell, Independent Member (University of Oxford); Mr Chris Cavanagh, Patient and Public Involvement Representative; Professor Adrian Taylor, Chief Investigator (University of Plymouth) Observers; Professor Rod Taylor, Trial Statistician (University of Exeter); Dr Wendy Ingram, Trial Manager (Peninsula Clinical Trials Unit, University of Plymouth); Mrs Pam Baxter, Sponsor Representative (University of Plymouth) Data Monitoring Committee members; Professor Paul Aveyard (University of Oxford); Dr Anne Haase (University of Bristol); Professor Richard Morris (University of Bristol).

Contributors AHT conceived the idea for the study with RST, NM, KJ, LY, NA, JLC, CG, SGD, PL, AW/JE, BJ, JLC and RBJ. AHT, RHT, NM, KJ, LY, NA, JLC, CG, JV, SGD, CM, PL, JE, BJ, JLC, AW, RBJ, WI and DW contributed to the final study design and development of the protocol. AHT, JDL, MS and LY developed the web-support and led PPI testing and feedback with JK. NA developed the health economics plan. SGD developed the process evaluation plan with CG, NC and RHT. RHT provided the statistical plan. All authors critically revised successive drafts of the manuscript and approved the final version.

Funding This research has been conducted independently by the University of Plymouth, University of Birmingham, Brunel University London, University of Edinburgh, University of Exeter, University of Southampton, Royal Cornwall Hospitals NHS Trust and University of St Mark and St John. It is funded by the Department of Health (DH) as part of the National Institute for Health Research (NIHR) Health Technology Assessment (HTA) programme (project number 13/25/20).

Disclaimer The views expressed are those of the author(s) and not necessarily those of the NHS, the NIHR or the Department of Health.

Competing interests SGD reports personal fees from University College London outside the submitted work. CM reports grants from NIHR during the conduct of the study; grants from ESRC Impact Acceleration Award outside the submitted work; is an employee of the Health Improvement Team (NHS Greater Glasgow & Clyde) who fund and manage the Service Level Agreement for the Exercise Referral Scheme. NM reports grants from various research funders including NIHR during the conduct of the study. LY reports grants from NIHR during the conduct of the study.

Patient consent Not required.

Ethics approval The original study and subsequent amendments were approved by the NHS Research Ethics Committee North West-Preston (REC reference 15/NW/0347).

Provenance and peer review Commissioned bid 13/25 Interventions to enhance engagement in exercise referral schemes.

Open access This is an open access article distributed in accordance with the Creative Commons Attribution 4.0 Unported (CC BY 4.0) license, which permits others to copy, redistribute, remix, transform and build upon this work for any purpose, provided the original work is properly cited, a link to the licence is given, and indication of whether changes were made. See: https://creativecommons.org/licenses/by/4.0/.

REFERENCES

1. Public Health England. *Physical inactivity: economic costs to NHS clinical commissioning groups London*: Her Majesty's Stationery Office, 2016.
2. Department of Health. *Start Active, Stay Active: A report on physical activity from the four home countries' Chief Medical Officers*. London: Department of Health, 2011.
3. NICE. *Obesity: Guidance on the prevention, identification, assessment and management of overweight and obesity in adults and children*. London: National Clinical Guideline Centre, 2010.
4. NICE. *Hypertension: Clinical management of primary hypertension in adults*. London: National Clinical Guideline Centre, 2011.
5. NICE. *Type 2 diabetes: The management of type 2 diabetes*. London: National Clinical Guideline Centre, 2008.
6. NICE. *Osteoarthritis: The care and management of osteoarthritis in adults*. London: National Clinical Guideline Centre, 2008.
7. NICE. *Depression: The treatment and management of depression in adults*. London: National Clinical Guideline Centre, 2009.
8. Bouchard C, Blair SN, Katzmarzyk PT, *et al*. Less sitting, more physical activity, or higher fitness? *Mayo Clin Proc* 2015;90:1533–40.
9. Warburton DE, Bredin SS. Reflections on physical activity and health: what should we recommend? *Can J Cardiol* 2016;32:495–504.
10. Dunstan DW, Daly RM, Owen N, *et al*. Home-based resistance training is not sufficient to maintain improved glycemic control following supervised training in older individuals with type 2 diabetes. *Diabetes Care* 2005;28:3–9.
11. British Heart Foundation National Centre for Physical Activity and Health. *Section 2: A Snapshot of ER Schemes Operating in England, Scotland & Northern Ireland - 2006-2008*: A Toolkit for the Design, Implementation & Evaluation of Exercise Referral Schemes: Loughborough University, 2010.
12. Pavey TG, Taylor AH, Fox KR, *et al*. Effect of exercise referral schemes in primary care on physical activity and improving health outcomes: systematic review and meta-analysis. *BMJ* 2011;343:d6462.
13. Pavey T, Taylor A, Hillsdon M, *et al*. Levels and predictors of exercise referral scheme uptake and adherence: a systematic review. *J Epidemiol Community Health* 2012;66:737–44.
14. Rouse PC, Ntoumanis N, Duda JL, *et al*. In the beginning: role of autonomy support on the motivation, mental health and intentions of participants entering an exercise referral scheme. *Psychol Health* 2011;26:729–49.
15. Joseph RP, Durant NH, Benitez TJ, *et al*. Internet-Based Physical Activity Interventions. *Am J Lifestyle Med* 2014;8:42–67.
16. Devi R, Singh SJ, Powell J, *et al*. Internet-based interventions for the secondary prevention of coronary heart disease. *Cochrane Database Syst Rev* 2015;12:Cd009386.

BMJ Open: first published as 10.1136/bmjopen-2018-022382 on 21 September 2018. Downloaded from http://bmjopen.bmj.com/ on December 23, 2019 at University of Plymouth - Library A. Henderson-Lib & Media Ser. Protected by copyright.

17. Davies CA, Spence JC, Vandelanotte C, *et al*. Meta-analysis of internet-delivered interventions to increase physical activity levels. *Int J Behav Nutr Phys Act* 2012;9:52.
18. Morrison LG, Hargood C, Lin SX, *et al*. Understanding usage of a hybrid website and smartphone app for weight management: a mixed-methods study. *J Med Internet Res* 2014;16:e201.
19. Lloyd S, Dennison L, Morrison L, *et al*. Losing weight online with POWeR: a randomised controlled trial of a web-based behavioural intervention in a community setting. *The Lancet* 2013;382:S62.
20. Williams S, Yardley L, Wills GB. A qualitative case study of LifeGuide: users' experiences of software for developing Internet-based behaviour change interventions. *Health Informatics J* 2013;19:61–75.
21. Yardley L, Morrison LG, Andreou P, *et al*. Understanding reactions to an internet-delivered health-care intervention: accommodating user preferences for information provision. *BMC Med Inform Decis Mak* 2010;10:52.
22. Little P, Stuart B, Hobbs FR, *et al*. An internet-based intervention with brief nurse support to manage obesity in primary care (POWeR+): a pragmatic, parallel-group, randomised controlled trial. *Lancet Diabetes Endocrinol* 2016;4:821–8.
23. Greaves CJ, Sheppard KE, Abraham C, *et al*. Systematic review of reviews of intervention components associated with increased effectiveness in dietary and physical activity interventions. *BMC Public Health* 2011;11:119.
24. Michie S, Abraham C, Whittington C, *et al*. Effective techniques in healthy eating and physical activity interventions: a meta-regression. *Health Psychol* 2009;28:690–701.
25. Anokye NK, Trueman P, Green C, *et al*. The cost-effectiveness of exercise referral schemes. *BMC Public Health* 2011;11:954.
26. Benaissa M, Malik B, Kanakis A, *et al*. *Tele-healthcare for diabetes management: A low cost automatic approach*: Conference proceedings: Annual International Conference of the IEEE Engineering in Medicine and Biology Society IEEE Engineering in Medicine and Biology Society Annual Conference, 2012:1290–3.
27. Chan AW, Tetzlaff JM, Altman DG, *et al*. SPIRIT 2013 statement: defining standard protocol items for clinical trials. *Ann Intern Med* 2013;158:200–7.
28. Hoffmann TC, Glasziou PP, Boutron I, *et al*. Better reporting of interventions: template for intervention description and replication (TIDieR) checklist and guide. *BMJ* 2014;348:g1687.
29. Ahmad S, Harris T, Limb E, *et al*. Evaluation of reliability and validity of the General Practice Physical Activity Questionnaire (GPPAQ) in 60-74 year old primary care patients. *BMC Fam Pract* 2015;16:113.
30. Michie S, Richardson M, Johnston M, *et al*. The behavior change technique taxonomy (v1) of 93 hierarchically clustered techniques: building an international consensus for the reporting of behavior change interventions. *Ann Behav Med* 2013;46:81–95.
31. Deci EL, Ryan RM. *Handbook of self-determination research*. Rochester, New York: University of Rochester Press, 2002.
32. Yardley L, Morrison L, Bradbury K, *et al*. The person-based approach to intervention development: application to digital health-related behavior change interventions. *J Med Internet Res* 2015;17:e30.
33. Harris T, Kerry SM, Victor CR, *et al*. A primary care nurse-delivered walking intervention in older adults: PACE (pedometer accelerometer consultation evaluation)-Lift cluster randomised controlled trial. *PLoS Med* 2015;12:e1001783.
34. Powell C, Carson BP, Dowd KP, *et al*. Simultaneous validation of five activity monitors for use in adult populations. *Scand J Med Sci Sports* 2017;27:1881–92.
35. Herdman M, Gudex C, Lloyd A, *et al*. Development and preliminary testing of the new five-level version of EQ-5D (EQ-5D-5L). *Qual Life Res* 2011;20:1727–36.
36. Ware JE, Kosinski M, Turner-Bowker DM, *et al*. *How to score version 2 of the SF-12 health survey (with a supplement documenting version 1)*. Lincoln, RI; Boston, Mass: QualityMetric Inc; Health Assessment Lab, 2002.
37. Zigmond AS, Snaith RP. The hospital anxiety and depression scale. *Acta Psychiatr Scand* 1983;67:361–70.
38. Department of Health. *NHS Reference Costs 2014 to 2015*, 2015.
39. Curtis LA. *Unit costs for health and social care University of Kent*. Canterbury: Personal Social Services Research Unit, 2014.
40. Anokye NK, Lord J, Fox-Rushby J. Is brief advice in primary care a cost-effective way to promote physical activity? *Br J Sports Med* 2014;48:202–6.
41. Campbell F, Holmes M, Everson-Hock E, *et al*. A systematic review and economic evaluation of exercise referral schemes in primary care: a short report. *Health Technol Assess* 2015;19:1–110.
42. Moore GF, Audrey S, Barker M, *et al*. *Process evaluation of complex interventions: Medical Research Council guidance*. London: MRC Population Health Science Research Network, 2014.
43. Ritchie J, Spencer L. Qualitative data analysis for applied policy research. *Analysing Qualitative Data*. London: Routledge, 1994.
44. Schulz KF, Altman DG, Moher D. CONSORT 2010 statement: updated guidelines for reporting parallel group randomised trials. *BMJ* 2010;340:c332.
45. Emsley R, Dunn G, White IR. Mediation and moderation of treatment effects in randomised controlled trials of complex interventions. *Stat Methods Med Res* 2010;19:237–70.
46. Pavey TG, Anokye N, Taylor AH, *et al*. The clinical effectiveness and cost-effectiveness of exercise referral schemes: a systematic review and economic evaluation. *Health Technol Assess* 2011;15:1–254.

BMJ Open: first published as 10.1136/bmjopen-2018-022382 on 21 September 2018. Downloaded from http://bmjopen.bmj.com/ on December 23, 2019 at University of Plymouth - Library A. Henderson-Lib & Media Ser. Protected by copyright.

e-coachER
Web support for exercise referrals

A multicentre randomised controlled trial of an augmented exercise referral scheme using web-based behavioural support in individuals with metabolic, musculo-skeletal and mental health conditions

STUDY PROTOCOL

Version 6.1 20th November 2017

Chief Investigator:	Prof Adrian Taylor<br>Professor of Health Services Research, University of Plymouth
Study Sponsor:	University of Plymouth
IRAS reference**:**	170179
REC reference:	15/NW/0347
ISRCTN:	15644451
Funder's number:	13/25/20 (NIHR HTA)

This protocol has regard for the HRA guidance

SIGNATURE PAGE

The undersigned confirm that the following protocol has been agreed and accepted and that the Chief Investigator agrees to conduct the trial in compliance with the approved protocol and will adhere to the principles outlined in GCP guidelines, the Sponsor's SOPs, and other regulatory requirements as amended.

I agree to ensure that the confidential information contained in this document will not be used for any other purpose other than the evaluation or conduct of the clinical investigation without the prior written consent of the Sponsor.

I also confirm that I will make the findings of the study publically available through publication or other dissemination tools without any unnecessary delay and that an honest accurate and transparent account of the study will be given; and that any discrepancies from the study as planned in this protocol will be explained.

For and on behalf of the Study Sponsor:

Signature: .... Date: ..../…..../.......

Name (please print): ....

Position: ....

Chief Investigator:

Signature: .... Date: …..../......./.......

Name: (please print):
....

Statistician:

Signature: .... Date: …..../…..../.......

Name: (please print):
....

Taylor A, *et al. Br J Sports Med* 2021; 55:444–450. doi: 10.1136/bjsports-2020-103121

KEY STUDY CONTACTS

Chief Investigator	**Prof Adrian Taylor**<br>Professor of Health Services Research<br>University of Plymouth<br>N6, ITTC Building 1<br>Plymouth Science Park,<br>Plymouth PL6 8BX<br>Email: adrian.taylor@plymouth.ac.uk<br>Tel: 07952 400835
Trial Manager	**Dr Wendy Ingram**<br>Trial Manager<br>Peninsula Clinical Trials Unit<br>University of Plymouth, N16, ITTC Building 1<br>Plymouth Science Park<br>Plymouth PL6 8BX<br>wendy.ingram@plymouth.ac.uk<br>Tel: 01752 315251<br>Fax: 01752 315245
Sponsor	**Plymouth University**<br>Drake Circus<br>Plymouth PL4 8AA<br>Tel: 01752 600600
Sponsor representative	**Prof Jonathan Marsden**<br>FF20 Peninsula Allied Health Centre<br>College of St Mark & St John<br>Plymouth<br>PL6 8BH<br>Email: plymouth.sponsor@plymouth.ac.uk<br>Tel: 01752 587590
Funder	**National Institute for Health Research Health Technology Assessment Programme (NIHR HTA)**<br>National Institute for Health Research<br>Evaluation, Trials and Studies Coordinating Centre<br>University of Southampton<br>Alpha House<br>Enterprise Road<br>Southampton SO16 7NS<br>Email: htacet@southampton.ac.uk<br>Tel: 02380 595586
Clinical Trials Unit	**Peninsula Clinical Trials Unit**<br>University of Plymouth, N16, ITTC Building<br>Plymouth Science Park<br>Plymouth PL6 8BX<br>Email: penctu@plymouth.ac.uk<br>Tel: 01752 439831
Statistician	**Prof Rod Taylor**<br>Professor of Health Services Research<br>University of Exeter Medical School<br>South Cloisters Rm 2.18<br>St. Luke's Campus<br>Exeter EX1 2LU<br>Email: R.Taylor@exeter.ac.uk<br>Tel: 07968 152537

Taylor A, *et al. Br J Sports Med* 2021; 55:444–450. doi: 10.1136/bjsports-2020-103121

STUDY SUMMARY

Study title	A multicentre RCT of an augmented exercise referral scheme (ERS) using web-based behavioural support in individuals with metabolic, musculo-skeletal and mental health conditions.
Short title	e-coachER – adding web-based support to an exercise referral scheme.
Trial design	Multi-centre, individually randomised, two arm trial with internal pilot.
Trial participants	Inactive individuals aged 16-74 years with obesity, hypertension, pre-diabetes, type 2 diabetes, osteoarthritis, or a history of depression, for whom NICE recommends exercise.
Planned sample size	413 participants (206 per trial arm)
Planned study period	45 months (set up 8 months, main recruitment 19 months, follow up 12 months, data cleaning, analysis and reporting 6 months)
Grant start date	01 January 2015
Study aim	To determine whether the addition of a web-based support package to usual ERS increases the minutes of moderate to vigorous intensity physical activity (MVPA) at twelve months, compared with ERS alone, and whether such an intervention is cost-effective.
Primary outcome measure	Total weekly minutes of MVPA in $\geq$10 minute bouts, recorded objectively by accelerometer, over one week at twelve months.
Secondary outcome measures	• Total weekly minutes of MVPA in ≥10 minute bouts, recorded objectively by accelerometer, over one week at four months.<br>• Achievement of at least 150 minutes of MVPA, measured objectively by accelerometer, over one week at four and twelve months post-randomisation.<br>• Average minutes of MVPA, measured by accelerometer over one week at 4 and 12 months post-randomisation.<br>• Self-reported achievement of at least 150 mins of MVPA over one week using the Seven Day Physical Activity Recall Questionnaire at four and twelve months.Self-reported health-related quality of life, assessed by the EuroQol-5 dimension–5 level (EQ-5D-5L) and 12-Item Short Form Health Survey version 2 (SF12v2) at four and twelve months.<br>• Self-reported symptoms of anxiety and depression, assessed by the Hospital Anxiety and Depression Scale (HADS) at four and twelve months.<br>• Average daily hours/minutes of sleep and sedentary behaviour (objectively measured by accelerometer) at baseline, four and twelve months.<br>• Uptake of the ERS by participant self-report at approximately four weeks and four months.<br>• Adherence to the ERS using a composite measure to describe the proportion in each arm of the trial who achieved the primary outcome at four months and were still doing so at twelve months.<br>• Monetary costs of intervention development including the ‘welcome pack’, with a view to costing the (potential) roll-out of the intervention to a wider population.<br>• Self-reported monetary costs of the use of the ERS, and (for the treatment arm) the use of the web-based support package, at four and twelve months.<br>• Mediation measures analysis (i.e. self-reported perceptions of physical activity confidence, importance, autonomy and relatedness, and use of self-monitoring and goal setting).<br>• Moderation analysis, i.e. subgroup analyses for participant characteristics and ERS.<br>• Incremental cost per quality-adjusted life year (QALY) at twelve months.<br>• Measures of engagement with e-coachER, and its content, and use of self-monitoring and goal setting functions, captured on the software platform (LifeGuide).<br>• Qualitative interviews with intervention arm participants focusing on their experiences with ERS and the additional e-coachER intervention.

STUDY SPONSOR AND FUNDER

The study sponsor is University of Plymouth. Selected sponsorship tasks will be delegated to the Plymouth University Peninsula Schools of Medicine and Dentistry (PUPSMD) under the terms of an appropriate service level agreement.

The study was initially funded by a grant of £1,372,155.80 from the National Institute for Health Research (NIHR) Health Technology Assessment (HTA) Programme. This was subsequently reduced to £900,000 in line with a reduced sample size. The grant reference number is 13/25/20. The grant will be held by the University of Plymouth.

ROLES AND RESPONSIBILITIES OF TRIAL OVERSIGHT COMMITTEES

Trial Management Group

A Trial Management Group (TMG) including the Chief Investigator, study statistician, trial manager, health economist, lead for process evaluation, lead for intervention development, and other relevant personnel as required (e.g. data manager, patient representatives, Principal Investigators) will meet regularly. The TMG (and other small working groups such as outcomes group, process evaluation group, recruitment group, intervention development and review group, PPI group) will meet approximately every four weeks in person or by teleconference throughout the set-up and internal pilot of the study to review progress, resolve day-to-day problems and monitor participant recruitment ahead of progression to the full trial. Thereafter the TMG will continue to meet regularly to review and respond to emerging issues, as well as to monitor follow-up, oversee budgetary issues, prepare draft reports, discuss analysis and results, and ultimately the final report. The TMG will report to the Project Management Group.

Project Management Group

A Project Management Group (PMG) including the Chief Investigator, Principal Investigators, co-applicants, Clinical Trials Unit (CTU) trial manager, ERS managers and PPI representative will meet quarterly, usually by teleconference, to provide wider multi-disciplinary input and oversight for the study. Interim communication/discussions will be by telephone or email, as required.

Trial Steering Committee

A Trial Steering Committee (TSC) including an independent chair, independent clinicians and/or academics with relevant expertise, independent statistician/methodologist with relevant expertise and a representative contributing a patient/public perspective will oversee the conduct of the trial. The TSC will meet in person or by teleconference before the start of the internal pilot study, before the start of the main trial and at least annually thereafter (shortly after a Data Monitoring Committee Meeting), to review study progress and protocol adherence, ensure that milestones are achieved and that general scientific probity is maintained. There is the option of the TSC meeting more regularly should either the TSC or research study team think it is necessary. The TSC will function in accordance with agreed terms of reference set out in a TSC Charter.

Data Monitoring Committee

An independent Data Monitoring Committee (DMC) will monitor the safety and ethics of the trial by overseeing recruitment, primary outcome data completeness and adverse event (hospitalisation) data. In addition, the DMC will review data from the internal pilot study to help inform a decision about progression to the main trial. Operating procedures for the DMC will be agreed before the start of the study and incorporated into a DMC charter, updated from time to time as required. The committee will meet once before the start of the internal pilot trial and approximately annually thereafter, by teleconference or face-to-face.

Trial Steering Committee nominations

**Nominated Members**			
**Name**	**Affiliation**	**Expertise/role**	**Email**
Dr Sharon Simpson (Chair)	Glasgow University	Senior Research Fellow (MRC/CSO Social and Public Health Sciences Unit)	Sharon.Simpson@glasgow.ac.uk
Dr Mark Kelson	Cardiff University	Research Fellow in Statistics, South East Wales Trials Unit	KelsonMJ@cardiff.ac.uk
Prof John Powell	Oxford University	Academic public health physician & health services researcher (interest in e-health) expertise	john.powell@phc.ox.ac.uk
Dr Charlie Foster	Oxford University	Nuffield Dept of Population Health; DH policy advisor on physical activity & sport	charlie.foster@dph.ox.ac.uk
Mr Chris Cavanagh	Retired	PPI representative	chriscavanagh58@btinternet.com
**Member**			
Prof Adrian Taylor (CI)	University of Plymouth	Professor of Health Services Research	Adrian.Taylor@plymouth.ac.uk

**Observers**			
**Name**	**Affiliation**	**Expertise/role**	**Email**
Dr Wendy Ingram	Peninsula Clinical Trials Unit	Trial Manager	wendy.ingram@plymouth.ac.uk
Prof Rod Taylor	University of Exeter	Statistician and Trialist	R.Taylor@exeter.ac.uk
Mrs Pam Baxter	University of Plymouth	Sponsor representative	pam.baxter@plymouth.ac.uk

Data Monitoring Committee Nominations

Name	Affiliation	Expertise/role	Email
Prof Paul Aveyard	University of Oxford	Professor of Behavioural Medicine, Department of Primary Care Health Services	paul.aveyard@phc.ox.ac.uk
Dr Anne Haase	University of Bristol	Senior Lecturer in Exercise, Nutrition, and Health, School of Policy Studies	Anne.Haase@bristol.ac.uk
Prof Richard Morris	University of Bristol	Professor in Medical Statistics, School of Social and Community Medicine	Richard.morris@bristol.ac.uk

Taylor A, *et al. Br J Sports Med* 2021; 55:444–450. doi: 10.1136/bjsports-2020-103121

LIST of CONTENTS

SIGNATURE PAGE....1
KEY STUDY CONTACTS....2
ROLES AND RESPONSIBILITIES OF TRIAL OVERSIGHT COMMITTEES....4
LIST of CONTENTS....7
STUDY FLOW CHART....9
1 BACKGROUND & RATIONALE....12
2 OBJECTIVES AND OUTCOME MEASURES....19
4 STUDY SETTING....21
5 ELIGIBILITY CRITERIA....21
6 RECRUITMENT....22
7 BASELINE DATA COLLECTION....24
8 RANDOMISATION....24
9 TRIAL INTERVENTION....25
10 TRIAL ACTIVITIES AND FOLLOW-UP....25
11 SAFETY REPORTING....28
12 STATISTICS AND DATA ANALYSIS....29
13 DATA HANDLING....31
14 MONITORING, AUDIT & INSPECTION....32
15 ETHICAL AND REGULATORY CONSIDERATIONS....32
16 DISSEMINATION POLICY....33
17 REFERENCES....34

LIST OF ABBREVIATIONS

AE	Adverse Event
CI	Chief Investigator
CRF	Case Report Form
DMC	Data Monitoring Committee
ERS	Exercise referral scheme
EQ-5D-5L	EuroQol -5 dimension – 5 level
GCP	Good Clinical Practice
GPPAQ	GP Physical Activity Questionnnaire
HADS	Hospital Anxiety and Depression Scale
ICF	Informed Consent Form
ISF	Investigator Site File
ISRCTN	International Standard Randomised Controlled Trials Number
MVPA	Moderate to vigorous physical activity
NHS R&D	National Health Service Research & Development
OA	Osteoarthritis
PA	Physical Activity
PI	Principal Investigator
PCRN	Primary Care Research Network
PIS	Participant Information Sheet
PMG	Project Management Group
PPI	Patient and Public Involvement
QALY	Quality Adjusted Life Year
RA	Research Assistant
RCT	Randomised Controlled Trial
REC	Research Ethics Committee
SAE	Serious Adverse Event
SF12v2	12-Item Short Form Health Survey version 2
SOP	Standard Operating Procedure
TMG	Trial Management Group
TSC	Trial Steering Committee
TMF	Trial Master File

Taylor A, *et al. Br J Sports Med* 2021; 55:444–450. doi: 10.1136/bjsports-2020-103121

PARTICIPANT PATHWAY

KEY

White boxes: activity at all sites

Orange boxes: activity at South West and Birmingham sites

Green boxes: activity at Glasgow site.

ERS: exercise referral scheme.

EA: Exercise advisor

RA: Research Assistant

PenCTU: Peninsula Clinical Trials Unit.

Referral to the ERS may occur at different points, and this is indicated by parentheses.

Taylor A, *et al. Br J Sports Med* 2021; 55:444–450. doi: 10.1136/bjsports-2020-103121

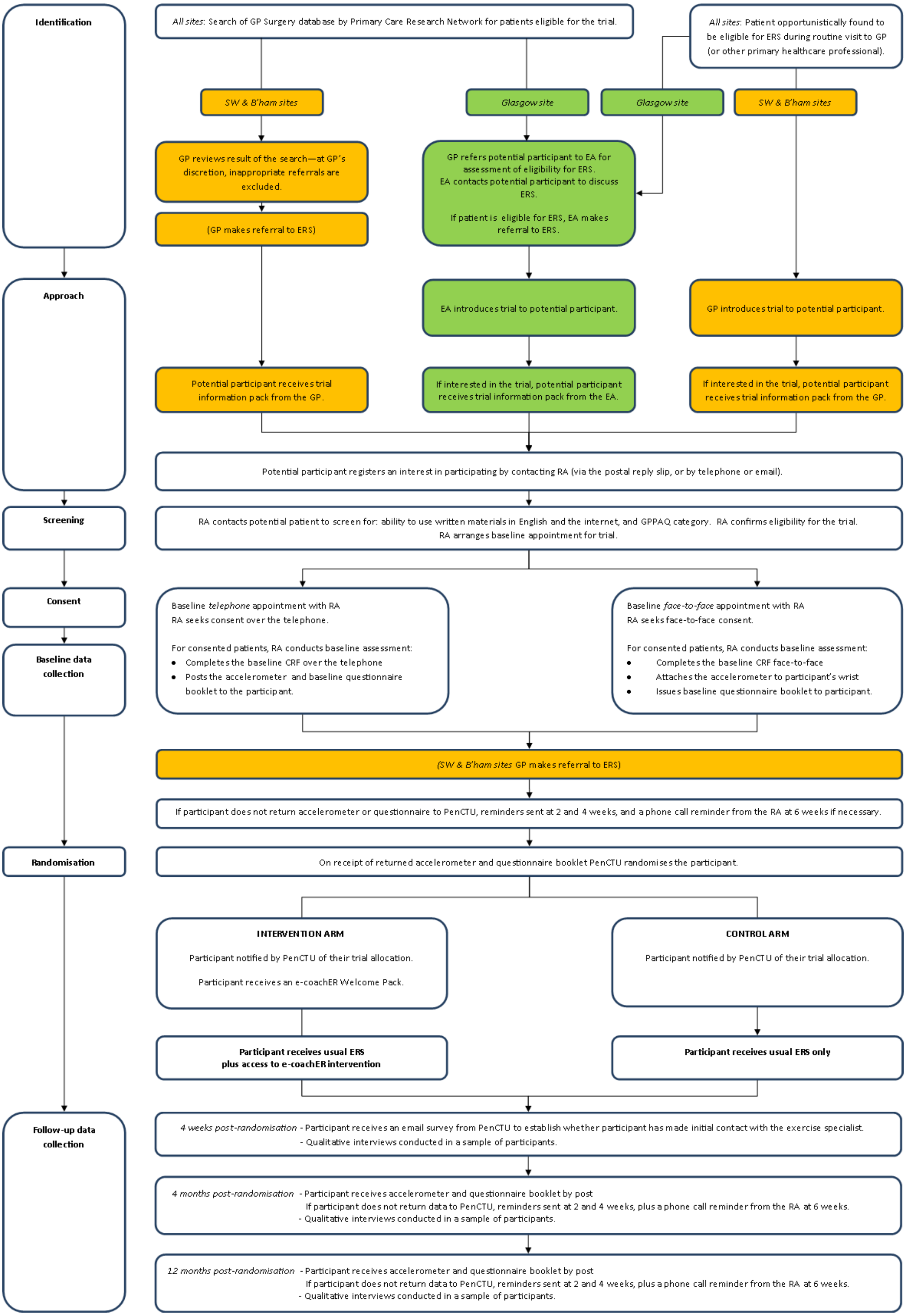

e-coachER Protocol_Version 6.1 20.11.2017 IRAS No. 170179 REC: 15/NW/0347 ISRCTN15644451 E001 Page 10 of 39

STUDY FLOW CHART

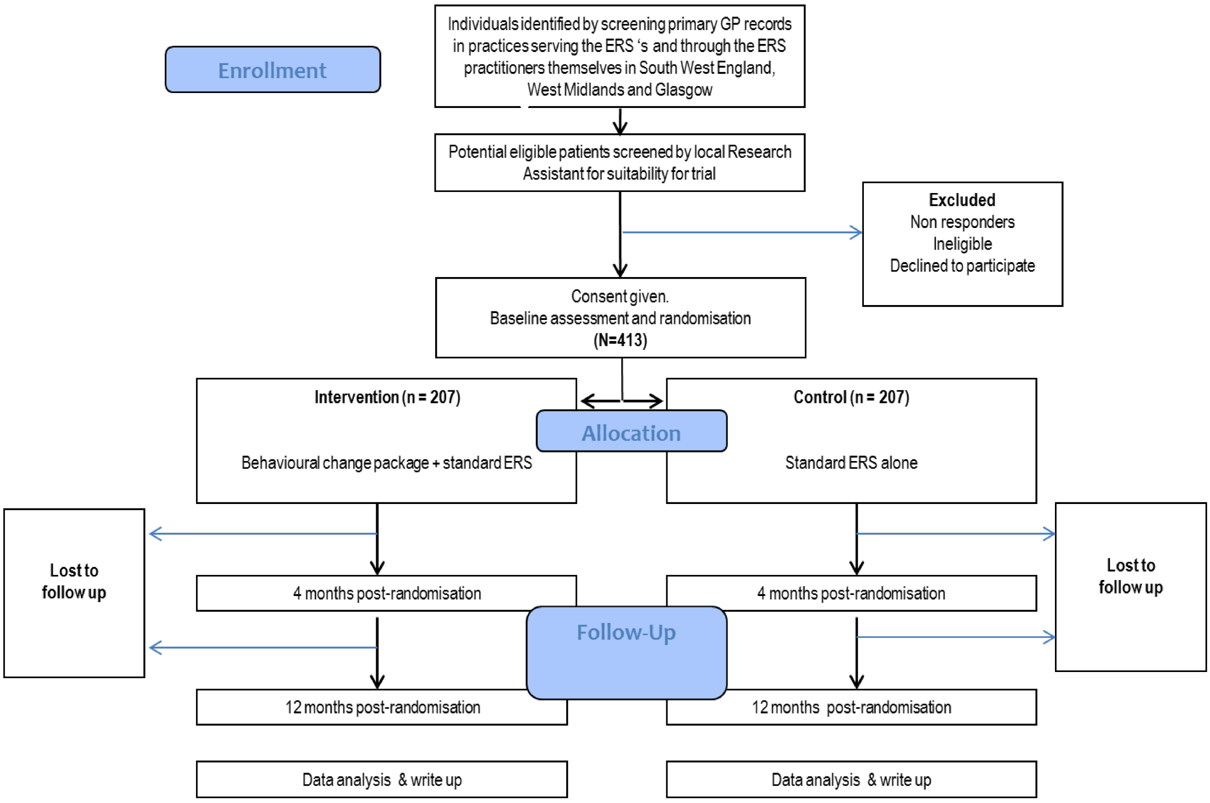

STUDY PROTOCOL

A multi-centre, randomised, controlled trial of an augmented exercise referral scheme (ERS) using web-based behavioural support in individuals with metabolic, musculo-skeletal and mental health conditions.

KEY WORDS

Randomised controlled trial; exercise referral scheme, web-based behavioural support.

1 BACKGROUND & RATIONALE

Metabolic, musculo-skeletal and mental health conditions place a major and increasing burden on health care resources, workplace sickness and absenteeism, as well as on individuals. Health problems associated with being overweight or obese, for example, cost the NHS more than £5 billion every year. There may be an increase from 2.6 million to > 4 million people with diabetes in the UK by 2025 as a result of more routine health checks. Hypertension and diabetes significantly contribute to premature mortality and morbidity related to cardiovascular disease, stroke and other serious illness.

Over one million adults each year consult their general practitioner with osteoarthritis and related conditions and this is expected to rise with increasing obesity. Depression is one of the most common reasons for consulting a general practitioner within the UK, and the associated economic burden is considerable and expected to worsen. Low mood and depression are common co-morbidities with metabolic and musculo-skeletal conditions.

The role of exercise

Across the UK the associated costs of inactivity are estimated at £1billion - £1.8billion (DH, 2011). Evidence-based guidelines (e.g. DH, 2011) recommend both aerobic and resistance exercise training for improving health markers and quality of life among those with common chronic metabolic conditions (i.e. obesity – NICE, 2010; hypertension – NICE, 2011; type 2 diabetes - NICE, 2008a) and musculo-skeletal conditions (e.g. osteoarthritis– NICE, 2008b), and mostly aerobic exercise for preventing and reducing depression (NICE, 2009). Significant health benefits and reduced health care costs could be gained with even a 10% increase in the proportion of the population, especially those with medical conditions, achieving the public health guidelines of at least 150 minutes of moderate to vigorous physical activity (MVPA) per week (DH, 2011).

The challenge of increasing physical activity

Patients with obesity, hypertension, type 2 diabetes, osteoarthritis and depression are less physically active than the general population (DH, 2011), and need greater support to overcome real and perceived barriers to increase physical activity (PA). Increases in PA amongst the least active have the potential to provide the largest impact on health but any benefits dissipate without maintained exercise (Dunstan, 2005). Since lower adherence, and lower exercise training volume and intensity, reduces health benefits, the challenge is to find appropriate ways to support sustained increases in aerobic and resistance exercise for those with or at risk of a medical condition.

A variety of initiatives have been explored to promote PA within primary care, including referring patients to 'exercise on prescription', i.e. exercise referral scheme (ERS). In the UK, ERS has been one of the most widespread approaches to promoting PA, with an estimated 600 schemes (involving up to 100,000 patients per year) linked to over 90% of primary care organisations (BHF, 2010).

Effectiveness of ERS

Evidence from a meta-analysis of robust trials on the effectiveness and cost-effectiveness of ERS (Pavey et al, 2011a) indicates a small increase in the proportion of participants who achieved 90-150 minutes of PA of at least moderate intensity per week, compared to control at 6-12 month follow-up

among at risk individuals. But uncertainty remains in the effects for patients with specific medical conditions since no study assessed long-term PA objectively.

Factors influencing effectiveness

In a systematic review (Pavey et al, 2012) pooled ERS uptake (attendance at the first exercise referral session) ranged from 66% in observational studies to 81% in randomised controlled trials, and adherence from 49% in observational studies to 43% in randomised controlled trials.

Predictors of uptake and adherence have rarely been explored but Pavey and colleagues (2012) reported that whilst women were more likely to begin an ERS, they were less likely to adhere to it than men, and also older people were more likely to begin and adhere to an ERS. ERS may help patients become familiar with concepts such as exercise type, intensity, frequency and duration of exercise, matched to their medical condition, and target key processes of behaviour change. However, the following features of an ERS may reduce uptake and adherence (BHF, 2010): inconvenience, cost, limited sustainable PA support (e.g. for 10 weeks), and low appeal for structured exercise and/or the medical model, i.e. 'exercise on prescription', which does little to provide autonomous support nor empower patients to develop self-determined behaviour to manage chronic medical conditions (Rouse et al, 2011).

Development of the trial intervention (e-coachER)

The LifeGuide platform has been extensively used to develop and evaluate acceptability and impact of behaviour change and self-management interventions with a variety of clinical groups, including in primary care (Lloyd et al, 2013; Williams, 2013; Yardley, 2010; 2011). It provides a researcher-led tool to develop interventions drawn from theory and evidence of effective techniques (Greaves 2011; Michie et al, 2009).

The proposed research therefore seeks to examine if web-based support using the LifeGuide platform (www.lifeguideonline.org/), to be referred to in this study as e-coachER, can be coherently combined with usual ERS to provide an effective and cost-effective approach to producing a sustained increase in PA. Both technologies involve relatively low cost (Anokye et al, 2011; Benaissa, 2012), and the proposed intervention has the potential to be rolled out across the UK. The UK prevalence of patients with obesity, hypertension, type 2 diabetes, OA and risk of depression is high and patients with these conditions are routinely referred to ERS (BHF, 2010). Should the approach prove to be effective there is considerable potential for patients with other chronic medical conditions (e.g. low back pain, heart disease), to be referred for exercise in more specialist services with e-coachER support.

A review of web-based public health interventions concluded that adding some human contact results in better long-term outcomes in mood (Newman et al, 2011). LifeGuide-based interventions combined with some human support have provided effective support for patients to self-manage various health behaviours over an extended period, including weight management, and will be used for the first time in this trial to support patients concurrently attending an ERS.

E-coachER was developed between July 2014 and January 2015, predominantly by researchers at the University of Southampton and Plymouth, and with input from PPI for beta testing and pre-piloting the intervention. A Welcome Pack is initially given to participants in the intervention arm, to include a User Guide, pedometer and fridge magnet with recording strips for monitoring daily physical activity steps and minutes of moderate intensity physical activity. Contact details are provided for support from a facilitator to assist with IT issues if required.

Once users have registered and logged on, e-coachER comprises seven short 'steps to health' which aim to increase uptake of the ERS support and the cognitive and behavioural skills to remain physically active. It is interactive in allowing users to record the amount of physical activity achieved, set and review weekly goals, and receive feedback. Throughout, there are short stories about how

others have used the support and overcome barriers. There are also links to carefully vetted websites (e.g. NHS, charities) on exercise and health, other local physical activity opportunities, and ways to use tracking software to monitor a range of health outcomes and behaviours.

Summary

For patients with chronic medical conditions, additional support from an exercise practitioner may be necessary to help them overcome initial and on-going barriers to maintaining a more physically active lifestyle, but it is unclear if current ERS schemes alone can provide this support. Traditional ERS may also create barriers for some patients but have the potential to provide valuable personal support and the opportunity to overcome barriers. We hypothesise that the additional support provided by e-coachER will improve the level of access to initial ERS support, improve the level of motivational support, and improve adherence to the ERS over a longer period of time than usual ERS, and thereby result in improved levels of sustained PA.

1.1 CHANGE OF PRIMARY OUTCOME MEASURE (SUBSTANTIAL AMENDMENT 04 dated 07 SEPTEMBER 2016)

1.1.1 Original trial design

The original design of the trial was a multicentre, parallel group, randomised controlled trial with an internal pilot. The primary outcome was the achievement of at least 150 minutes of MVPA measured objectively by accelerometer over one week at twelve months. The internal pilot phase was scheduled to run between July 2015 and October 2015 during which time 180 patients were to be recruited to provide sufficient information to justify progression to a main trial. For the main trial, a further 1220 patients were to be recruited, making a total of 1400 participants (Figure 1).

Progression from the internal pilot to the main trial was dependent on recruitment rate and engagement with the intervention according to the following scenarios:

Criteria	Scenario 3	Scenario 2	Scenario 1
% of internal pilot sample size target (180 patients) recruited.	< 65%	65 - 79%	≥ 80%
Intervention engagement (% who access e-coachER at least once)	< 65%	65 - 79%	≥ 80%
**Proposed Action**	No progression	Discuss with TSC and funder about progression and resources needed to achieve target.	Proceed to full trial.

Qualitative interviews with eligible non-participants, and participants not initially engaging with the intervention were to be conducted to inform the discussion about progression and ways to improve recruitment and engagement. There was no set progression target for recruiting a fixed proportion of patients with each of the six clinical conditions of interest since numbers were likely to be small across the three sites after only three months.

Figure 1: Original study flowchart

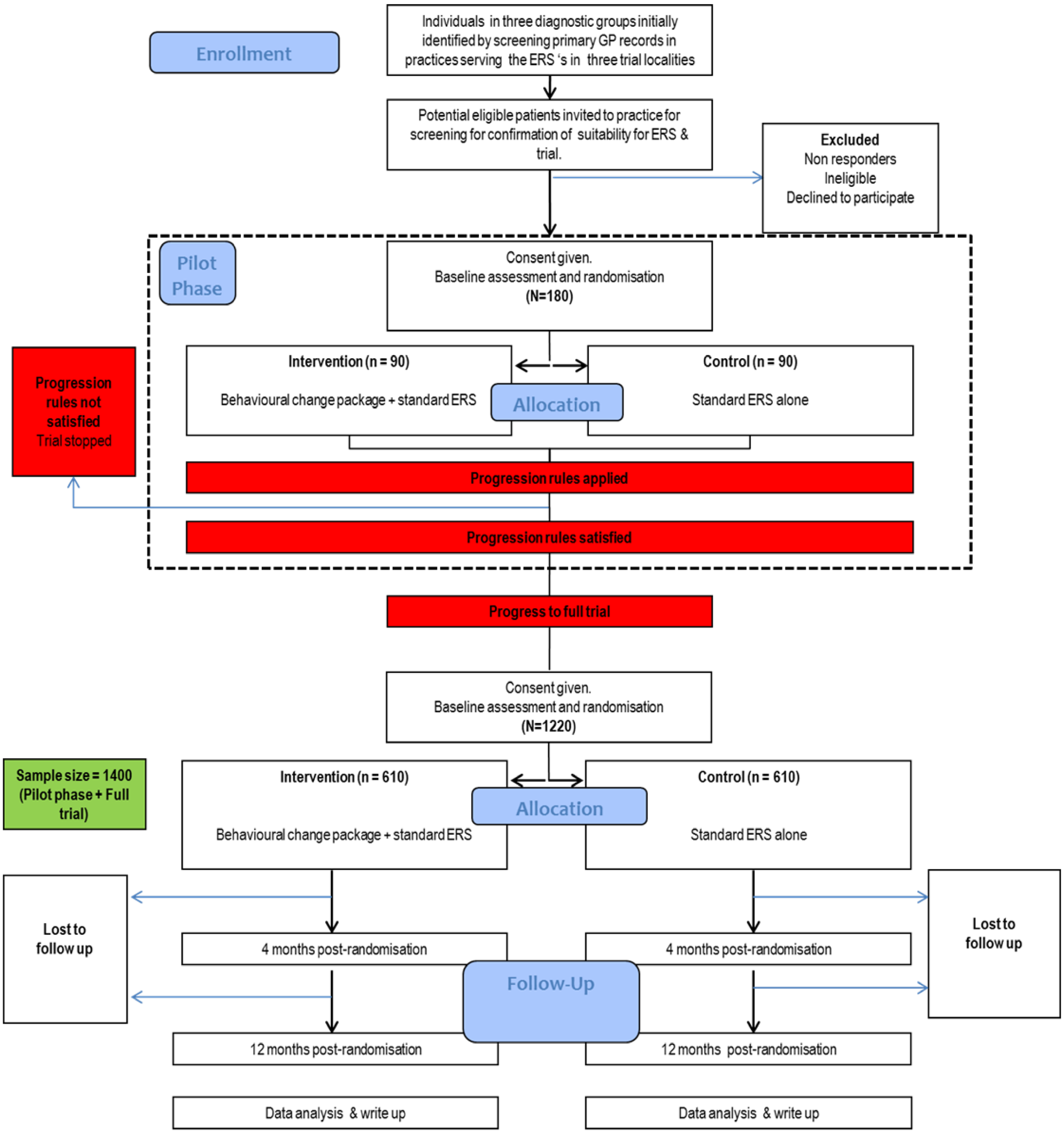

The conditions for progression were not met by the end of the internal pilot phase. At this point, on advice from the TSC, DMC and funders, the pilot phase was extended to the end of January 2016 to allow time for recruitment to be evaluated at one site that had not yet commenced recruitment (Glasgow), and to allow for a number of proposed strategies to increase recruitment to be implemented.

At the end of this extension period, recruitment at the Glasgow site had begun but there was no firm evidence that the original recruitment target could be achieved. Based on recommendations from the TSC and DMC, and in light of the research team's own updated literature review, a revised sample size was calculated using a continuous outcome (in contrast to the dichotomous outcome originally

proposed) and presented to the funder. The funders invited the submission of a detailed recovery plan.

1.1.2 Recovery Plan: Proposal

The recovery plan comprised:

1. Change the primary outcome measure to a continuous variable (i.e., total weekly MVPA minutes recorded by accelerometer in ≥10 minute bouts) at 12 month follow-up, resulting in a reduced sample size, from 1400 originally to 332 participants (for an ES of 0.4) or to 413 (ES of 0.35), allowing for 20% attrition. It was estimated that a sample size of 562 participants would be needed with an ES of 0.3, and this was felt to be unachievable with the available funding.
2. Continue recruitment activity for a short time to confirm that recruitment according to the revised target, across all three sites, could be achieved.

1.1.3 Recovery Plan: Scientific rationale and justification for reducing the sample size

Original sample size calculation

This sample size was based on the previous HTA systematic review of ERS (Pavey et al 2011a & 2011b) that showed that trials up to that time had primarily reported their outcomes according to percentage of participants reaching the threshold of 150 minutes of MVPA per week. Using this binary outcome, it was estimated that recruiting 700 participants per group in the e-coachER trial would able us to detect a difference at 12-months follow up of at least 10% (intervention group: 53% vs. control group: 43%) and assuming an attrition rate of 20% and small effect of clustering (ICC: 0.006) at 90% power and 5% alpha. The exploratory modelling indicated a change of ≥10% is required for the intervention to achieve an incremental cost effectiveness ratio of <£20,000/ QALY.

Revised sample size proposal

The required sample size was recalculated considering the difference between groups in MVPA in minutes i.e. considering the primary outcome as continuous. In absence of a published minimally important difference for MVPA, assuming a 'small' to 'moderate' standardised effect size of 0.4, it was estimated that 132 participants per group at 90% power and 2-sided alpha of 5% were required (using 'sampsi' in STATAv.14). Allowing for a 20% attrition rate, a total of 332 participants would need to be recruited.

Following presentation of this revised sample size proposal and discussion with the funder, it was agreed that the trial sample size be revised and be based on a standardised effect size of 0.35 and a total of 413 participants recruited. Assuming an effect size of 0.35, provides 88% power at a 2-sided alpha of 5% assuming 20% attrition or 90% power at a 2-sided alpha of 5% assuming 16% attrition.

Given that the e-coachER intervention is being delivered at the level of the individual participant, a clustering effect has not been factored into this revised sample size calculation. Based on the baseline standard deviation for MVPA total weekly minutes in ≥ 10 minute bouts of 104 to 113 reported by Harris and colleagues (Harris, 2015), an effect size of 0.35 would correspond to a between group difference of 36 to 39 minutes of MVPA/week.

While international reviews and guidance have clearly identified the importance of PA for preventing and treating patients with the chronic conditions that we are recruiting in the e-coachER trial, it is less clear precisely how much change in physical activity would contribute to a minimally important clinical

difference (across all our target clinical groups). Public health guidelines of 150 minutes of MVPA per week are widely accepted but even small increases in PA and reduced sedentary time among the least active are likely to accrue health benefits (Bouchard et al, 2015; Warburton et al, 2016), and be cost-effective, especially for a low-cost web-based intervention. But detecting small differences (compared with a control group) usually requires very large sample sizes which are beyond the scope of research funding. We will be able to apply the trial data of a change in MVPA in minutes to existing and emerging cost-effectiveness models; a paper by Anokye is under review, and others have done this (e.g., Larsen, 2015).

Following our previous systematic review of ERS and since the approval of funding of the e-coachER trial we continue to monitor relevant literature on the effectiveness and cost-effectiveness of ERS, and there have been no further systematic reviews or original studies of relevance to the ERS literature. However, interest in web-based interventions to promote physical activity has continued to grow.

Several systematic reviews have been identified (e.g. Joseph *et al*, 2014 – 72 studies; Devi *et al*, 2015 – 8 studies), and at least 15 original studies that have reported on the effects of technology-based interventions on PA since 2013. The reviews have included studies with a wide range of interventions (from quite simple self-monitoring to ones with complex multiple behaviour change components), targeted at different clinical groups with different baseline levels of physical activity, with various physical activity outcomes reported (very few using objective measures), and with mostly short-term follow-ups. Also, some comparisons are with no intervention and others are with human contact, though none report on the effects of adding web-based support to ERS. This makes their relevance to assessing the effectiveness and cost-effectiveness of our e-coachER intervention limited or unclear. But some general findings are important; the overall effect size for web-based and technology interventions is small to moderate (up to 0.4), but there is evidence that more rigorous studies, interventions with more behaviour change components, and ones targeted at less active populations are more effective. Given that an effect size of 0.4 would be equivalent to approximately 42-45 minutes of MVPA per week, we searched for individual studies reporting such a between group difference at follow-up to identify the study characteristics and similarities to e-coachER. We also noted the sample size justification for each study that included minutes of MVPA as a continuous outcome.

Of 10 individual studies (involving likely comparable participants to those in e-coachER) reporting outcomes from a comparison of web-based intervention versus control, since 2013, 4 reported accelerometer assessed physical activity. Including 2 further studies with a published protocol, the estimated sample sizes required to detect significant between group differences in continuous physical activity outcomes was 48 to 397.

In a study with a total of 94 participants with angina, Devi *et al* (2014) reported at 6 months the following effect sizes in favour of a web-based intervention, compared with usual care, for daily steps (effect size =0.24, 95% CI:–358 to 2324, P=0.15), daily energy expenditure (0.38, 95% CI: –35.17 to 250.47, P=0.14), duration of sedentary activity (0.55, 95% CI: 0.190 to -0.205, P=0.20), duration of moderate activity (0.55, 95% CI: 0.244 to -0.261, P=0.24), recorded by accelerometer.

In a study with a total of 300 participants, Harris *et al* (2015) reported at 12 months a between group difference in favour of the digital intervention (pedometer monitoring and reflection, in primary care) of 609 steps/day (95% CI: 104 to 1,115, p = 0.018) and 40 minutes/week MVPA (95% CI: 17 to 63, p = 0.001).

In a study with a total of 179 participants at 3 months follow-up, Compernolle *et al* (2015) reported a net difference in daily step counts (recorded by a user-blinded pedometer) of 895, and 12% difference

in the proportion achieving the recommended 10,000 steps per day, in favour of the intervention compared with a control. This study included both pedometer and web-based support like e-coachER.

Finally, the only study (Wijsman *et al*, 2013) we have found which used the same GeneActive accelerometer used in e-coachER reported that a web-based intervention, offered to half the 226 participants, led to a mean increase of 11.1 minutes per day spent in MVPA, compared to a mean decrease of 0.1 minutes in the control group (P =0.001) at 3 months.

Systematic reviews (e.g. Davies *et al*, 2012) have also highlighted the importance of maximising sustained engagement in web-based interventions for enhancing change in the target behaviour. Recent studies (e.g. Morrison *et al*, 2014) confirmed that self-assessment and tailored feedback were important to increase engagement, and periodic communications help to maintain participant engagement. The e-coachER trial links closely to another LifeGuide delivered intervention (for weight loss) called POWeR, in which a combination of face to face and web-based support led to the greatest weight loss (Yardley et al, 2014); those completing at least 9 of the 12 recommended brief sessions lost 6.7kg, whereas those who did not, lost 1.5kg at 12 months. Our intervention also provides ERS practitioner support in addition to e-coachER web-support. We also seek to maximise engagement with e-coachER support, with follow-up automated e-mails for 12 months. Based on the first 60 participants allocated to the e-coachER intervention over 65% have accessed the on-line support system, and we continue to monitor that through our process evaluation.

1.1.4 Recovery Plan: Outcome

The funder accepted the recovery plan, stipulating the following conditions:

1. Change the primary outcome measure to a continuous variable, as proposed.
2. Continue recruitment and achieve a sample size of 413 by the end of March 2017 (i.e. a 5 month extension to recruitment).

1.1.5 Summary

- The primary outcome has been changed to a continuous variable, i.e. total weekly minutes of MVPA in ≥10 minute bouts, recorded objectively by accelerometer, over one week at twelve months.
- As a result of changing the primary outcome from a dichotomous to a continuous variable, the sample size has been reduced from a total of 1400 participants to 413 participants **(206** per group) based on detecting a between group effect size of 0.35, allowing for 20% attrition, with 5% significance and 88% power.
- The recruitment window will be increased from 15 to 20 months.

2 OBJECTIVES AND OUTCOME MEASURES

To determine whether the addition of a web-based support package to usual ERS increases the minutes of moderate to vigorous intensity physical activity (MVPA) at twelve months, compared with ERS alone, and whether such an intervention is cost-effective.

2.1 Objectives

The objectives are as follows:

- To determine whether in the intervention participants compared to the controls, there is an increase in the total weekly minutes of MVPA at twelve months post-randomisation.
- To determine whether in the intervention participants compared to controls there is an increase in the proportion of participants who:
    - Take up the opportunity to attend an initial consultation with an exercise practitioner
    - Maintain objectively assessed physical activity at four and twelve months post-randomisation
    - Maintain self-reported physical activity at four and twelve months post-randomisation
    - Have improved health-related quality of life at four and twelve months post-randomisation
- To quantify the additional costs of delivering the intervention and determine the differences in health utilisation and costs between the intervention and control arms at twelve months post-randomisation.
- To assess the cost-effectiveness of the intervention compared with control at twelve months post randomisation (incremental cost per QALY) and over the lifetime perspective (incremental cost per QALY) using a previously developed decision model to estimate future costs and benefits.
- To quantitatively and qualitatively explore whether the impact of the intervention is moderated by medical condition, age, gender and socioeconomic status, or ERS characteristics, IT literacy.
- To quantitatively and qualitatively explore the mechanisms through which the intervention may impact on the outcomes, through rigorous process evaluation and mediation analyses.

All primary and secondary outcomes will be collected on both intervention and control arm participants unless otherwise indicated below.

2.2 Primary outcome

The primary outcome is the achievement of more weekly minutes of MVPA, in ≥10 minute bouts, recorded objectively by accelerometer, over one week at twelve months compared with the control group.

Taylor A, *et al. Br J Sports Med* 2021; 55:444–450. doi: 10.1136/bjsports-2020-103121

2.3 Secondary outcomes

Secondary outcomes are:

- Total weekly minutes of MVPA in ≥10 minute bouts, recorded objectively by accelerometer, over one week at four months.
- Achievement of at least 150 minutes of MVPA, measured objectively by accelerometer, over one week at four and twelve months post-randomisation.
- Self-reported achievement of at least 150 minutes of MVPA over one week using the Seven Day Physical Activity Recall Questionnaire at four and twelve months post randomisation.
- Self-reported weekly minutes of MVPA at four and 12 months.
- Self-reported health-related quality of life, assessed by the EQ-5D-5L and SF12v2 at four and twelve months post randomisation.
- Self-reported symptoms of anxiety and depression, assessed by the Hospital Anxiety and Depression Scale (HADS) at four and twelve months post randomisation.
- Average daily hours/minutes of sleep and sedentary behaviour (objectively measured by accelerometer) over one week at four and twelve months post randomisation.
- Uptake of the ERS by participant self-report at approximately four weeks and four months post randomisation.
- Adherence to the ERS, using a composite measure to describe the proportion in each arm of the trial that achieved the primary outcome at four months and were still doing so at twelve months.
- Process measures, to be described and included in mediation analysis including 1-4 self-reported survey items for each of the following: self-efficacy/confidence to be physically active; importance of being physically active; relatedness (perceived frequency and availability of support); perceived autonomy/control over physically active choices; involvement in self-monitoring and planning PA.
- In the intervention group, measures of engagement with e-coachER, and its content, and use of self-monitoring and goal-setting functions, captured by the software platform (LifeGuide).
- Qualitative interviews with participants in the intervention arm, focusing on their experiences with ERS and the intervention. Also, interviews with eligible participants who decline to enter the study to assess acceptability of trial methods.

2.3.1 Economic outcomes

The costs associated with the following will be determined:

- Development of the intervention to include the ‘Welcome Pack’, with a view to costing the (potential) roll-out of the intervention to a wider population.
- Self-reported monetary costs of health service use, use of the ERS and use of the web-based support package, at four and twelve months.
- Costs of support (including training) provided by the e-coachER facilitator (RA) and LifeGuide technician.
- Health and personal social care use (self-reported at four and twelve months).
- Personal costs for participation in PA (including use of ERS) at four and twelve months.

The main outcome of the economic analysis will be the incremental cost per Quality-Adjusted Life-Year (QALY) at twelve months, based on EQ-5D-5L.

3 TRIAL DESIGN

The design is a multicentre, parallel group, randomised controlled trial. Patients will be individually randomised to receive usual ERS alone (control) or usual ERS plus access to a web-based support package (e-coachER), and motivational and technical support (intervention). The trial will have parallel economic and process evaluations.

In the set-up phase the research team, and ERS associates will adapt and test e-coachER. The Welcome Pack and platform will be tested with ERS patients and final adaptations made in response to users' feedback.

Thereafter, 413 patients will be recruited to determine the effectiveness and cost-effectiveness of the addition of the intervention to ERS, relative to usual ERS alone.

4 STUDY SETTING

The study is a multicentre study with three participating sites – South West (Devon and Cornwall), Birmingham, and Glasgow, where exercise referral schemes currently exist. All participants will be referred by a GP or health professional working in primary care to a local exercise referral scheme in the community. Those participants randomised to receive the intervention will be given access to the e-coachER support package.

5 ELIGIBILITY CRITERIA

5.1 Inclusion criteria

Patients must satisfy the following criteria to be enrolled on the study:

- Aged 16-74 years
- Have one or more of the following:
  - Obesity (BMI30-40)
  - Diagnosis of hypertension
  - Type 2 diabetes
  - Prediabetes ('borderline diabetes')
  - Lower limb osteoarthritis
  - Recent history of treatment for depression (i.e. last two years) but may not be currently receiving treatment
- Categorised as 'Moderately Inactive' or 'Inactive' according to the physical activity index calculated from the GP Physical Activity Questionnaire.
- Be contactable by e-mail and have at least some experience of using the internet.

5.2 Exclusion criteria

Patients who meet any of the following criteria will be excluded from study participation:

- Unstable, severe and enduring mental health problem that may limit involvement in the trial.
- Being treated for an alcohol problem or drug addiction that may limit involvement in the trial.
- Inability to use written materials in English, unless they have access to a readily available designated friend or family member to translate.
- Does not meet the inclusion criteia for a referral to the ERS, e.g. has a medical condition that is contra-indicated for the ERS.

6 RECRUITMENT

Eligible participants will be patients with the chronic conditions of diabetes, prediabetes, obesity, hypertension, osteoarthritis or a history of depression who are suitable for referral to a local exercise referral scheme from a health professional working in primary care.

6.1 Patient identification and approach

Patients will be recruited in more than one way since the usual care pathway varies between sites and participating GP practices. At participating GP practices, patients being actively referred to an ERS or opportunistically found to be eligible for an ERS (e.g. during a routine NHS health check or visit to a surgery) may be identified by the GP/ practice research nurse / PCRN research associate / other health professional as being potentially eligible for the study. In addition, the GP database will be searched by practice staff or PCRN research associate, for patients who are potentially eligible for an ERS, and such patients invited for an appointment with the GP / practice research nurse / PCRN research associate/ Research Assistant to establish eligibility for ERS. Referral to the ERS will be made by a member of the primary care team.

At some sites, potential participants will also be identified by exercise advisors from patients referred by the GP for assessment of suitability for the ERS.

6.2 Approach/invitation to participate

Depending on the identification route and local care pathway, a member of the GP practice team or the exercise advisor will provide potential participants with a trial Information Pack (by post or by hand). Alternatively, potential participants may be given a summary study information sheet containing contact details for the local RA who will send an Information Pack directly to the patient once contact has been made by the patient.

The Information Pack comprises an outer envelope displaying brief information about the trial containing an invitation letter, Participant Information Sheet and reply slip. Patients will be asked to indicate on the reply slip if they are interested in participating in the trial, and to return the reply slip to the local RA in the Freepost envelope provided. Patients may also contact the relevant site research team via a dedicated answer phone at each site or by e-mail.

In addition, interested patients will be asked by the exercise advisor if they are willing for their contact details to be passed on by the ERS service to the local RA, and if so, the local RA will make contact with the patient as described in Section 6.3.

6.3 Screening and consent

On receipt of a completed reply slip (or equivalent expression of interest), a member of the local research team will contact the potential participant to outline the study, answer any queries and establish eligibility for the trial.

If the patient is interested in taking part in the trial and appears eligible, the research team member will offer to arrange a face-to face meeting with the patient to complete the consent process, provide the wrist-worn GENEActiv accelerometer and baseline questionnaire. Alternatively, the consent process can be completed during this same telephone call and the researcher can post the accelerometer and baseline questionnaire to the patient.

6.3.1 Face to face consent process

The face-to-face screening/consent appointment will usually take place at the location of a primary healthcare provider (which will usually be the GP practice), or at the location of the ERS provider

Taylor A, *et al. Br J Sports Med* 2021; 55:444–450. doi: 10.1136/bjsports-2020-103121

(which is usually a leisure centre). Other locations may also be used to maximise convenience for participants and availability of quiet and secure office space, such as in pharmacies, and academic centres and at peoples' homes.

At this session, the research team member will describe the study, answer any questions the patient may have and check final eligibility for the ERS and trial, including the General Practice Physical Activity Questionnaire (GPPAQ). Patients who are willing and eligible to take part will be asked to complete, sign and date the study consent form, which will also be signed and dated by the person obtaining consent. A copy of the signed consent form will be given to the participant and the original signed form will be retained in the Investigator Site File.

6.3.2 Telephone consent process

If the patient is unable or unwilling to meet with the researcher in person, consent can be obtained via the telephone. Patients will be provided with the same information as in the face to face process (above) and given the opportunity to have any questions answered. Inclusion/exclusion criteria, including the GPPAQ, will be checked. If patients are willing and eligible to take part, the researcher will read out the separate elements of the consent form and get the patient's verbal assent for each one. The researcher should initial each box on the consent form to indicate that each clause has been read to and agreed by the patient. The researcher should sign and date the consent form. A copy of the researcher-only signed consent form will be sent to the participant and the original researcher-only signed form will be retained in the Investigator Site File. Given the nature of the study, there is no requirement for participants to sign the consent form themselves in the case of telephone consent.

6.4 Planned recruitment rate

The recruitment target is 413 participants (138 participants per site). The following strategies to maximise recruitment will be used as necessary:

- Encourage practices to maintain or increase routine identification and referral of patients into local ERS's.
- Engage with GP practices and/or exercise advisors at the ERS's to identify eligible patients.
- Raise patient awareness of the study at GP practices and ERS's (e.g. presentations, posters, website) to foster opportunistic interest.
- Site PI's and RAs to work closely with the local Research Network, to identify practices for recruitment in a timely manner.
- Utilise the site research assistant (RA) to maintain a proactive approach to recruitment and monitor ERS waiting times (referral throughput) to ensure the recruitment rate approximately matches the ERS capacity.

6.4.1 Addressing trial and intervention 'reach'

There is a risk of recruiting a higher proportion of patients who tend to be more physically active (and hence with less to gain from the intervention), and only those familiar with web-based and mobile technologies. In order to recruit less active patients and those with only limited familiarity with internet and mobile technologies the following approaches have been and will continue to be used:

- Conduct focus groups and individual interviews with patients and practitioners with relevant experience to determine how best to describe the study and intervention in recruitment and intervention (e.g. Welcome Pack) materials.
- Work with local authority and third sector organisations to identify local opportunities to ensure that appropriate IT support can be described in trial materials and provided to participants receiving e-coachER.

- Identify specific roles for the e-coachER RA to support patients' use of the technology.
- Continue to monitor local and academic reports on optimising the use of e-coachER for those with low IT use (e.g. older people, disadvantaged populations).

7 BASELINE DATA COLLECTION

Baseline data collection includes demographic data, a simple IT literacy question the baseline questionnaire booklet and baseline accelerometry data. Demographic data will be collected by direct questioning at the time of consent and recorded in the case report form (CRF).

Participants attending a face to face screening/consent visit will complete the baseline questionnaire booklet at this visit, following consent. Each participant will also be provided with a GENEActiv accelerometer. The researcher conducting the face-to-face screening appointment will attach the accelerometer to the participant's non-dominant wrist. The participant will be asked to wear the accelerometer for the next seven days and to return it to the Peninsula CTU after that time, in the pre-paid envelope supplied. The researcher will send the complete baseline questionnaire booklet to the CTU.

For participants consenting to the study by telephone, the local researcher will post a copy of the researcher-signed consent form, baseline questionnaire booklet, accelerometer, instructions for use and a pre-paid return envelope to the participant following verbal consent. The completed questionnaire booklet and used accelerometer will be returned directly to the CTU by the participant.

The CTU will send a standard letter to participants three days after the accelerometer has been administered by post, as a prompt to the participant to begin wearing the accelerometer, if not already doing so.

The CTU will send up to two reminder letters (at 2 and 4 weeks) and/or make two telephone calls) to participants to prompt the return of both accelerometers and baseline questionnaire booklets. If the participant has not returned the accelerometer after 6 weeks the local Research Assistant will remind the participant via the telephone. Participants who return the accelerometer to the CTU will receive a high street/online store voucher of £20 as a 'thank you' payment.

8 RANDOMISATION

Following receipt of the baseline survey and accelerometer, randomisation will be carried out by the PenCTU. Randomisation will be conducted by means of a secure, password protected web-based system created and managed by the CTU in conjunction with the trial statistician. Participants will be randomised to usual ERS or usual ERS plus access to e-coachER in a 1:1 ratio, stratified by site (1=SW; 2=Birmingham; 3=Glasgow) with minimisation by patient's perception of main medical referral reason (1=control diabetes; 2=weight loss; 3=lower blood pressure; 4=manage lower limb osteoarthritis symptoms; 5=manage mood/depression), IT literacy level (1=lower confidence; 2=higher confidence). To maintain concealment, the minimisation algorithm will retain a stochastic element.

CTU will inform the participant of the treatment allocation by standard letter. Participants allocated to the intervention arm will also be sent an e-coachER Welcome Pack (see section 7).

Blinding of trial participants is not possible, given the nature of the intervention. Given that the primary outcome is an objective measure of physical activity recorded by the wrist-worn accelerometer and the secondary outcomes will be assessed by participant questionnaire self-completion, the risk of assessor bias is likely to be negligible in this study. However, to minimise any potential bias, the statistical analysis will be kept blinded and the code for group allocation not broken until the primary and secondary analyses have been completed.

9 TRIAL INTERVENTION

The e-coachER intervention is an engaging support package to help people on an ERS to become and remain more physically active. The intervention consists of an interactive website plus a pedometer and a fridge magnet with paper strips for recording the number of daily activity steps and minutes of moderate intensity physical activity. Without engagement, the intervention can have no additional benefit. The first point of contact with the intervention is therefore a user-friendly Welcome Pack. Figure 2 shows the version to be given out at face-to-face opportunities; a non-boxed version will be used for mailing to participants.

The Welcome Pack contains a User Guide with a unique User ID to enable participants to register and log into the e-coachER website easily. It also includes a good quality pedometer and the fridge magnet with attached record sheets. Contact details for further IT support are also provided. The User Guide shows screenshots of pages in the e-coachER website, including the seven 'Steps to Health'.

Figure 2: The Welcome Pack

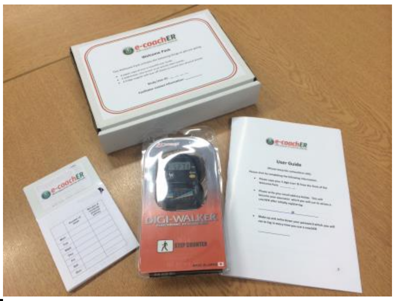

E-coachER aims to increase uptake of support offered by exercise practitioners at the ERS, but also provides a stand-alone interactive website to facilitate skill development to remain physically active.

The support provided by the e-coachER website is autonomous in that participants set their own (hopefully progressive) targets and choose their preferred types of activities. Appendix 1 shows each element of the e-coachER support package, the objective of each element, the behaviour change technique used to achieve each objective, and the strategy for implementing each behaviour change technique. The Research Assistant at each site will provide general and local motivational IT support and the LifeGuide technician will support minor operational issues across all sites.

10 TRIAL ACTIVITIES AND FOLLOW-UP

The study schedule is given in Table 1.

10.1 Exercise Referral Scheme

Participants will attend the ERS according to local standard care, typically after completion of baseline assessments and randomisation to trial arm. Protocols for ERS's have been agreed at each site. These vary from the more traditional approach with patients receiving supervised exercise sessions by a qualified exercise practitioner 1-2 times per week to more office-based support and signposting to exercise in a variety of community settings.

10.2 Follow-up assessments

At four weeks post-randomisation, the CTU will email all participants a survey about ERS attendance. At four and twelve months post-randomisation, the CTU will post all participants an explanatory cover letter, an accelerometer (with an instruction sheet), self-completion questionnaire booklet, and a pre-paid envelope for return of the accelerometer and questionnaire booklet.

The CTU will send a standard letter to participants approximately 1 week prior to the 4 month and 12 month follow-up assessments, as notification that the items listed above will shortly be sent. Furthermore, the CTU will send a standard letter to participants three days after the accelerometer has been administered, as a prompt to the participant to begin wearing the accelerometer, if not already doing so.

The CTU will send up to two reminder letters to participants (supported by a telephone call or email as required) to prompt the return of both the accelerometer and questionnaire booklet. Participants who return the accelerometer to the CTU will receive a high street/online store voucher (£20 at four months and £20 at twelve months) as a ‘thank you’ for participating.

Table 1: Study schedule

Measure	Baseline	4 weeks	4 months	12 months
(IT needs assessment at screening)				
Demographics	X			
Medical condition for referral	X			
Accelerometer (worn for 1 week) - minutes of MVPA, sleep, and light activity per week	X		X	X
Sessions held with exercise practitioner (retrospective self-report) as an indicator of ERS engagement			X	X
Self-reported physical activity (7 day PA questionnaire)	X		X	X
Health & social care resource use	X		X	X
EQ-5D-5L, SF12v2	X		X	X
HADS	X		X	X
Process outcomes e.g. confidence, importance (1)	X		X	X
Qualitative interview (sample of participants)		X	X	X
Retrospective check of ERS attendance (by e-mail, questionnaire, and ERS attendance records)		X	X	X

(1) See full list in section 2.3: Secondary outcomes.

10.3 Retrospective check of ERS attendance by study team

To ascertain the uptake of and adherence to ERSs, the study team shall collate information on participants’ ERS attendance directly from the local ERS provider.

10.4 Qualitative assessments

Qualitative interviews will be conducted by a single e-coachER research assistant, as part of the process evaluation, based in Exeter. The main consent form for the study includes a statement that participants may be contacted for interview but that this part of the study is optional and participants do not have to agree to be interviewed. Upon contacting the participant by phone, the RA will explain the broad interview content, that the interview will be recorded, and processes to ensure the data remains confidential and anonymous during data analysis. Further verbal consent will be obtained, and a consent formed signed by the RA. Interviews will be conducted either face-to-face or over the telephone. All interviews will be transcribed with any personal data or ways of identifying participants being removed. Transcriptions will be coded, thematic analysis performed to identify key findings. The focus of the interviews will be linked to the phase of the research.

10.4.1 Feasibility and acceptability of the intervention and trial methods

(1) To inform our understanding of recruitment feasibility and acceptability, participants who are eligible but who decline to join the study will be asked to indicate by return of the reply slip if they are willing to be contacted to determine what influenced their decision not to join the study. Questions will broadly focus on the following: (a) understanding of what the study/intervention is about based on the Information Pack materials; (b) confidence (or lack of) in using the internet; (c) perceptions of available support to overcome IT issues; (d) beliefs about the value of a website in the context of ERS. We will seek to interview as many participants as possible at this stage.

(2) To inform our understanding of perceptions about engaging with the intervention, we will interview those who, within three weeks of being allocated to the on-line intervention group, (a) do not register on-line for e-coachER or (b) register but then never log in again; or (c) register and log in once, but don’t get beyond Step 1 and/or 2 (i.e. do not get involved in any of the core behaviour change techniques, including self-monitoring and goal setting). Questions will broadly focus on perceptions of the Welcome Pack, the process of registering on-line and accessing e-coachER, and the initial content and support provided. We will seek to interview as many participants as possible at this stage.

10.4.2 Functionality and utility to support behaviour change

Participants from the following groups will be interviewed (a) used e-coachER a few times then stopped, or never get beyond say Step 3 or 4; (b) got through all seven steps. We will select a random sample of about 40 participants but the precise number of interviews will be determined by data saturation and resources available.

The interview schedule will include questions about the value of the Welcome Pack and contents in helping to access e-coachER, the overall web-based support and each of the Steps to Health, in terms of functionality and utility to support behaviour change. Participants will be asked to identify if and how they thought e-coachER provided support in accessing an exercise practitioner within the ERS, and maintaining physical activity. Ideas for additions or revisions to e-coachER will be requested.

Questions about support for behaviour change will also attempt to provide qualitative information about some of the processes within our logic model and to be assessed quantitatively within the four and twelve month assessments. For example, questions will focus on changes in perceived importance of physical activity, support used and received to increase physical activity, perceived changes in competence, and autonomy of decisions concerning physical activity.

10.4.3 Interviews with e-coachER facilitators

E-coachER facilitators at each site will record the type and amount of support requested at an individual level, and provided in field notes. Interviews with e-coachER facilitators during and at the end of the trial will be conducted to identify strengths and weaknesses of their supporting role.

10.5 Withdrawal criteria

A participant may, at any time, withdraw from the study without giving a reason and without it affecting his/her clinical care. Participants will be asked to give a reason for withdrawal from the study but do not have to provide one. Participants who wish to withdraw will be given the option to continue with partial follow-up, e.g. provide primary outcome data only, to minimise data loss. Participants who withdraw from the study will not be replaced. The CTU data management team will ensure that participants who formally withdraw from the study are not contacted for any subsequent follow-up data collection (aside from any partial follow-up arrangements made with individual participants). Data collected prior to withdrawal will be included in the study analysis unless a participant specifically requests that their data are removed from the database.

10.6 End of trial

Participants will normally complete the study after returning the completed twelve month questionnaire booklet and used accelerometer. The trial itself will end on the date that the last participant completes the twelve month follow-up assessments.

11 SAFETY REPORTING

11.1 Definitions

Adverse event (AE)
Any untoward medical occurrence, unintended disease or injury or any untoward clinical signs in study participants whether or not related to any research procedures or to the intervention.

Serious Adverse Event (SAE)
A serious adverse event in the context of this study is any untoward medical occurrence that:

- Results in death
- Is immediately life-threatening
- Requires inpatient hospitalisation
- Results in persistent or significant disability/incapacity

11.2 Reporting requirements for this study

The recording and reporting of non-serious AEs in this study is **not** required. Information about SAEs may be captured in a variety of ways (see below). SAE report forms will be returned to the CTU and entered into the study database. The CTU will prepare quarterly summaries of SAEs, listed by organ system where possible, for review by the DMC and Sponsor.

11.2.2 In-patient data from questionnaires at 4 and 12 months

The resource use questions in the self-completion study questionnaire booklets ask participants to record the number of in-patient episodes within a set recall period. At the four and twelve month time points, participants are asked to record if they have been hospitalised, the reason for any hospital admission during the past four and eight months respectively and whether they think that the hospitalisation was related to participation in this study. On receipt of a questionnaire indicating a past hospital admission, the

CTU will liaise with the relevant local RA who will be responsible for ascertaining further details about the SAE from the participant and/or GP records as appropriate.

11.2.3 Notification of SAEs via GP

Once a patient is recruited to the study, the participant's GP will be notified by letter. The notification letter includes a request for the GP to contact the CTU in the event of the GP becoming aware of any SAE. On being informed of an SAE, the CTU will liaise with the relevant local RA who will be responsible for ascertaining further details about the SAE from the participant and/or GP records as appropriate.

11.2.4 Notification of SAEs from other sources

It is possible that the local research team or CTU may become aware of an SAE via patient or relative self-report or some other channel. In such cases, the local RA will be informed of the SAE in order to ascertain further details for reporting to the CTU.

12 STATISTICS AND DATA ANALYSIS

12.1 Sample size calculation

In absence of a published minimally important difference for MVPA, assuming a 'small' to 'moderate' standardised effect size of 0.35, it is estimated that 413 participants total is required at 88% power and a 2-sided alpha of 5% assuming 20% attrition or 90% power at a 2-sided alpha of 5% allowing for 16% attrition. Given that the e-coachER intervention is being delivered at the level of the individual participant, clustering has not been factored into this revised sample size calculation.

12.2 Statistical analysis

All analyses will be carried out using a detailed *a priori* statistical analysis plan that will be completed and agreed with the TMG and DMC prior to closure of the trial database and the commencement of any data analysis.

Analyses will be reported in full and in accord with CONSORT reporting guidelines (Schultz et al, 2010). Recruitment, intervention and control uptake, outcome completion rates and drop out will be reported (with 95% CIs) as a flow diagram and we will describe baseline participant characteristics in the two trial arms.

The primary analysis will compare the primary and secondary outcomes between intervention and control arms groups according to the principle of intention to treat (i.e. according to original randomised allocation) at twelve months adjusting for baseline outcome values and stratification and minimisation variables (recruitment site, postcode, age gender, and disease indication using logistic regression.

Secondary analyses will be undertaken to compare groups at follow up across all follow up points (i.e. four and twelve months) using a repeated measures approach. In addition, we will seek to undertake secondary per protocol analyses to examine the impact of different levels of the adherence to the e-coachER intervention. Pre-defined definitions of per-protocol will be agreed by the TMG and included in the statistical analysis plan.

The primary analysis model will be extended to fit interaction terms to explore possible subgroup differences in intervention effect in stratification and minimisation variables and the pre-defined baseline characteristics. As not formally powered, these subgroup analyses will be regarded as

exploratory and hypothesis-generating. Sensitivity analysis, making different assumptions about the imputation model used will be conducted for both primary and secondary analyses to assess the likely impact of missing data.

Contemporary mediational analysis methods (Emsley et al, 2010) will be used to explore the impact of process outcomes identified in the planned intervention components, including e-coachER engagement, use of behaviour change techniques, and motivation and processes of change (e. g., self-efficacy, autonomy, relatedness).

No interim analysis of primary or secondary outcomes is planned.

Models will be fitted using mixed effects regression models and undertaken in STATA v12.

12.3 Interim analysis

Once the recruitment period has finished, descriptive blinded analysis will be undertaken on the baseline data.

12.4 Economic evaluation

The economic analysis will include NHS, personal social services and patient perspective (NICE, 2012), with two approaches:

12.4.1 Within-trial-based analysis

Resource use data will be used to determine an incremental cost per Quality-Adjusted Life-Year (QALY: based on EQ-5D-5L). Resource use data will be collected via follow-up surveys at four and twelve months, and by e-mail to capture ERS uptake and engagement. Unit costs will be taken from the NHS reference costs (e.g. DH, 2012), standard unit costs (e.g. PSSRU, 2011), and published literature. QALYs will be estimated over the trial period for individual patients using an ‘area under the curve’ approach. It will also be possible to present the results in the form of a cost-consequence analysis (disaggregated costs next to the important outcomes). Descriptive analyses will show mean total costs and mean utilities by trial arm and differences between trial arms. Non-parametric bootstrapping will be used to estimate differences in mean costs, with 95% confidence intervals, and incremental cost-effectiveness ratios. Uncertainty will be represented in cost-effectiveness acceptability curves (CEACs) and incremental net benefits for the intervention arm versus control.

12.4.2 Beyond trial modelling

A decision analytical model will be used to examine the impact of PA on lifetime risk of developing a series of conditions which are known to be associated with physical activity and for which more robust quantifiable evidence is available (CHD, stroke and type II diabetes, potentially depression- with DH work- EMPHASIS model underway) following extensive previous work (Anokye et al, 2011, Anokye et al, 2014). Costs and QALYs will be discounted at the NICE recommended rates of 1.5% p.a. The modelling approach will be informed by new developments in the field, particularly the EMPHASIS model, which is being developed at Brunel (involves Anoyke) and expected to be completed in 2017.

13 DATA HANDLING

13.1 Subject numbering

Each participant will be allocated a unique study number following receipt of the reply slip (or telephone call or email equivalent) indicating interest in the study, and completion of baseline assessments (including accelerometer), and will be identified in all study-related documentation by their study number and initials. A record of names, addresses, telephone numbers and email addresses linked to participants' study numbers will be stored securely on the study database for administrative purposes.

13.2 Data collection

Data will be recorded on study specific data collection forms (CRFs), usually by the Research Assistant. Participants will complete participant-reported outcome measures. Data will be collected on paper for both study arms, with additional data collected from the e-coachER intervention (via the LifeGuide software platform) for intervention participants. An e-mail will be sent to participants at 4 weeks with a request for information on the number of sessions held with an exercise professional as part of the ERS, will request a response to indicate ERS uptake. All persons authorised to collect and record study data at each site will be listed on the study site delegation logs, signed by the relevant PI.

13.3 Data handling and record keeping

Completed CRFs will be checked and signed at the research sites by the research assistant or another member of the research team before being sent to the Pen CTU. Original CRF pages and questionnaires will be posted to the CTU at agreed timepoints with copies of the CRF retained at the relevant study site. Forms will be tracked using a web-based study management system. All data will be double-entered by the CTU on to a password-protected database. Double-entered data will be compared for discrepancies using a stored procedure and discrepant data will be verified using the original paper data sheets. Incomplete, incoherent, unreadable or other problem data in the CRF pages will be queried by the CTU with study site staff during data entry to ensure a complete and valid dataset. Questionnaire data will not be queried with participants. The CTU may complete further validation of data items, perform logical data checks and raise further data queries after data collection has been completed. The final export of anonymous data will be transferred to statisticians for analysis after all data cleaning duties have been performed by the CTU, this will usually be via email or a removable storage device. Identifiable information will not be exported from the study database as part of the final export.

Accelerometers will be received by the PenCTU and data will be downloaded via GENEActiv software, and linked to participant ID numbers. Files will be checked before the accelerometers are recirculated. Files will be then further analysed with bespoke software to classify data into levels of physical activity intensity using accepted cut-points. Standard operating procedures will be applied to make adecision about dealing with missing data. Selected primary and secondary accelerometer derived outcomes will be merged into an individual participant data set, and securely stored as below.

13.4 Data confidentiality and security

The research team will ensure that participants' anonymity is maintained on all documents. Data will be collected and stored in accordance with the Data Protection Act 1998/General Data Protection Regulation 2018.

Electronic study records will be stored in a SQL server database, stored on a restricted access, secure server maintained by Plymouth University. Data will be entered into the database via a bespoke web-based data entry system encrypted using SSL. Access to electronic data will be permission based,

with access to identifiable information limited to those processing questionnaires and performing initial screening activities. Data entered onto the database will be backed up according to PenCTU SOPs.

Within the CTU, anonymised paper-based study data will be stored in locked filing cabinets within a locked office. Any paper-based participant related identifiable data will be stored separately from the study data. Copies of study data retained at study sites will be securely stored for the duration of the study prior to archiving.

13.5 Access to data

The CTU data team will have access to the full dataset, including identifiable data. Site based researchers will have access to the dataset for participants from their site, including identifiable information, to perform screening activities. Other members of the study team and the CTU will have restricted access to anonymised study data. Access will be granted to the Sponsor and host institution on request, to permit study-related monitoring, audits and inspections. Access to the database will be overseen by the CTU data manager and trial manager.

13.6 Archiving

Following completion of data analysis and submission of the end of study report, the Sponsor will be responsible for archiving the study data and essential documentation in a secure location for a period of five years after the end of the trial. No trial-related records should be destroyed unless or until the Sponsor gives authorisation to do so.

14 MONITORING, AUDIT & INSPECTION

A trial monitoring plan will be developed and agreed by the TMG based on a risk assessment. This will involve central data monitoring but may also include on-site monitoring by the CTU trial manager. The Principal Investigators will be required to permit the CTU trial manager or deputy to undertake such monitoring as required to ensure compliance with the approved trial protocol and applicable SOPs, providing direct access to source data and documents as requested.

15 ETHICAL AND REGULATORY CONSIDERATIONS

15.1 Research Ethics Committee (REC) review & reports

The study will be undertaken subject to appropriate Research Ethics Committee (REC) approval and local NHS Research & Development approvals. The trial will be conducted in accordance with the protocol, the principles of the Declaration of Helsinki and ICH GCP. Any amendments of the protocol will be submitted to the Sponsor and REC for approval.

Substantial amendments that require review by REC will not be implemented until the REC grants a favourable opinion and the amendment has been reviewed by relevant NHS R&D departments as required. All correspondence with the REC will be retained in the Trial Master File and Investigator Site Files. An annual progress report will be submitted to the REC within 30 days of the anniversary date on which the original favourable opinion was given, and annually until the trial is declared ended. If the study is ended prematurely, the Chief Investigator will notify the REC, including the reasons for the premature termination. Within one year after the end of the study, the Chief Investigator will submit a final report with the results, including any publications/abstracts, to the REC.

15.2 Protocol compliance

Protocol deviations will be monitored by the CTU and reported to the Chief Investigator and Sponsor as appropriate. Significant deviations from the protocol which frequently recur are not acceptable and may potentially be classified as a “serious breach”.

15.3 Notification of serious breaches of GCP and/or the protocol

A “serious breach” is a breach of the protocol or of the conditions or principles of Good Clinical Practice which is likely to effect to a significant degree –

(a) the safety or physical or mental integrity of the subjects of the trial; or
(b) the scientific value of the trial

The Sponsor will be notified immediately of any case where the above definition applies during the trial period. The Sponsor is responsible for notifying the REC of a serious breach in any study within seven days of the matter coming to their attention.

15.4 Indemnity and insurance

The University of Plymouth (as research sponsor) and its research collaborators will be required under the terms of their collaboration agreement to maintain public liability, professional indemnity and employer's liability insurance (together with such other insurance as the sponsor may require from time to time) to cover liabilities arising from the study.

In addition, each party is required under their collaboration agreement to indemnify the other parties and their staff against all claims, proceedings, liabilities, losses and costs incurred by them as a result of or in connection with the indemnifying party’s negligent acts or omissions, negligent delivery of its work under the study, negligent performance or breach of its obligations under the agreement, wilful misconduct or breach of statutory duty (including liability for damage to property, injury or death caused by any such negligent act, omission or wilful misconduct).

All participants taking part in the exercise referral scheme will be covered in case of harm by the relevant exercise provider’s public liability, professional indemnity and premises insurance.

16 DISSEMINATION POLICY

We will use newsletters to maintain contact with participants throughout the trial. At the end of the trial, the study team will prepare a plain English summary of the main study results (comparing the two trial arms) which will be sent by e-mail or post to study participants. The research team will work with stakeholders at each site, and nationally, to help to interpret the results and the implications for policy and practice. Dissemination may involve presentation at meetings of relevant support groups or other lay audiences, as well as NHS strategy forum at local and national level.

There will be a standing item on the agenda for each Project Management Group meeting (quarterly) on the publication plan and establishing authorship rules. We shall aim to submit the trial Protocol for publication no later than the end of the 3 month internal pilot phase of the study. Reports will comply with current CONSORT guidelines for publishing randomised trials (http://www.consort-statement.org/) and TIDieR guidelines for intervention reporting (http://www.equator-network.org/reporting-guidelines/tidier/). The study results will be submitted for publication in relevant international, high impact, peer reviewed journals. Names of key collaborators and groups who have contributed to the trial will be clearly stated in all publications. The study findings will be presented at regional, national and international meetings as appropriate.

17 REFERENCES

Anokye N et al (2011). The cost-effectiveness of exercise referral schemes. BMC Pub Health, 11:954.

Anokye N et al et al (2014). Is brief advice in primary care a cost-effective way to promote physical activity. Br J Sp Med, 48, 202-206.

Benaissa M et al et al (2012). Tele-healthcare for diabetes management: A low cost automatic approach. Conf Proc IEEE Eng Med Biol Soc. twelve90-3.

BHF (2010). A Snapshot of ER Schemes Operating in England, Scotland & Northern Ireland - 2006-2008. British Heart Foundation Final Report, 2010. Chalder M et al (20twelve). Facilitated physical activity as a treatment for depressed adults: randomised controlled trial. BMJ, 344, e2758

Bouchard C, et al (2015). Less sitting, more physical activity, or higher fitness? Mayo Clin Proc., 90(11):1533-40.

Campbell F, et al. (2015). A systematic review and economic evaluation of exercise referral schemes in primary care: a short report. Health Technol Assess. 19(60):1-110.

Compernolle S, et al. (2015). Effectiveness of a web-based, computer-tailored, pedometer-based physical activity intervention for adults: a cluster randomized controlled trial. J Med Internet Res. 9;17(2):e38.

Davies CA, et al. (2012). Meta-analysis of internet-delivered interventions to increase physical activity levels. Int J BehavNutr Phys Act. 9:52.

Devi R, et al. (2015). Internet-based interventions for the secondary prevention of coronary heart disease. Cochrane Database Syst Rev. 12:CD009386.

DH (2011). Start Active, Stay Active: A report on physical activity from the four home countries' Chief Medical Officers. London Depart of Health.

DH. NHS Reference costs (2011/12). London: Depart of Health, 2012.

Dunstan DW et al (2005). Home-based resistance training is not sufficient to maintain improved glycemic control following supervised training in older individuals with type 2 diabetes. Diab Care, 28, 3-9.

Emsley R et al (2010). Mediation and moderation of treatment effects in randomised controlled trials of complex interventions. Stat Methods Med Res. 19, 237-70.

Greaves CJ et al (2011). Systematic review of reviews of intervention components associated with increased effectiveness in dietary and physical activity interventions. BMC Pub Health, 18, 119.

Harris T, et al. (2015). A primary care nurse-delivered walking intervention in older adults: PACE (pedometer accelerometer consultation evaluation)-Lift cluster randomised controlled trial. PLoS Med. 12(2):e1001783.

Joseph RP, et al. (2014). Internet-based physical activity interventions. Am J Lifestyle Med. 8(1):42-68.

Larsen B, Gilmer T, Pekmezi D et al (2015). Cost effectiveness of a mail-delivered individually tailored physical activity intervention for Latinas vs. a mailed contact control. Int J of Beh Nutr & Phys Act, 12:140

Lloyd S et al (2013). Losing weight online with POWeR: a randomised controlled trial of a web-based behavioural intervention in a community setting. Lancet, 382, S62.

Michie S et al (2009). Effective techniques in healthy eating and physical activity interventions: a meta-regression. Health Psychol, 28, 690-701.

Morrison LG, et al, Yardley L. (2014). Understanding usage of a hybrid website and smartphone app for weight management: a mixed-methods study. J Med Internet Res. 16(10):e201.

Newman MG et al (2011). A review of technology-assisted self-help and minimal contact therapies for anxiety and depression: is human contact necessary for therapeutic efficacy? Clin Psychol Rev., 31, 89-103.

NICE (2008a). Type 2 diabetes: The management of type 2 diabetes.

NICE (2008b). Type 2 diabetes: The management of type 2 diabetes. Osteoarthritis: The care and management of osteoarthritis in adults.

NICE (2009). Depression: The treatment and management of depression in adults.

NICE (2010). Obesity: Guidance on the prevention, identification, assessment and management of overweight and obesity in adults and children.

NICE (2011). Hypertension: Clinical management of primary hypertension in adults.

NICE (2012). Methods for the development of NICE public health guidance.

Pavey TG et al (2012). Levels and predictors of exercise referral scheme uptake and adherence: a systematic review. J of Epid Comm Health, 66, 737-44.

Pavey TG et al (2011a). Clinical effectiveness and cost-effectiveness of exercise referral schemes. HTA Report.

Pavey, T.G., et al. (2011b). Effect of exercise referral schemes in primary care on physical activity and improving health outcomes: systematic review and meta-analysis. British Medical Journal. 343: d6462.

PSSRU (2011) Unit costs of health & social care. Kent: Personal Social Services Research Unit. , University of Kent, 2011.

Rouse PC et al (2011). In the beginning: role of autonomy support on the motivation, mental health and intentions of participants entering an exercise referral scheme. Psychol Health., 26, 729-49.

Schulz KF et al (2010). CONSORT 2010 statement: updated guidelines for reporting parallel group randomised trials. BMJ. 340, c332.

Warburton DE, Bredin SS. (2016). Reflections on physical activity and health: What should we recommend? Can J Cardiol. 32(4):495-504.

Wijsman CA, et al. (2013). Effects of a web-based intervention on physical activity and metabolism in older adults: randomized controlled trial. J Med Internet Res.15(11):e233.

Williams S et al (2013). A qualitative case study of LifeGuide: Users' experiences of software for developing Internet-based behaviour change interventions. Health Info J, 19, 61-75.

Yardley L, et al. (2014). Randomised controlled feasibility trial of a web-based weight management intervention with nurse support for obese patients in primary care. Int J BehavNutr Phys Act. 21;11:67.

Yardley L et al (2010). Understanding reactions to an internet-delivered self-care intervention: accommodating user preferences for information provision. BMC Med Info & Decision Making, 10, 52.

Yardley L (2011). The multidisciplinary future of e-health interventions: Learning from LifeGuide. Psych & Health, 26(suppl. 2): 352-353.

18. APPENDICES

18.1 Appendix 1: e-coachER indicative intervention framework

Sequential process	Performance objectives	Behaviour Change Techniques<br><br>(Michie et al., 2013)	Implementation Strategy
Welcome Pack and pedometer (print) & User Guide.<br><br>Introduction to web-based support for self-directed PA	To introduce the user to the philosophy of the website to become personal coach<br><br>Build on personal support provided by ERS using web-based platform<br><br>Support those who don't want to /can't engage with ERS personnel<br><br>Support achievement of personal goals for PA to enhance health	*N/A*	Explain philosophy of using website to become own personal coach.<br><br>Links provided to local services and other self-help resources to highlight patient autonomy and choice.<br><br>Offers e-coachER facilitator to help with using technology. Provide link to IT support in Southampton.
Step 1 - Thinking about the benefits of physical activity	Elevate importance of physical activity	*82. Information about health consequences*<br><br>*83. Information about emotional consequences*	Quiz to engage participants using positive framing.<br><br>Provide evidence of multiple benefits of PA especially for relevant health condition(s).<br><br>Elicit and address concerns about PA, describing support given as part of ERS and by website.
Step 2: Support to get active	To encourage user to access and create social support networks<br><br>To encourage user to take advantage of exercise referral scheme	*1.Social support (practical)*<br><br>*2.Social support (emotional)*<br><br>*3.Social support (unspecified)*	Explain how to make the most out of the ERS support to learn how to become own personal trainer in future.<br><br>Explain how user can create a personal 'PA challenge' and share it with family, friends, peers, and exercise and health professionals. The patient may be encouraged to tell others about how e-coach has been used to support behaviour change.<br><br>Suggest ways of involving family or friends in longer-term support for continued PA.<br><br>Link to online sources of local support (e.g., local walking or jogging group, or British Trust for Conservation Volunteers).<br><br>How to use website to send personalised email/text reminders, motivational messages.<br><br>Draw on positive normative beliefs; identify benefits of social interaction (companionship). Sharing personal PA challenge with others, involve friends and family, online local support links.<br><br>Identify benefits of informational support (from ERS scheme) in addition

			to emotional support from family and friends)
Step 3: Counting your steps	To educate and support the user to monitor step counts using a pedometer over a week. Emphasise personal experimentation	*10. Self-monitoring of behaviour*	Provide guidance on how to count steps/use pedometer.<br>Provide guidance on how steps can be implemented into lifestyle.<br>Encourage self-monitoring using diary.
Step 4: Making your step plans	To set explicit step count goals for the following week	*66. Goal setting (behaviour)*	Give rationale and evidence for goal-setting for graded increase in PA.<br>User sets specific, achievable goals for next week (e.g. sessions completed, step count using the supplied pedometers).<br>Links provided to local services and other resources.
Step 5: Making your activity plans	To educate and support the user to identify behavioural goals (types of activities).	*68. Action planning*	User selects walking or 'other physical activities' (which includes options for facility-based activity with practitioner support within ERS).<br>Present options for facility and lifestyle-based activity.<br>Sets specific, achievable goals for next week with a particular focus on avoiding days with less activity by planning walking or other activities.<br>Keeping a PA diary.
Weekly goal and PA review	To promote adherence and graded increase in PA by providing tailored feedback and advice based on self-reported goal progress.	*66. Goal setting behaviour*<br>*68. Action planning,*<br>*69. Review behaviour goals.*	User records extent to which goals achieved in previous week, gets progress graph and personalised feedback:<br>Praise for any goal achievement, encouragement to set more challenging goal if not yet meeting target PA criteria.<br>Encouragement where goals not attained, with links to webpages to assist with increasing motivation or confidence, selecting different activities or goals, making better plans, accessing support, overcoming setbacks (with links to relevant sessions below).<br>Each session completed ends with new links to reputable information and resources (e.g. NHS Choices, condition-specific PA advice websites).<br>Help user plan gradual increases in PA.
Step 6 – Finding ways to achieve your plans	To help the user harness their environment to provide support for PA	*30. Restructuring the physical environment*	Make plan to use environment to automatically support PA (with examples e.g. fitness equipment in

	Identifying personal motivations, building confidence.	*31. Restructuring the social environment* *32. Avoidance / reducing exposure to cues for behaviour*	living room, route to work/shops that involves more PA, committing self to specific routine).<br>Advise user on how to use website to send personalised email/text reminders, motivational messages.<br>Overcoming barriers in work, leisure, home and travel. Building self-efficacy.<br>Using smart phone apps for mobile support (e.g. PowerTracker, MyFitnessPal)<br>Invite user to identify personal motivations for becoming more active.
Motivational Messages (text or/and emails)	To provide reminders of users personal reasons (not necessarily health reasons) for becoming more active	*15. prompts/cues*	Invite user to write motivational message to be sent weekly or monthly detailing their own motivations for becoming more active
Step 7 – Dealing with setbacks	To provide strategies for overcoming relapse in levels of PA.	*5. Reduce negative emotions*	Identify possible causes of relapse (e.g., illness, holidays, change in work hours, new caring responsibilities) and plan ways to overcome barriers.<br>Challenging catastrophic negative thoughts about lapses from intended PA.<br>How to learn from a lapse and plan to avoid or overcome in future.<br>Provide salient role models of people overcoming barriers to successfully engage with PA.

Taylor A, *et al. Br J Sports Med* 2021; 55:444–450. doi: 10.1136/bjsports-2020-103121
